# Adaption to glucose limitation is modulated by the pleotropic regulator CcpA, independent of selection pressure strength

**DOI:** 10.1186/s12862-018-1331-x

**Published:** 2019-01-10

**Authors:** Claire E. Price, Filipe Branco dos Santos, Anne Hesseling, Jaakko J. Uusitalo, Herwig Bachmann, Vera Benavente, Anisha Goel, Jan Berkhout, Frank J. Bruggeman, Siewert-Jan Marrink, Manolo Montalban-Lopez, Anne de Jong, Jan Kok, Douwe Molenaar, Bert Poolman, Bas Teusink, Oscar P. Kuipers

**Affiliations:** 1 Molecular Genetics Group, University of Groningen, Nijenborgh 7, 9747 AG Groningen, The Netherlands; 2 Department of Biochemistry, University of Groningen, Nijenborgh 4, 9747 AG Groningen, The Netherlands; 3 Kluyver Center for Genomics of Industrial Fermentations/NCSB, Julianalaan 67, 2628 BC Delft, The Netherlands; 4 Systems Bioinformatics, Faculty of Earth and Life Sciences, VU University Amsterdam, De Boelelaan 1085, 1081 HV Amsterdam, The Netherlands; 5 Molecular Microbial Physiology Group, Faculty of Life Science, Swammerdam Institute of Life Sciences, University of Amsterdam, Science Park 904, 1098 XH Amsterdam, Netherlands; 6 Molecular Dynamics Group, University of Groningen, Nijenborgh 7, 9747 AG Groningen, The Netherlands; 7 Present address: DSM Biotechnology Centre, Alexander Fleminglaan 1, 2613 AX Delft, The Netherlands; 8 Present address: Chr. Hansen, Boege Allé 10-12, 2970 Hoersholm, Denmark

**Keywords:** Evolution, Systems biology, Lactic acid bacteria

## Abstract

**Background:**

A central theme in (micro)biology is understanding the molecular basis of fitness i.e. which strategies are successful under which conditions; how do organisms implement such strategies at the molecular level; and which constraints shape the trade-offs between alternative strategies. Highly standardized microbial laboratory evolution experiments are ideally suited to approach these questions. For example, prolonged chemostats provide a constant environment in which the growth rate can be set, and the adaptive process of the organism to such environment can be subsequently characterized.

**Results:**

We performed parallel laboratory evolution of *Lactococcus lactis* in chemostats varying the quantitative value of the selective pressure by imposing two different growth rates. A mutation in one specific amino acid residue of the global transcriptional regulator of carbon metabolism, CcpA, was selected in all of the evolution experiments performed. We subsequently showed that this mutation confers predictable fitness improvements at other glucose-limited growth rates as well. In silico protein structural analysis of wild type and evolved CcpA, as well as biochemical and phenotypic assays, provided the underpinning molecular mechanisms that resulted in the specific reprogramming favored in constant environments.

**Conclusion:**

This study provides a comprehensive understanding of a case of microbial evolution and hints at the wide dynamic range that a single fitness-enhancing mutation may display. It demonstrates how the modulation of a pleiotropic regulator can be used by cells to improve one trait while simultaneously work around other limiting constraints, by fine-tuning the expression of a wide range of cellular processes.

**Electronic supplementary material:**

The online version of this article (10.1186/s12862-018-1331-x) contains supplementary material, which is available to authorized users.

## Background

Studies of microbial evolution can be used to understand the adaptive mechanisms that result in fitter genotypes in response to different selective pressures. Laboratory evolution offers unique opportunities for the testing of predictions made by population geneticists and evolutionary biologists, whilst monitoring evolving populations under controlled, and well-defined, conditions. Microorganisms are appealing to be used for evolutionary studies due to their fast growth rate, controllable population size and the availability of advanced molecular biology tools. Moreover, for many microorganisms detailed knowledge exists about operon organization, transcription regulation and physiology such that detailed molecular understanding of the adaptive processes can be obtained.

Laboratory evolution of microorganisms can be carried out under a broad range of selective pressures, such as varying growth rate in serial batch cultures [1, 2], antibiotic resistance in different cultivation settings [3], nutrient limitation in chemostats [4], cell number in emulsion-based systems [5], and growth rate in dynamic environments [6]. Such studies have revealed remarkable evolutionary versatility of microorganisms to adapt to different selective pressures.

In this study, we exploited the chemostat to perform laboratory evolution. The chemostat presents an ideal system to study two basic aspects regarding the versatility of microbial evolution: plasticity and evolvability.

Evolutionary plasticity is related to the existence of alternative mutations that give rise to a comparable fitness enhancement for a particular selective pressure. Plasticity is high when many alternatives exist, and tends to zero when only a single option subsists. In the chemostat, plasticity can be assessed across a wide range of strengths for the same selection pressure. This can be achieved by replicate experiments testing for fixation of alternative mutations at multiple dilution rates.

Evolvability considers the rate of evolution. It is both related to the number of mutations required, and their fixation rate, to reach a specific quantitative change in fitness. If several mutations are required in sequence for a specific quantitative change in fitness then evolvability is low, and vice-versa.

Until now, microbial evolution in chemostats has been limited by the number of replicates and by the number of generations and volume changes. The chemostat was initially described as a device that could continuously cultivate bacteria for an indefinite period of time [7]. However, to date the longest published cultivation using a chemostat is only a few hundred generations [4], despite recent advancements in the development of dedicated multiplex culture systems [8]. This is several orders of magnitude shorter than the longest serial batch cultivation experiments – > 50,000 generations and counting [1]. To address the lack of long-term continuous cultivation, we developed a versatile bioreactor cap and experimental set-up to perform parallel, prolonged cultivations under chemostat conditions, at small working volumes. These advances allowed us to conduct experiments in quadruplicate for more than 1350 consecutive generations.

We studied the microbial evolution of *Lactococcus lactis* in glucose-limited chemostats. This well-characterized lactic acid bacterium (LAB) exhibits a simple fermentative metabolism and is an ideal model organism to investigate broader evolutionary principles. It carries a small genome (about 2.5 Mb) and several strains isolated from different niches have been fully sequenced, allowing for a good understanding of the natural evolution of this bacterium [9–12]. We describe a laboratory evolution experiment in chemostats at two different dilution rates with 4 replicates each. The mutations were identified and their molecular consequences characterized. We elucidated the fitness enhancement using complementary computational and experimental approaches. Remarkably, we found the same mutation in experimental replicates across the dilution rates tested. We further tested experimentally our in-silico simulations by carrying out chemostat competition experiments at two additional dilution rates, and found that this mutation was also advantageous then.

This study indicates that the fitness landscape of this microbial evolution process is likely single-peaked and insensitive to the strength of the selection pressure. This suggests that the adaptation process from batch cultivation to constant conditions set by glucose limitation is characterized by high evolvability and low plasticity.

## Results

### Chemostat design and cultivation conditions for long-term microbial evolution under stable environmental conditions

**Fig. 1:**
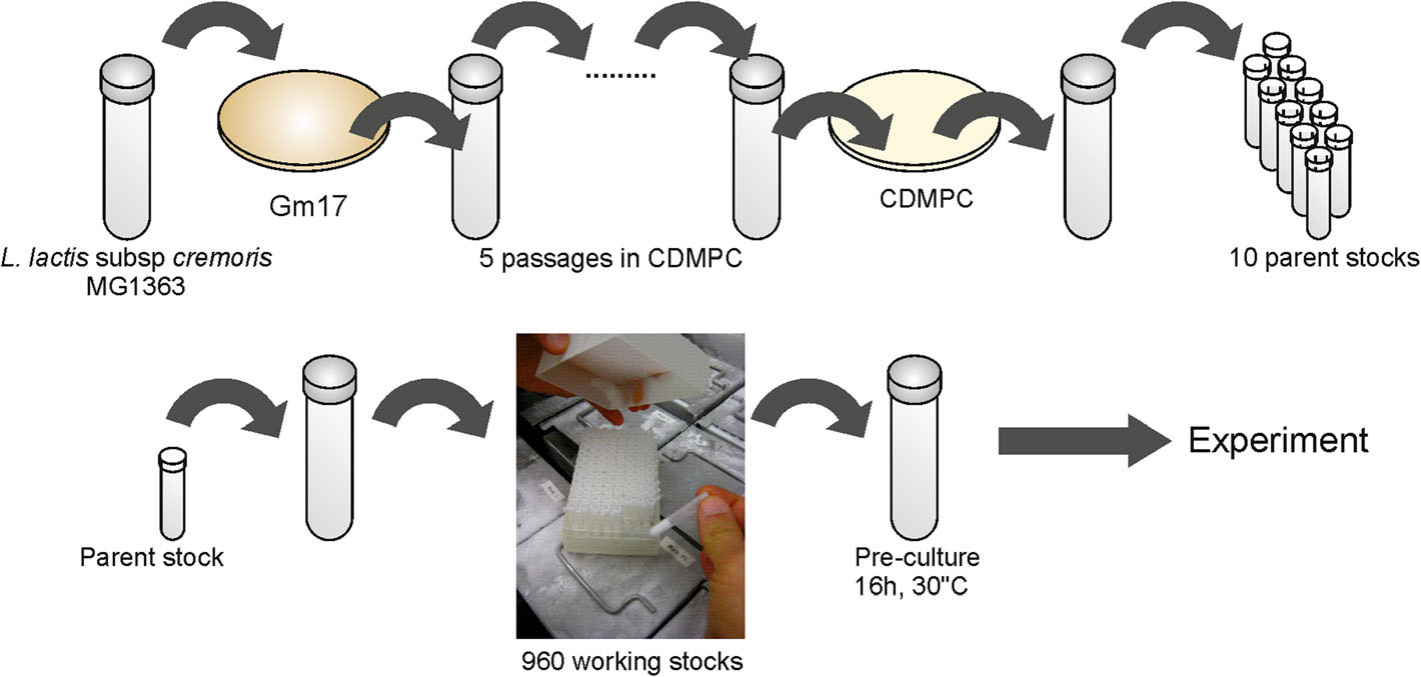
Standardized cryopreservation procedure to minimize strain variability among experiments. GM17: M17 medium supplemented with 25 mM glucose, CDMPC: chemically defined medium for prolonged cultivation

A requirement of laboratory evolution is that the technical variability between replicates should be minimized. Our initial focus was to carefully define the cultivation conditions (chemostat and medium) and to standardize the experimental procedures (inoculation, sampling, and cell storage). We developed a chemically defined medium particularly suited for prolonged cultivation (CDMPC) based on the nutrient requirements [13] and biomass composition of *L. lactis* MG1363 [14] (detailed in Additional file 1 and Additional file 2: Table S1). The major differences compared to previously published chemically defined media for *L. lactis* [13, 15, 16] are (i) the implementation of standardized procedures that reduce variations between media batches, (e.g. those caused by poor solubility of metal co-factors); (ii) the removal of components that cause technical (downstream) difficulties or perturbation of cell behavior; and (iii) the finetuning of the concentration of medium compounds to meet the nutrient requirements of *L. lactis*. To reduce the variability introduced by the inoculation procedure, we implemented a strictly controlled cryopreservation and inoculation procedure described in Additional file 1 (Fig. 1).

The cultivation history of bacterial strains is of upmost importance. Strains can rapidly deviate from their original genotype, which can sometimes lead to noticeable phenotypic differences [2, 17]. Our initial inoculum came from the original *L. lactis* MG1363 stock, for which the genome sequence is published [17]. We wanted to ensure that all the prolonged cultivations would be seeded from the same starting inoculum as much as possible, so we implemented a standardized cryopreservation and inoculation procedure (detailed in Additional file 1). In order to ensure standardized growth in the newly developed medium, we performed a short-term serial batch adaptation of an isolate of this strain to the medium CDMPC. The resulting single isolate picked (Genr0) was cryopreserved and used as the seeding population for all the laboratory evolution experiments performed (Fig. 1).

We aimed at carrying out all the prolonged cultivations under tightly controlled conditions, while the cultures were left as unperturbed as possible, and without interruptions. We needed a chemostat set-up that would be (i) suitable to run parallel cultivations; (ii) robust enough to be stable for several hundreds of generations; and (iii) with a relatively small working volume to limit the amount of medium needed to a manageable amount. Unfortunately, such set-ups are not commercially available such that our list of specifications is met. And although there are some tailored parallel continuous cultivation systems reported in literature (a very good example in [8]), we speculated that they would not offer the type of robustness that would be necessary (particularly, due to the tubing connectors used to the in- and outlet ports of the reactor). So to permit more robust parallel cultivations and reduce costs, we designed small-scale bioreactors made of welded aluminum especially suited and tested to meet these requirements (detailed in Additional file 1).

### Parallel laboratory evolution in chemostat cultures

**Fig. 2:**
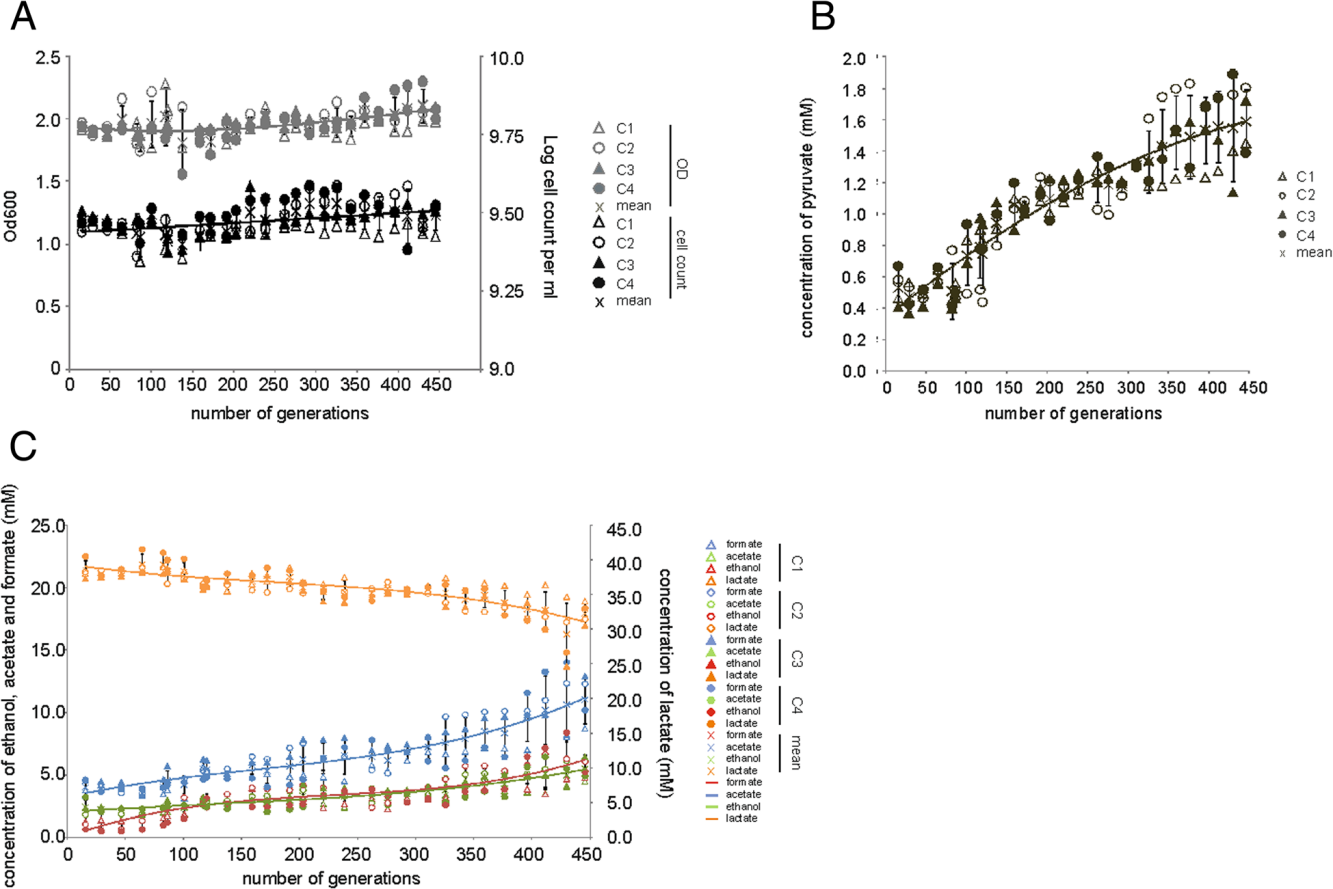
Strain characteristics during the evolution experiments at D of 0.5 h^− 1^ and of the resulting evolved strains. For each of the chemostats (C1, open triangles; C2, open circles; C3, closed triangles; and C4, closed circles) the biomass concentration and cell counts (**a**) along with organic acids (**c**) were measured. The values for lactate (orange), formate (blue), acetate (green) and ethanol (red) are shown. The levels of pyruvate increased in all the chemostats during the evolution experiment (**b**). Mean concentrations at each generation are shown by data symbol ‘X’, along with error bars representing standard deviation and a 3rd order polynomial fit solely used for the purpose of visualizing the overall trend

*L. lactis* MG1363 has a cultivation history that most likely resembles a serial batch with its growth rate varying with changes in nutrient availability. To investigate the evolutionary plasticity of this strain when adapting to constant environments, we allowed it to evolve under chemostat conditions i.e. with a constant growth rate. Genr0 was cultivated continuously in four replicates, each using 60 mL glucose-limited chemostats at a growth rate of 0.5 h^− 1^ without any kind of interruption. The experiment was stopped after 309 volume changes (calculated by multiplying the dilution rate by the cultivation time), which corresponds to 445 generations (determined by multiplying the doubling time (equals Ln2 divided μ) by the cultivation time). A growth rate of 0.5 h^− 1^ is equivalent to ~ 70% of the maximal growth rate in CDMPC for *L. lactis*. Culture samples were harvested periodically every 15–25 generations from the effluent to determine cell density and the fluxes of fermentation substrates and products (Fig. 2). Cell samples were stored as glycerol stocks at − 80 °C to be used as snapshots of the evolutionary process.

Throughout the prolonged cultivation, all four replicates (referred hereon as 445C1 through 445C4, referring to the number of generations) displayed very similar behavior. Biomass concentration did not vary significantly indicating that biomass yield on substrate remained constant (Fig. 2a). *L. lactis* can catabolize glucose through homolactic or mixed acid fermentation, excreting, respectively, lactate, or acetate, formate and ethanol [16]. At a dilution rate of 0.5 h^− 1^, Genr0 produces mainly lactate (lactate:acetate ratio close to 16). Throughout the course of the experiment, all parallel reactors shifted gradually towards mixed acid fermentation leading to a ratio of approximately 7 (Fig. 2c). Even though this fermentative mode leads to a theoretical 50% increase in ATP yield on substrate, the biomass concentration did not change, as evolved cells also excreted more pyruvate, the final shared precursor of the homolactic and the mixed acid fermentative pathways (Fig. 2b).

**Fig. 3:**
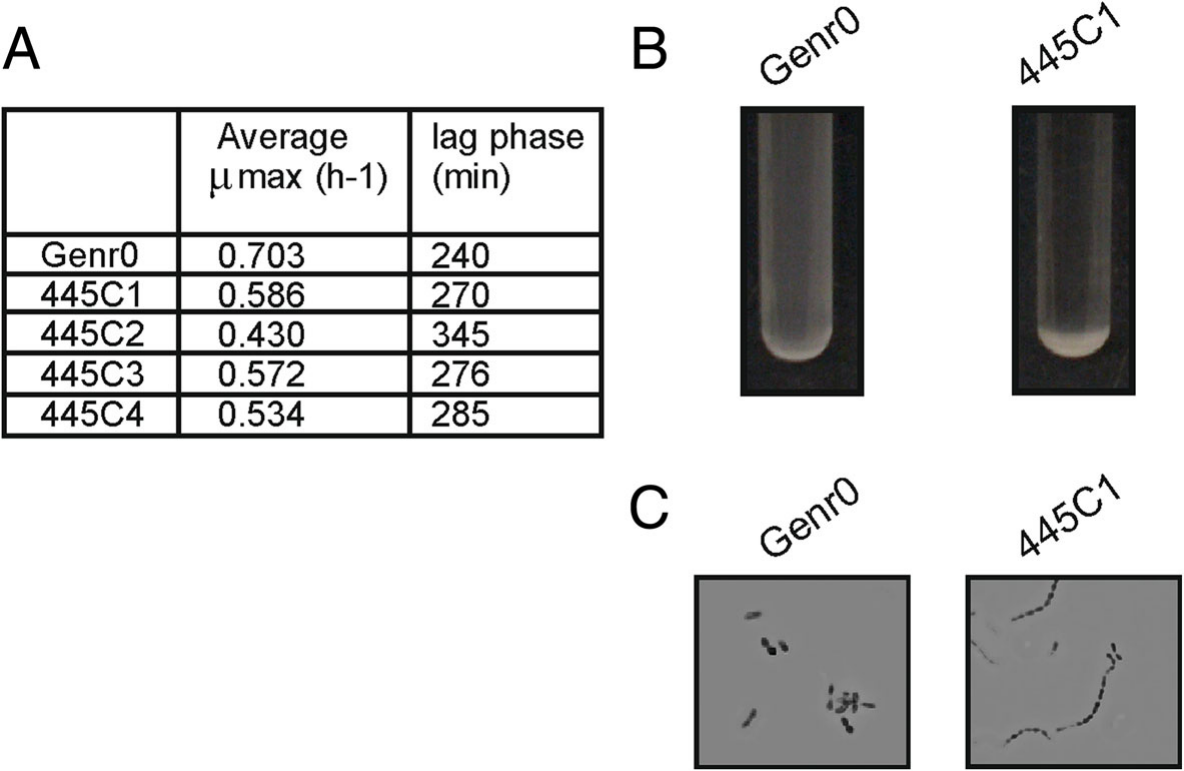
Phenotypic characterization of evolved strains in batch culture The evolved strains were revived in batch culture and the growth characteristics determined (**a**). **b** Evolved strains sediment during growth. Overnight cultures of Genr0 and 445C1 in CDMPC are shown. **c** Sedimentation is caused by the formation of long cell chains. Typical pictures are shown for Gen0 and 445C1 grown for 16 h in CDMPC. The cells were visualized using a Zeiss light microscope and a Zeiss digital camera. Magnification, × 1000

During batch cultivation, evolved cells differed phenotypically from Genr0. The differences included the growth rate, length of lag phase and sedimentation (Fig. 3). We compared to Genr0 both the population samples collected after 445 generations and single colony isolates from the evolved population samples (Additional file 1: Figures S1, S2, S3, S4 and S5). Prolonged cultivation under chemostat environment selected cells that cope poorly with high concentrations of glucose. Irrespective of whether the evolved populations or the isolates were compared, they were clearly outperformed by Genr0 in batch. Most notably, μ_max_ dropped to a value close to the dilution rate at which cells were evolved, which falls nearly 25% below the μ_max_ of the parent strain (Fig. 3). The evolved strains also exhibited extended lag phases and sedimentation during batch cultivations. The sedimentation was reminiscent of the AcmA-deficient phenotype previously described [18].

### Identification of the mutations arising during the evolutionary experiment

**Fig. 4:**
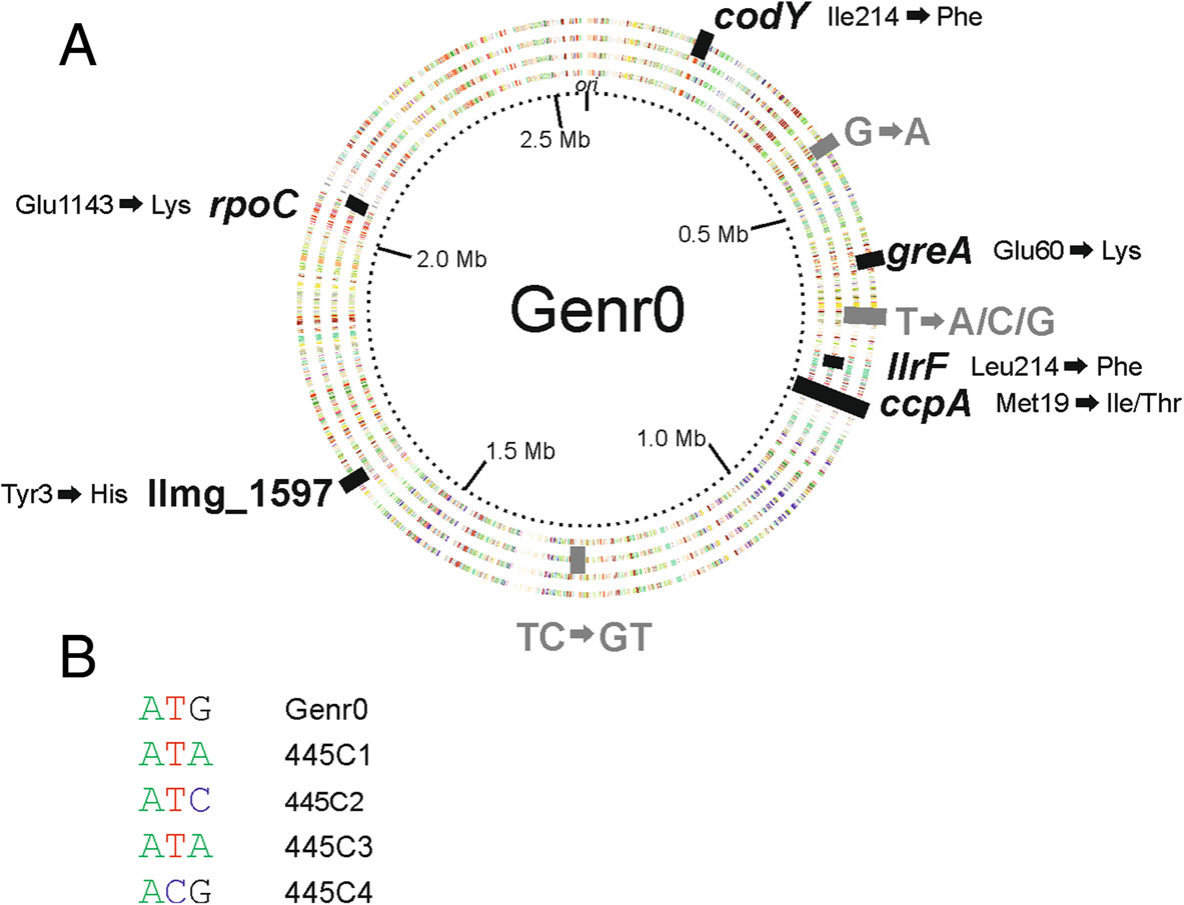
Mutations identified in the 4 evolved strains compared to the original strain Genr0. **a** The genes and intergenic regions with SNP mutations are shown. For coding regions (in black) the resulting amino acid substitution is indicated. The dotted inner ring indicates the genome position. The rings are color-coded according to the COG-classification of the gene present on the forward and reverse strands, and from inner to outer ring we represent evolved strains 445C1 to C4. **b** The DNA mutations in the codon for Met19 in CcpA are shown for each strain

We sequenced single-colony isolates from the original strain stock of Genr0 and from the end of the four replicate evolution experiments performed at a dilution rate of 0.5 h^− 1^ (i.e. 445C1, 445C2, 445C3, 445C4). Single isolates were chosen to keep the focus on the mutations, which gave rise to a selective advantage under the conditions tested. The population dynamics were determined after this (see section Population dynamics of prolonged cultivation experiments). The published whole genome sequence for *L. lactis* MG1363 [17] was used as a reference for sequence assembly. Mutations were identified and verified by Sanger sequencing (Additional file 3: Table S2). In comparison with Genr0, we did not find any evidence to support gene amplifications or large genomic rearrangements in the evolved strains. We did however identify several SNPs unique to the evolved strains (Fig. 4). The number of SNPs accumulated per genome was between one (445C1) and six (445C4). This corresponds to a mutation rate of 1.3–7.8 × 10^− 9^ (per base pair per generation) which is in line with currently published estimates [19].

All the SNPs in the coding regions were non-synonymous, and strikingly, all strains accumulated a mutation in the codon coding for Met19 in the global transcriptional regulator CcpA of Genr0. At the DNA level, the mutations differed between the evolved strains (Fig. 4). In strains 445C1, 445C2, and 445C3 ATG was mutated to either ATA or ATC, resulting in the amino acid change from Met to Ile. In none of the sequencing experiments performed for cells cultured at a D of 0.5 h^− 1^ was the Ile codon ATT identified (see also section on Population dynamics of prolonged cultivation experiments). For strain 445C4, the ATG codon was mutated to ACG resulting in a Met to Thr change. Strains 445C2, 445C3 and 445C4 contained a few additional mutations (more in details in Additional file 1), while the isolate of 445C1 only contained the SNP in *ccpA*.

Isolate 445C1 displays all of the common phenotypic differences observed between the evolved strains and Genr0. For example, these include the growth kinetics and sedimentation phenotype (Figs. 2 and 3) and, as addressed below, altered glucose utilization kinetics. Since 445C1 differs from Genr0 by only the CcpA Met19 to Ile substitution, and this mutation is widespread amongst the other biological replicates as well, we are led to assume that the latter is the genetic basis conferring the fitness advantage that has led to its fixation in all four independently evolved populations.

The sequences were also analyzed for insertions and/or deletions and when compared to the Genr0 sequence, the evolved strains showed no significant frameshifts. However, when the sequences of Genr0 and the evolved strains were compared to those published for *L. lactis* MG1363, all strains contained a mutation in *malR* which would results in a frameshift in the maltose operon transcriptional repressor MalR. This could be due to sequencing errors from adjacent C nucleotides or a truncated MalR protein. The truncation would result in a HTH DNA binding domain without a ligand binding domain. This is not related to the adaptation to constant glucose conditions of the chemostat since it was already observed in Genr0.

### Effect of CcpA M19I on global gene expression patterns

**Fig. 5:**
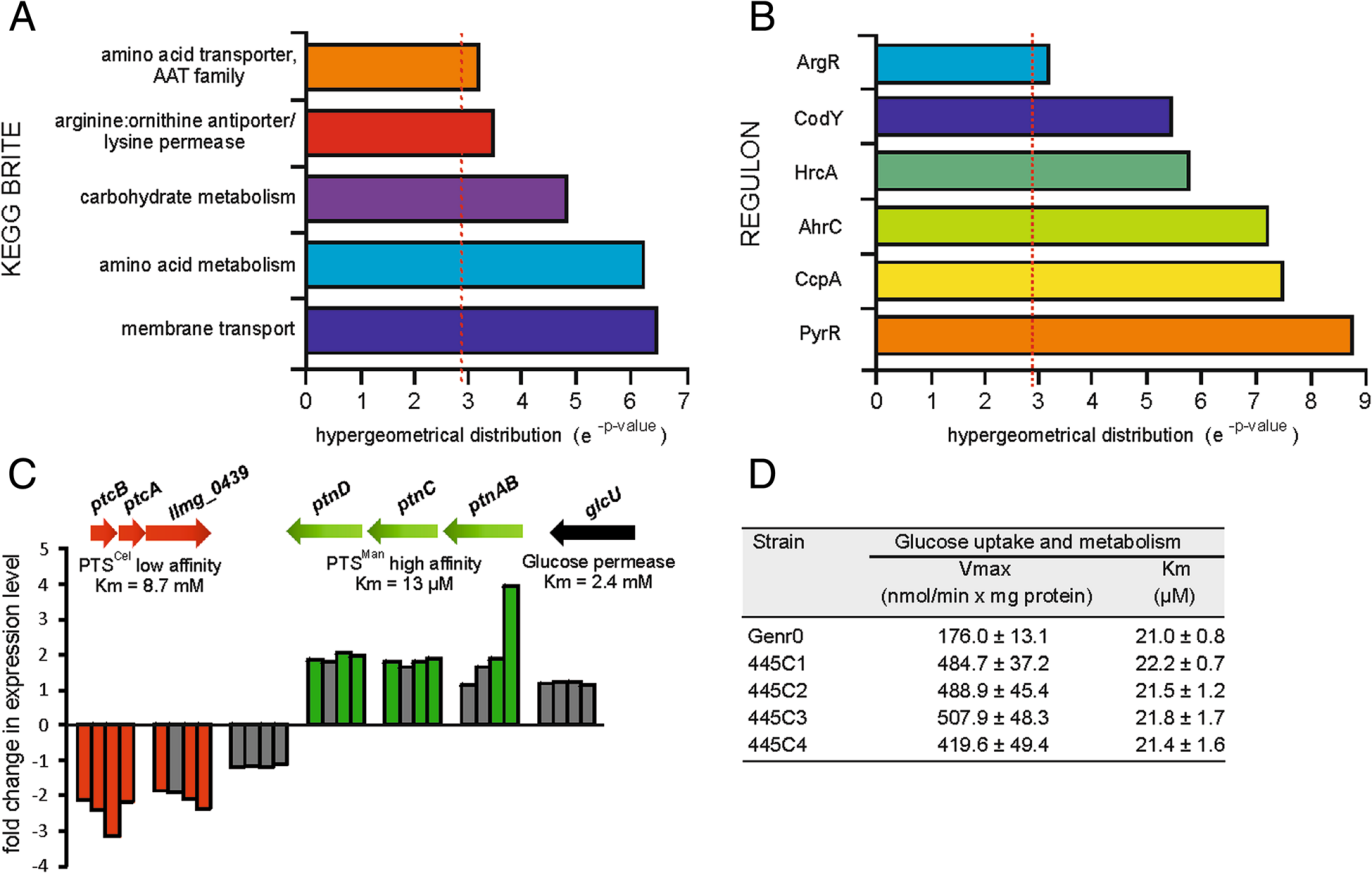
Altered gene expression in evolved strains is focused on transport pathways. Genes were considered to be significantly changes with a Bayes *p*-value score of less than 0.05 and a pfp value of less than 0.05. Genes found to change significantly in the evolved strains were grouped into functional classes. The p-value is the summation of the hypergeometrical distribution and *p*-values less than 0.05 were considered to be significant. **a** Over-represented KEGG BRITE classes found. **b** Over-represented regulons found. **c** The expression of genes involved in glucose uptake in *L. lactis* MG1363 were significantly changed in the evolved strains. Highlighted in green are genes that were up-regulated and in red for down-regulated. Gene expression changes not meeting the significance cut-off values are highlighted in grey. **d** Kinetic parameters of glucose transport in Genr0 and the evolved stains were determined in cells grown to exponential phase in CDMPC. Glucose transport was assayed with the use of [14-C]-labeled glucose. Values of three independent experiments were averaged and are reported ±SD. Vmax and Km were determined using glucose concentrations from 1.2 to 200 μM

The evolved strains were revived in CDMPC and grown until mid-exponential phase at which point microarray analysis was performed [20]. Gene expression in the evolved strains was compared to that of Genr0. Evolved strain 445C1, which contains only the SNP in *ccpA*, showed an altered gene expression dominated by genes involved in membrane transport, especially carbohydrate import (Fig. 5, Additional files 4 and 5: Tables S3 and S4). CcpA controls the preferential use of glucose over other sugars [21] and regulon analysis of the differentially expressed genes revealed that CcpA-regulated genes were indeed over-represented in the data set (Fig. 5b).

*L. lactis* contains three glucose import systems – two phosphotransferase systems, PTS^Man^ and PTS^Cel^, and a glucose facilitated diffusion system [22] – and expression of the genes encoding the high affinity PTS^Man^ were up-regulated in compared to the original strain, while those encoding the low affinity PTS^Cel^ were down-regulated (Fig. 5c). The effects of these gene expression changes were investigated further by monitoring the uptake of ^14^C-glucose in whole cells. In accordance with the sequencing and gene expression data, i.e. the glucose transporters themselves are not mutated but rather their expression is changed, the rate at which glucose was taken up by the cells was increased nearly 3-fold in the evolved strain, while the apparent affinity for glucose was unchanged (Fig. 5d). The ability to utilize other carbon sources was diminished for the evolved strains (Additional file 1: Figure S1). This coincided with the down regulation of, amongst others, genes encoding for maltose transporters, ABC sugar import systems and sugar utilization enzymes (Fig. 5a, Additional file 4: Table S3), many of which are CcpA-regulated (Fig. 5b).

In general, CcpA is a versatile global regulator that can acts as a transcriptional repressor or activator. An amino acid substitution in the DNA binding region may therefore have qualitatively different gene expression effects at different target promoters. This was indeed what we observed in the expression data set: 15 CcpA-regulated genes were down regulated, 6 up-regulated, while 11 remain unchanged. We next examined the molecular basis of these observations.

### Effect of M19I mutation on the binding affinity of CcpA

**Fig. 6:**
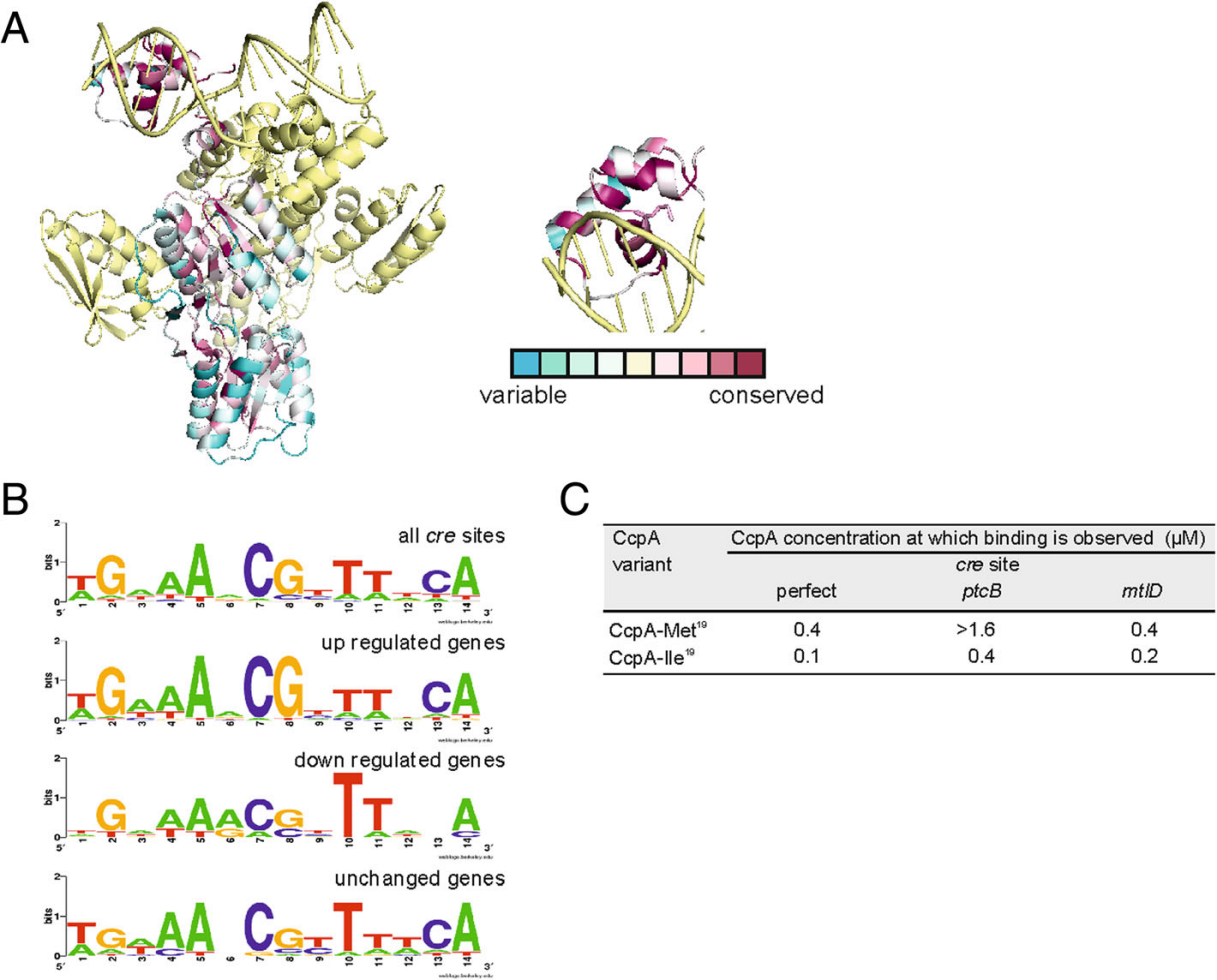
Mutations in *ccpA* result in increased binding affinity for certain *cre* sites. Mutations in the Met^19^ codon in the DNA binding domain of CcpA alter the binding affinity of CcpA for certain *cre* sites. **a** Using consurf db [43] and the *B. subtilis* CcpA-HPr complex bound to a synthetic cre site (3OQN) as a starting structure, the level of amino acid sequence conservation among LacI transcriptional regulators was analysed. One molecule of CcpA is colored according to the level of conservation within the LacI family of transcriptional regulators while the other molecule, HPr and the DNA are colored yellow. Dark purple indicates 100% conservation while blue indicates extensive sequence variation. The inset shows a view of the DNA-binding domain with the side chain of Met19 is shown. **b** Sequence logo diagrams representing the abundance and position of nucleotides in the CcpA-regulated genes of *L. lactis* MG1363. **c** Binding of CcpA to *cre* sites in vitro. The binding of CcpA-Met^19^ and CppA-Ile^19^ was tested with DNA sequences identified as *cre* sites upstream of *ptcB* and *mtlD* as well as a perfect *cre* site. As a negative control the CodY recognition site upstream of *oppD* was also tested (See also Additional file 1: Figure S6)

The effects of the *ccpA* mutations were investigated using homology modeling, molecular dynamic simulations and in vitro binding assays using purified CcpA from the mutated strains. The high-resolution structure of *B. subtilis* CcpA was used to model *L. lactis* CcpA (Fig. 6a) [23] since the one published for *L. lactis* CcpA lacks the N-terminal DNA-binding domain [24]. The structure from *B. subtilis* includes CcpA bound to the DNA catabolic response element (*cre* site) as well as the activating phosphoprotein HPr. Met19 is located in the second helix contained in the DNA binding domain. The methionine at this position is highly conserved across all Gram-positive CcpA proteins sequenced to date and in 4 natural isolates of *L. lactis* for which the *ccpA* locus was resequenced (Additional file 6: Table S6).

*L. lactis* CcpA binds to *cre* sites for which the consensus sequence is known: TGNNANCGNTTNCA. Sequence analysis revealed that putative *cre* sites upstream down-regulated genes were closest to the *cre* consensus sequence, while up-regulated genes deviated and were less likely to have both C7, G8 and C13 but always T10 (Fig. 6b).

The binding of CcpA from Genr0 and 445C1 to 4 DNA operators was examined: synthetic *cre* sites, *cre* site upstream of *ptcB*, cre site upstream of *mtlD* and, as a negative control, the CodY binding sequence upstream of *oppD* (Fig. 6c and Additional file 1: Figure S6), representing genes that are down regulated as well as up regulated in the evolved strains.

For all 4 *cre* sites tested in vitro, the CcpA-Ile19 variant showed an increase in binding affinity for the DNA operators selected (Fig. 6d and Additional file 1: Figure S6). This was most pronounced for the sequence upstream of *ptcB* and least pronounced for *mtlD*.

**Fig. 7:**
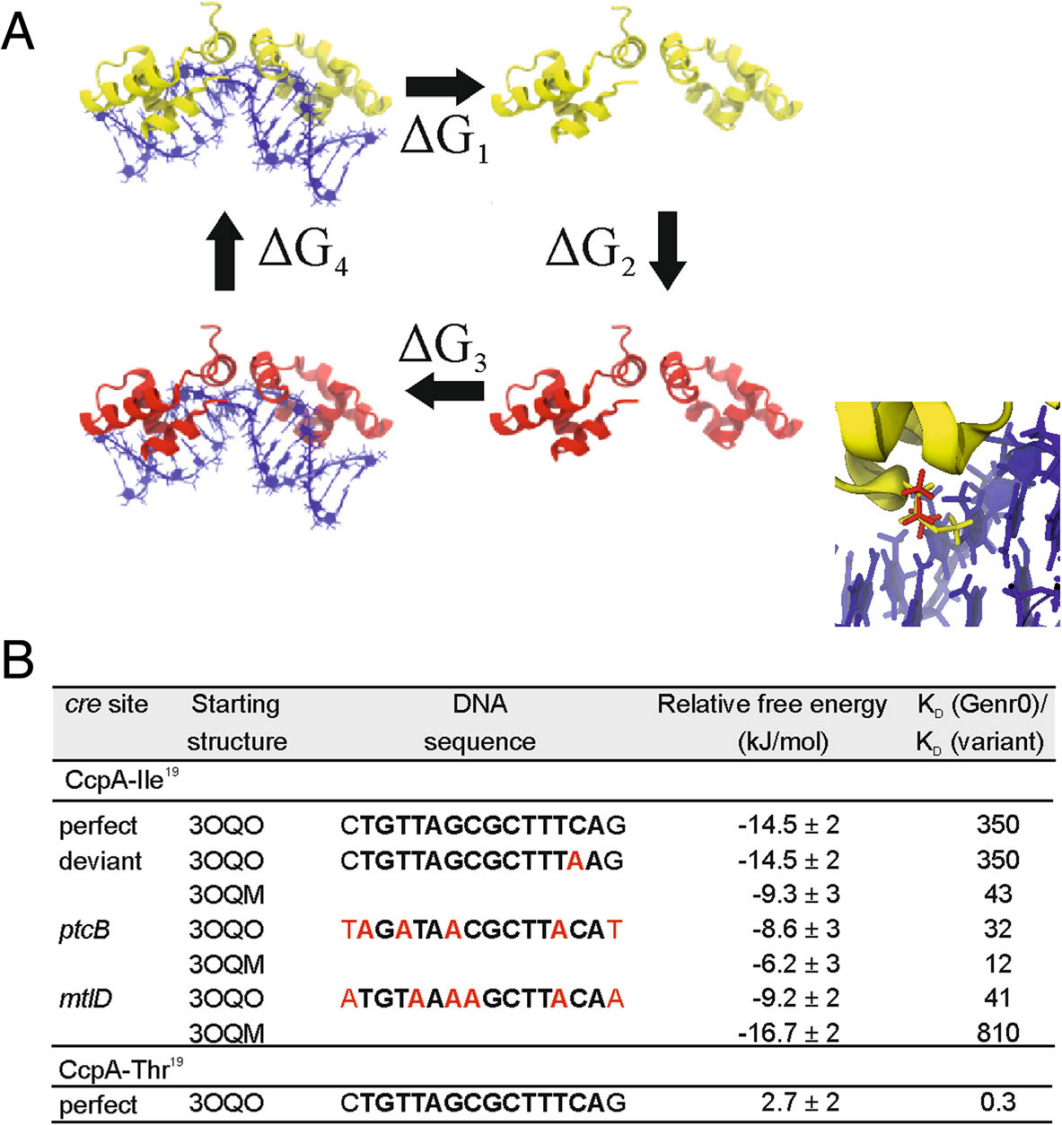
The thermodynamic cycle to calculate the relative binding free energies. The wild type protein is shown in yellow and the mutated protein in red (**a**). Note that while the color of the whole protein is different only a single residue is changed in both monomers of the mutated protein. The relative binding free energy is ΔG1 + ΔG3 which, based on the cycle, is equal to -(ΔG2 + ΔG4). The inset highlights the DNA binding domain of CcpA, with the methionine-19 position in red, and isoleucine at the same position in yellow. **b** Changes in relative binding free energy upon substitutions at Met19 in CcpA as calculated by molecular dynamics using two different starting structures i.e. 3OQO, with a synthetic *cre* site, and 3OQM, with the *cre* site from the *ackA2* promoter region

To complement the binding assays, molecular dynamic simulations were performed. This allowed us to determine the relative binding free energy of the CcpA variants to a similar set of DNA operators (Fig. 7). The effect of either the Ile or Thr substitution at position 19 was first investigated by calculating the relative binding free energies of CcpA to a canonical *cre* site [23]. The Ile variant had a more negative free energy of binding as compared to the wild type protein, whereas the Thr variant did not exhibit significantly different binding to the wild type protein. We then tested the effect of the DNA sequence on the binding free energy for wild type and evolved CcpA, using structures derived from CcpA bound to two different *cre* site sequences (3OQO, with a synthetic *cre* site, and 3OQM, with the *cre* site from the *ackA2* promoter region) [23]. When the Ile was present at position 19, tighter binding to all DNA sequences tested was computed, irrespective of the starting structure used.

### Population dynamics of prolonged cultivation experiments

**Fig. 8:**
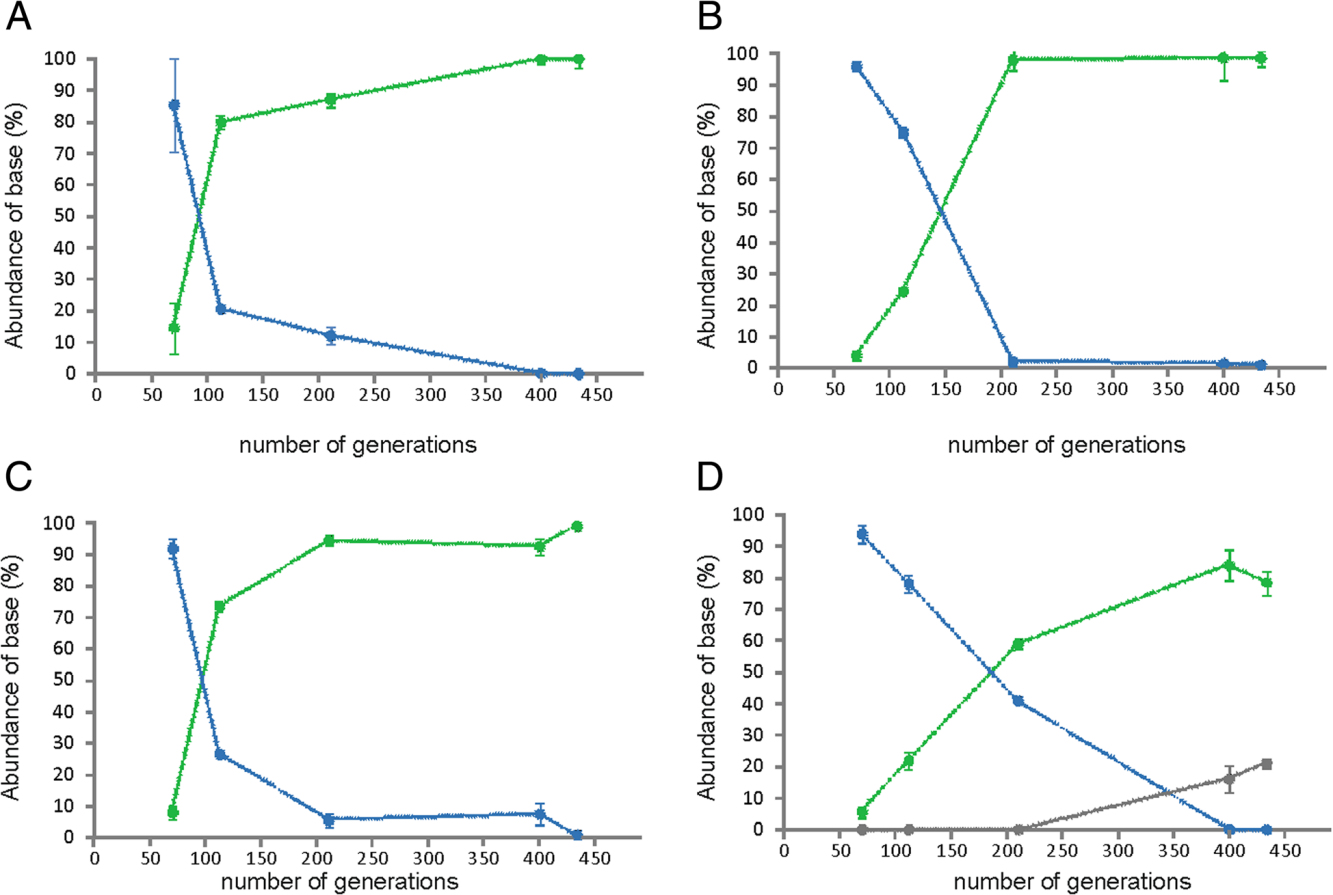
Abundance of the three CcpA species found during evolution experiment. *ccpA* accumulates the Met19 to Ile early in evolution experiment and quickly takes over the population. Indicated are the averages of two pyrosequencing reactions and in the case of strain 445C4, the abundance of the Thr mutation was verified by Sanger sequencing. The PCR was performed from the frozen stocks sampled during the continuous culture process. In green is indicated the prevalence of the Ile mutant, in blue wild type CcpA and in grey the Thr mutant. **a** 445C1, **b** 445C2, **c** 445C3, **d** 445C4

The frozen population stocks, collected periodically throughout the prolonged cultivations, served as snapshots of the adaptive evolution process to the chemostat environment. By determining the abundance of the CcpA-Ile19 variant in stocks collected at different generations, we could determine the wash-in dynamics that outcompeted the native CcpA-Met19. Quantification was carried out using pyrosequencing of the region containing the SNP in the *ccpA* locus. The CcpA-Ile19 variant emerged after 70 generations in the evolved populations and became dominant already by generation 210 (Fig. 8). While Met is encoded by ATG, ATA, ATC or ATT can encode Ile. Only the first two were identified in the D = 0.5 h^− 1^ evolution experiment, with the ATA mutant emerging prior to ATC (Additional file 1: Figure S7). At generation 445, when the experiment was stopped, over 97% of the population carried the CcpA-Ile19 variant.

We then tested whether the phenotypic changes imposed by the CcpA mutation would be sufficient to explain the wash-in kinetics observed using the glycerol stocks. For this purpose, a simple model of chemostat growth with two competing strains was used to simulate the prolonged experiment at a D of 0.5 h^− 1^ (Additional file 1: Table S7). The model could accurately fit the observed population dynamics, suggesting that the underlying mechanism can be fully understood in terms of chemostat wash-in kinetics and is attributable to the mutant CcpA regulator (Additional file 1: Figure S8). Sensitivity analysis of this model showed that two pairs of parameters cannot be estimated independently, only their ratios (Additional file 1: Figure S8D–E). These are (i) the Monod constants (K_M_) of the parent (Genr0) and the evolved (445C1-C4) strains, and (ii) the biomass yield of the parent strain and the emergence frequency of the fitter strain. The uncertainty should, however, not influence the conclusions derived from the model.

Another important aspect emerged from analyzing the model – the cells evolved at a D = 0.5 h^− 1^ were predicted to outcompete Genr0 at any constant dilution rate not higher than μ_max_. Since this can be experimentally verified, we performed new competition experiments.

### Parallel laboratory evolution and competition experiments under chemostat conditions at different dilution rates

We next addressed whether the CcpA-Ile19 variant was advantageous at the D of 0.5 h^− 1^ alone, or at different dilution rates as well. We performed new evolution experiments in the chemostat environment at a dilution rate of 0.62 h^− 1^. At a D closer to μ_max_ (~ 0,7 h^− 1^), the concentration of the limiting nutrient, in this case glucose, is higher [25], hence modulating the strength of the selection pressure applied. Experiments were carried out for as many as 1350 generations, in a similar fashion as was done for the D of 0.5 h^− 1^ (Additional file 1: Figure S9). Sanger sequencing of the *ccpA* locus from fragments, obtained by PCR using the frozen stocks as templates, indicated that indeed the CcpA-Ile19 substitution was washing in as predicted by the model. Re-sequencing of the whole-genome of single isolates of each of the populations evolved at 0.62 h^− 1^ revealed that once again the only mutation that was consistent across all isolates was in residue 19 of CcpA. This time all possible codons that encode Ile were found, suggesting that the amino acid substitution is the only factor that confers a strong fitness benefit.

**Fig. 9:**
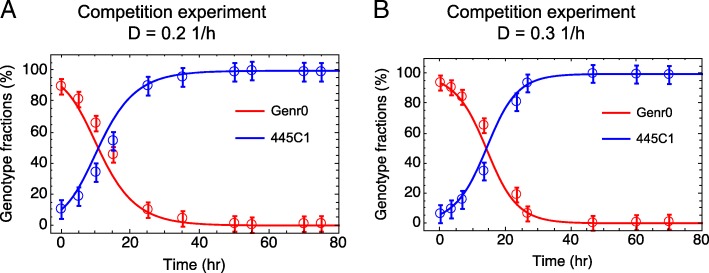
Chemostat competition experiments. Experiments were carried out between Genr0 (red) and 445C1 (blue) at a dilution rate of 0.2 h^− 1^ (**a**) and 0.3 h^− 1^ (**b**). Marks represent averages and standard deviations based on experimental data. Line depicts results from in silico simulations using the model presented here parameterized based on the population dynamics observed during the evolution experiments at a D = 0.5 h^− 1^ (Additional file 1: Figure S8)

We then tested whether a fitness advantage is also apparent at dilution rates far lower than μ_max_ by performing competition experiments in chemostats between Genr0 and 445C1 (harboring only the CcpA-Met19 mutation). We used our growth model parameterized using the data from the evolution experiments at a D of 0.5 h^− 1^, to simulate the population dynamics of competition experiments carried at a D of 0.2 and 0.3 h^− 1^ (Fig. 9). Based on this, we designed and performed experiments for dilution rates of 0.2 and 0.3 h^− 1^, in which a small fraction (~ 10%) of cells from a steady-state culture of 445C1 were mixed with a steady-state culture of Genr0 maintained at the same D. The population dynamics of these mixed cultures were monitored in time by collecting samples from the culture effluent, and determining relative fractions using pyrosequencing as before. The agreement between experimental data and model simulations (Fig. 9) reinforced that: (i) our simple growth model is able to capture the complexity of the evolutionary process; (ii) the parameters derived at a different D are sufficiently accurate to predict the phenotypic behavior of the evolved and parent strains; and (iii) the evolutionary process underlying the adaptation to a constant glucose-limiting environment is independent of the strength of the selection pressure.

## Discussion

In this paper, the relation between evolvability and evolutionary plasticity was studied by evolving *Lactococcus lactis* evolve under constant conditions in the chemostat. Growth on the preferred sugar, glucose, was studied. The type of selective pressure was kept constant, but its strength was varied by imposing different growth rates. Intriguingly, fixation of the same beneficial amino-acid substitution was found across all parallel evolutionary experiments, and for all growth rates tested. This demonstrates a high evolvability with low evolutionary plasticity for the adaptation process towards constant growth conditions under glucose-limitation i.e. one small change in the genotype was sufficient to initiate multiple phenotypic changes and strains which were able to outcompete the initial one.

We constructed miniature chemostats to allow a stable chemostat evolution study at fixed conditions. The working volume was decreased, which allowed experiments in quadruplicate for more than 1350 consecutive generations, generating true biological replicates, which allowed us to tackle issues related to the plasticity of microbial evolution. In glucose-limited chemostats, one expects enhanced selective pressure toward increased efficiency of glucose uptake (e.g. by elevating the expression of high-affinity- rather than of low affinity transport systems). However, this did not evolve as a main mechanism, but instead, a pleiotropic mutation in the global carbon metabolism regulator CcpA that, amongst other genes, increases the expression of genes for high affinity glucose PTS systems (e.g. PTS^Man^). Subsequently, the effect of the up-regulation of gene expression by an increased efficiency of uptake of glucose by the evolved strains was experimentally confirmed.

The molecular analysis of the fitness enhancing mutation in CcpA, suggests that the sequence of the DNA-binding domain determines an affinity for DNA that is advantageous in natural fluctuating environments (low-high glucose). The DNA binding domain of CcpA is highly conserved and in natural isolates of *L. lactis*, as well as in the CcpA proteins of other Gram-positive organisms such as *B. subtilis*, the Met residue is present at an equivalent position. Based on the high-resolution structures from *B. subtilis*, this residue directly interacts with the nucleobases in the structures containing *ackA2* and *glntR* promoter sequences but not with the synthetic *cre* site [23]. Whether CcpA binds with a higher or lower affinity to certain *cre* sites has been investigated in *B. subtilis* [26]. While the focus was on the *cre* site sequence itself, our study gives insights into the effect of the CcpA amino acid sequence on DNA binding. The direct-binding data show that the effect of the amino acid substitution consistently results in a tighter binding of CcpA to *cre* sequences, although not always to the same extent for each resulting mutation. Other factors such as distance of the *cre* site to the transcriptional start site or other transcriptional regulators governing the expression of the operon are likely to affect gene expression as well.

CcpA was not the only global regulator to accumulate mutations in our study, with the Phe substitution for Ile^214^ in the DNA binding domain of CodY. This mutation was only found in one chemostat but, like the mutation accumulated in *ccpA*, indicates a strategy of global gene expression modulation in response to the culture conditions tested.

In another study, adaptive evolution of *L. lactis* in emulsion – a method to select for high cell number – selected for a mutation in the glucose transporter itself, rather than in a regulator thereof [5]. In that case, the mutation reduced glucose transport activity, thereby mimicking a low-glucose environment associated with acetogenic metabolism, a high-yield strategy. Thus, the mutant relied on the native intracellular metabolic and gene-regulatory machinery for a rather drastic phenotypic change in metabolism.

In our study, at nutrient limitation, it appears that such an evolutionary path is not favored, but rather, the gene-regulatory circuit itself needs adjustment. Intriguingly, the same mutation occurred at two different growth rates, which require different rates of glucose uptake and glycolysis. This is only possible if the cells possess a form of non-genetic phenotypic plasticity that tolerates the adjustment of the gene-regulatory circuitry under different conditions. A recent functional genomics study of the parent Genr0 strain at different growth rates – using the same standard cultivation conditions – suggests that regulation at the metabolic level is the dominant form of phenotypic plasticity [25]. The presence of such non-genetic phenotypic plasticity seems therefore a requirement for our finding of low genetic plasticity and high evolvability.

In other relevant evolutionary studies on *Escherichia coli*, reproducible genetic mutations to growth under constant growth conditions were identified [27] as well as SNPs that, through rewiring of the transcriptional network, imparted a fitness advantage [28]. The fitness advantage was specific for growth under constant conditions and, as in our study, underpins a natural strategy.

In conclusion, our work shows that evolution to constant growth conditions can be mediated by global regulators such as the carbon catabolite protein CcpA. Metabolic engineering through the mutation of global regulators was first demonstrated by Stephanopoulos and coworkers [29]. We here show that the subtle manipulation of global regulators to change entire metabolic networks is a strategy already employed by nature. We find that for the evolution of *L. lactis* towards constant glucose-limitation, a high evolvability comes with low evolutionary plasticity.

## Material and methods

### Bacterial strains, media and growth conditions

*Lactococcus lactis* subsp. *cremoris* MG1363 was grown in a chemically defined medium developed for prolonged cultivation, CDMPC (Additional file 2: Table S1). The media consists of a phosphate buffer at pH 6.5, supplemented with all 20 amino acids, the vitamins biotin, DL-6,8-thioctic acid, D-pantothenic acid, nicotinic acid, pyridoxal hydrochloride, pyridoxine hydrochloride and thiamine hydrochloride, and trace metals (NH_4_)_6_Mo_7_O_24_, CaCl_2_, CoSO_4_, CuSO_4_, FeCl_2_, MgCl_2_, MnCl_2_ and ZnSO_4_. Glucose was added to a final concentration of 25 mM and cultivation was performed under an anaerobic headspace. In order to study the effects of long term cultivation under constant conditions, *L. lactis* MG1363 was cultivated in 4 chemostats run in parallel at 30 °C at the following dilution rates: 0.5 h^− 1^ for a total of 445 consecutive generation; 0.62 h^− 1^ for a total of 1350 consecutive generations. A small sample was removed every 15–25 generations and stored at − 80 °C in 20% (*v*/v) glycerol. In addition, the effluent was collected for optical density at 600 nm (OD_600_) measurements and HPLC analysis.

For subsequent studies, the glycerol stocks were used. 5 ml of fresh CDMPC was inoculated from the frozen glycerol stocks and grown for 16 h at 30 °C. The overnight culture was subsequently diluted to a starting OD_600_ of 0.025–0.050 in either CDMPC or M17 medium (Oxoid, Basingstoke, United Kingdom) supplemented with 0.5% (*w*/*v*) glucose or other carbon sources as indicated.

### Chemostat competition experiments between Genr0 and 445C1

The growth model parameterized using the data from the evolution experiments at a D of 0.5 h-1 was used to design the experiments so that the initial fraction and sampling time-scheme would be adequate to capture the predicted wash-in kinetics of the evolved cells. The competition experiments were preceded by running four-parallel chemostats for 10 volume changes as described above for Ds of 0.2 and 0.3 h-1. Of the four steady states independently established for each D, three included Genr0 and one 445C1. The competition stage was then initiated by transferring the 445C1 culture into the Genr0 steady states so that they made up ~ 10% of the population. A population sample was taken, after proper mixing, and considered to be the time zero (t0) sample. Subsequently, and according to a predetermined sampling scheme, samples were removed from the chemostat effluent. The relative fractions of the population corresponding to Genr0 and 445C1 were then determined in time, using pyrosequencing of the *ccpA* locus (detailed in the Additional file 1).

### Recombinant DNA methods

For cloning purposes, *Escherichia coli* XL1 blue was used. *ccpA* from the evolved *L. lactis* strains was cloned into the pQE30 plasmid essentially as described to create pQE30ccpA [30]. This allowed for the placement of 6 X His at the 5′-end of the *ccpA* gene. The *ccpA* gene was PCR amplified from genomic DNA from the single colony isolates from strains Genr0, 445C1, 445C2 and 445C4. *E. coli* XL1blue harbouring pQE30ccpA was used for overproduction of CcpA by IPTG induction. CcpA was purified using Ni-NTA resin (Qiagen, Germantown, MD, USA) as previously described [30]. The protein concentration was determined with the DC Protein assay kit (Bio-Rad, Hercules, CA, USA) using BSA as a standard.

### Whole-genome sequencing and mutation detection

DNA was extracted from the *L. lactis* single isolates using the GenElute™ Bacterial Genomic DNA kit (Sigma-Aldrich, St. Louis, MO, USA) according to the manufacturer’s instructions except that the cells were first pre-treated with lysozyme at 10 mg/ml for 1 h at 50 °C. The isolates were sequenced with 100 bp single-end reads across one lane of an Illumina HiSEQ 2000 flow cell (Illumina). The resulting reads were deposited in the NCBI-SRA database (PRJNA185994). Reads were mapped to the reference genome of *L. lactis* ssp. *cremoris* MG1363 (GenBank accession number AM406671) and mutations detected using the CLC BIO Genomic Work Bench Suit 4.5 (CLC BIO, Arhus, Denmark). The mutation lists were verified using PCR amplification and subsequent Sanger sequencing.

### Microarray analysis

For RNA extraction, all strains were grown in CDMPC to the mid-exponential growth phase. Total RNA was isolated using the High Pure RNA isolation Kit (Roche Applied Science) and the quality was tested using an Agilent Bioanalyzer 2100 (Agilent Technologies Netherlands BV). All procedure regarding microarrays were performed as described previously [31, 32]. Data was processed and normalized using the Lowess method with Micro-Prep [31] and lists of significant gene expression changes were generated on the basis of a Bayes *p-*vale and Benjamini Hochberg multiple testing corrections [33]. The DNA microarray data have been submitted to GEO with accession number GSE67657.

The expression data was further analyzed by the Rank Product analysis [34] using the software package R version 2.10.1. This generates a list of genes ranked according to ln ratio and calculates a conservative estimate of the percentage of false positives (pfp). Proteins with pfp values smaller than 0.05 (5%) were regarded as differentially expressed. The lists of significant gene expression levels were subjected to functional class analyses supported by the Genome2D webserver (http://www.molgenrug.nl/). This results in a list of classes that are overrepresented in the dataset supplied on the basis of a hypergeometrical distribution test. The data for the functional classes was derived from the KEGG public data base (http://www.genome.jp/kegg/ko.html) and for regulons from PePPER [35].

### [^14^C]-glucose uptake

Glucose uptake was monitored in whole cells using [^14^C]-labeled glucose essentially as previously described [36]. Briefly, all strains were grown in CDMPC and harvested at the mid-exponential growth phase, washed twice in ice-cold KPi buffer (50 mM, pH 6.5) and resuspended in KPi buffer to an OD_600_ of 0.5. Uptake assays were performed at 30 °C with stirring. [^14^C]-glucose was added to 100 μl cell suspension aliquots to a final concentration of 1.2–200 μM (0.005 μCi). The reactions were stopped by the addition of 2 ml of ice-cold 0.1 M LiCl and the samples were collected on 0.45 μm pore-size cellulose nitrate filters (Schleicher and Schuell GmbH, Dassel, Germany). The filters were again washed with 2 ml 0.1 M LiCl. The background was determined by adding the radiolabeled substrate to the cell suspension immediately after the addition of 2 ml of ice-cold LiCl, followed by filtering. The assays were performed in triplicate using cells from two independent cultures.

### Electrophoretic mobility shift assays

To determine the *cre* site DNA binding affinity of CcpA, binding assays were performed with Cy3-labeled oligonucleotides. DNA oligonucleotides were from Biolegio B.V. (Nijmegen, the Netherlands) with the (+) strand labelled at the 5’end with Cy3 and the (−) strand unlabeled. The oligonucleotides were mixed 1:1, incubated at 98 °C for 3 min and allowed to cool slowly to room temperature to facilitate annealing. The annealed DNA was used at 4 nM and added to binding buffer (20 mM Tris-HCl pH 8, 100 mM KCl, 10 mM MgCl_2_, 1 mM EDTA, 1 mM DTT, 5% glycerol) with additional BSA to 40 μg/ml. CcpA was added to concentrations between 100 nM and 800 nM. The binding reaction was performed at 30 °C for 20 min after which the samples were mixed with 5 X gel loading buffer (0.25% bromophenol blue, 40% sucrose). The samples were analysed by electrophoresis on 7.5% native polyacrylamide gels using a TBE buffer (90 mM Tris, 90 mM Boric Acid, 2 mM EDTA pH 8.0). The fluorescently-labelled oligonucleotides were visualised on a LAS-4000 imager (Fujifilm).

### Molecular dynamics simulations

The simulated structures were based on the crystal structures of CcpA bound to different DNA sequences by Schumacher et al. [23]. Homology models of *L. lactis* proteins were created using the Swiss Model Server [37]. The sequence identity of the DNA binding domain in proteins from *B. subtilis* and *L. lactis* is 77% and only this region of the protein was used for the simulation. Structures with differing DNA sequences were produced by fitting the differing bases to a crystal structure before equilibrating the system. The protein domains after residue 64 were removed from the simulation system and a harmonic bond with a 1000 kJ/(mol nm^2^) force constant was set between the backbone atoms of the last residues to restrain the structure of the remaining domains. Each system was solvated in a dodecahedron box where each face of the box was at least 1 nm from the protein and the DNA. This amounted in average to about 14,500 water molecules, 130 Na^+^ ions and 110 Cl^−^ ions, which corresponds to a 420 mM NaCl solution and counter-ions.

All simulations were run using the Gromacs 4.5.5 [38] simulation package. The protein and DNA were modelled with CHARMM27 force field with CMAP corrections [39, 40] together with the original TIP3P water model [41] as implemented in Gromacs 4.5.5.

Free energy changes were calculated from simulations using the Bennett acceptance ratio method [42] with the g_bar tool in Gromacs. Relative free energies of binding were calculated using a thermodynamic cycle described in Fig. 7.

### Pyrosequencing

Frozen glycerol stocks served as template material for PCR reactions. The PyroMark platform from QIAGEN was used for pyrosequencing and reactions were performed as per manufacturer’s instructions. The region surrounding the point mutations was amplified and biotinylated with the use of a biotinylated reverse primer. To detect the ratio of SNPs in the population, single stranded primers were designed to terminate before the codon containing the mutation.

### Miscellaneous

Oligopeptides were from JPT Peptide Technologies GmbH (Berlin, Germany). Primers used in this study were obtained from Macrogen (Amsterdam, the Netherlands). Sequences are given in Additional file 7.

## Additional files

Additional file 1: Additional materials and methods and supplementary figures 1–9. (ZIP 12575 kb)

Additional file 2: **Table S1.** Preparation of chemically defined medium for prolonged cultivations (CDMPC) for *Lactococcus lactis*. (DOCX 29 kb)

Additional file 3: **Table S2.** Single nucleotide polymorphisms (SNPs) detected in adapted strains. (DOCX 25 kb)

Additional file 4: **Table S3.** Genes/operons found to change significantly in at least three of the adapted strains compared to the original strain. Significant changes, indicated in bold, were considered for genes with a Bayes p-value score of less than 0.05 and a pfp value of less than 0.05. (DOCX 46 kb)

Additional file 5: **Table S4.** Changes in gene regulation in the adapted strains compared to the original strain. Significant changes, indicated in bold, were considered for genes with a Bayes p-value score of less than 0.05 and a pfp value of less than 0.05. (DOCX 115 kb)

Additional file 6: **Table S6.** Protein sequence alignment of CcpA from gram positive organisms for which the sequences are available in the public domain. (DOCX 157 kb)

Additional file 7: **Table S5.** Primers sequences used in this study. (DOCX 18 kb)
