# Supplementary material for: Adaption to glucose limitation is modulated by the pleotropic regulator CcpA, independent of selection pressure strength
Source: BMC Evol Biol. 2019 Jan 10;19:15. doi: 10.1186/s12862-018-1331-x (PMC6327505; doi:10.1186/s12862-018-1331-x)
Supplement: Supplementary file 1 — Additional materials and methods and supplementary figures 1–9. (ZIP 12575 kb) [file 12862_2018_1331_MOESM1_ESM.zip › 181104 suppl material.docx]

**Additional file 1**

***Title:*** Adaption to glucose limitation is modulated by the pleotropic regulator CcpA, independent of selection pressure strength

***Authors:***

Claire E. Price^a,b,c,1,2^, Filipe Branco dos Santos^c,d,e,2^, Anne Hesseling^a^, Jaakko J. Uusitalo^f^, Herwig Bachmann^c,d^, Vera Benavente^d^, Anisha Goel^c,d^, Jan Berkhout^d^, Frank J. Bruggeman^d^, Siewert-Jan Marrink^f^, Manolo Montalban-Lopez^a^, Anne de Jong^a^, Jan Kok^a^, Douwe Molenaar^c,d^, Bert Poolman^b^, Bas Teusink^c,d^, Oscar P. Kuipers^a,c^

***Author affiliations:***

^a^ Molecular Genetics group, University of Groningen, Nijenborgh 7, 9747 AG Groningen, the Netherlands

^b^ Department of Biochemistry, University of Groningen, Nijenborgh 4, 9747 AG Groningen, the Netherlands

^c^ Kluyver Center for Genomics of Industrial Fermentations/NCSB, Julianalaan 67, 2628 BC Delft, The Netherlands

^d^ Systems Bioinformatics, Faculty of Earth and Life Sciences, VU University Amsterdam, De Boelelaan 1085, 1081 HV Amsterdam, The Netherlands

^e^ Molecular Microbial Physiology Group, Faculty of Life Sciences, Swammerdam Institute of Life Sciences, University of Amsterdam, Science Park 904, Amsterdam 1098 XH, Netherlands

^f^ Molecular Dynamics group, University of Groningen, Nijenborgh 7, 9747 AG Groningen, the Netherlands

***Author footnotes:***

^1^ Current address: DSM Biotechnology Centre, Alexander Fleminglaan 1, 2613 AX, Delft, the Netherlands

^2^ Both authors contributed equally

^3^ Current address: Chr. Hansen, Boege Allé 10-12, 2970 Hoersholm, Denmark

***Corresponding authors:*** [b.teusink@vu.nl](mailto:b.teusink@vu.nl) and [o.p.kuipers@rug.nl](mailto:o.p.kuipers@rug.nl)

***Keywords:*** evolution, systems biology, lactic acid bacteria

**Results**

***Mutations unique to strains 445C2, 445C3 and 445C4***

The effect of the mutations identified in *rpoC* in 445C2 and *greA* in 445C4 on transcriptional fidelity were investigated using *lacZ* constructs with a premature stop codon at position Glu^13^ (P. Gamba and J.W. Veening, unpublished data). However, β-galactosidase activity was similar in the evolved strains and the original strains Genr0 and *L. lactis* MG1363 (Figure S2), indicating that in the evolved strains, RNA polymerase and elongation factor function as they do in wild type strains.

Since proteins encoded by llmg_1597, a hypothetical protein possibly with RNA-binding function, and *llrF,* a two-component system regulator, are not well-characterized, no prediction as to change or function could be made for the mutations identified. In contrast, CodY is a well-studied transcriptional regulator of nitrogen metabolism and based on structural studies it can be predicted that the SNP identified results in a Phe substitution for Ile^214^ in the DNA binding domain [1]. The absence of a high-resolution structure of CodY bound to a DNA operator makes any further prediction of the effect on DNA binding difficult.

Several SNPs were predicted to lie in non-coding regions at positions that lie in or near putative promoter sequences, thus making predictions as to the effect of such mutations difficult. The SNP at position 490263 resulted in an altered ribosomal binding site (GGAGGA to GGAGAA) upstream of the gene for a Hu-like DNA binding protein, *hllA*. This could suggest that a modest reduction of the translation of this mRNA, encoding a putative DNA-binding protein, could take place.

***Global gene expression patterns of evolved strains***

The evolved strains were revived in CDMPC and grown until mid-exponential phase at which microarray analysis using in-house *L. lactis* MG1363 slides [2] was performed. Gene expression in the evolved strains was compared to that of Gen0. A total number of 377 genes exhibited changed expression in the evolved strains (Additional file 5: Table S4). Approximately a third of the changes were in at least 3 of the 4 strains. Altered gene expression was dominantly in genes involved in membrane transport, especially carbohydrate import (Figure 5A, Additional files 4 and 5: Tables S3 and S4). Regulon analysis of the differentially expressed genes revealed that CcpA-regulated genes were over-represented in the data set. For strain 445C4, those regulated by CodY were prevalent as well (Figure 5B, Additional files 4 and 5: Tables S3 and S4).

CcpA controls the preferential use of glucose over other sugars [3], including expression of sugar uptake systems such as those for glucose. Transcriptome analysis of all the evolve strains revealed similar patterns of expression and increased glucose uptake (Figure 5C and D).

The ability to utilize other carbon sources was diminished for the evolved strains (Figure S1) in accordance with the down regulation of, amongst others, genes encoding for maltose transporters, ABC sugar import systems and sugar utilization enzymes in the microarray data (Additional file 4: Table S3). Many of these genes are CcpA-regulated.

***Phenotypic characterization of evolved strains to identify effects of transcriptional regulation.***

The strains were not only adapted to the constant culture conditions through expression changes in carbohydrate uptake and utilization systems, but also through changes in the expression levels of genes involved in amino acid uptake and metabolism. Ordinarily, *L. lactis* MG1363 grows faster when amino acids are taken up as oligopeptides than when only free amino acids are available (Figure S3B). This is due to the efficient import of oligopeptides by the major oligopeptide transporter Opp (Detmers et al., 2000). Cultivation in free amino acids led to the down regulation of the entire *opp* operon (Figure S3A) and the cells were as a result impaired in growth on oligopeptide containing medium (Figure S3B and C). The difference in growth in oligopeptide-containing medium between the evolved strains and Genr0 is well correlated with the expression levels of the *opp* operon except in the case of 445C4. In this strain, the down regulation of the *opp* operon appears to be partially compensated for by the concomitant up regulation of the analogous *opp2* operon. This operon was previously thought to be cryptic in *L. lactis* MG1363 [5], but in our evolved strain is expressed and OppA2 was detected using mass spectrometry (data not shown).

Prolonged cultivation under an anaerobic atmosphere had a dramatic effect on the ability of the evolved strains to utilize the electron transport chain present in *L. lactis*. *L. lactis* is unable to synthesize heme *de novo,* but if heme is added to the growth medium, the bacterium can synthesize the cytochromes needed for the generation of a proton motive force (PMF) and subsequent ATP synthesis at the membrane-embedded ATP synthase [6]. This results in an increase in biomass [7] as observed for Genr0 (Figure S4). The evolved strains did not show any increase in biomass upon addition of heme to the medium and expression data showed the down regulation of several NADH oxidases/dehydrogenases (Additional file 4: Table S3) which are likely to be responsible for the inability of these strains to respire.

In addition the evolved strains displayed a well-characterized long-chain phenotype associated with a deficiency in the major autolysin AcmA (Figure S5) [8]. The expression of the *acmA* gene was down regulated in all the evolved strains (Additional file 4: Table S3) and in agreement with the down regulation of the *acmA* gene, the evolved cells were less prone to lysis in the stationary growth phase (Figure S5).

***Evolution of Ccp-Thr^19^ variant***

The appearance of and subsequent dominance of the *ccpA* mutations was mapped in time during the evolution experiment through PCR amplification of the *ccpA* locus from the frozen stocks. The PCR products were resequenced using traditional Sanger sequencing, with manual chromatogram inspection, as well as pyrosequencing which allows for the quantitation of SNPs in a population. In addition, frozen stocks were streaked onto CDMPC agar plates and the *ccpA* locus was PCR amplified and resequenced from single colonies. The CcpA-Ile^19^ variant emerged around 50 generations in the evolved strains and by generation 150 was the dominant population in the chemostat (Figure S7). The pattern of emergence was different for strain 445C4, with the emergence of a second variant, i.e. CcpA-Thr^19^. This variant was only detected after 200 generations and by 445 generations both CcpA variants existed in the population. Sequencing of numerous single colony isolates could not detect any wild type *ccpA* by 445 generations (data not shown).

Sequencing of single colony isolates from the C4 frozen stocks, revealed that the unique CcpA-Thr^19^ variant is linked to the CodY-Phe^214^. At 445 generations the only combinations identified were CcpA-Ile^19^ and CodY-Ile^214^ (i.e. wild type CodY) and CcpA-Thr^19^ and CodY-Phe^214^. Stocks resequenced from earlier in the evolution experiment also contained CcpA-Thr^19^, CodY-Ile^214^ pairs, indicating that the *codY* mutations occurred in the unique CcpA- Thr^19^ background.

***Model description and parameter estimation***

The differential equations describing the rates of the change in the limiting nutrient concentration (s) and the biomass concentrations of the wild type (w) and the mutant (m) are,

$$\frac{ds}{dt}\left( t \right)=D\left( s_{r}-s\left( t \right) \right)-\frac{\mu_{w}}{Y_{w}}w\left( t \right)-\frac{\mu_{m}}{Y_{m}}m\left( t \right)$$

$$\frac{dw}{dt}\left( t \right)=\left( \mu_{w}-D \right)w(t)$$

$$\frac{dm}{dt}\left( t \right)=\left( \mu_{m}-D \right)m(t)$$

(1)

with: $\mu_{w}=\mu_{w}^{max}\frac{s(t)}{K_{w}+s(t)}$ , $\mu_{m}=\mu_{m}^{max}\frac{s(t)}{K_{m}+s(t)}$ , D as the dilution rate in h^-1^, μ as the specific growth rate in h^-1^, Y is the biomass yield in gDW.mM^-1^, μ/Y as the specific nutrient uptake rate in mM.gDW^-1^.h^-1^, K as the Monod constant in mM, s_r_ as the vessel concentration of the limiting nutrient in mM, s as the reactor concentration of the limiting nutrient in mM, and w and m as the biomass abundances in gDW.L^-1^. The subscripts “w” and “m” indicate whether the parameter concerns the wild type or the mutant. Alternatively, we could have taken the OD to represent the biomass concentration, which we now take as gDW.L^-1^.

Considering the experimental data, it is more convenient to consider a dimensional time defined as $\tau=Dt$, which is what we call ‘volume changes’. With this definition we obtain,

$$\frac{ds}{d\tau}\left( \tau\right)=\left( s_{r}-s\left( \tau\right) \right)-\frac{\mu_{w}}{DY_{w}}w\left( \tau\right)-\frac{\mu_{m}}{{DY}_{m}}m\left( \tau\right)$$

$$\frac{dw}{d\tau}\left( \tau\right)=\left( \frac{\mu_{w}}{D}-1 \right)w(\tau)$$

$$\frac{dm}{d\tau}\left( \tau\right)=\left( \frac{\mu_{m}}{D}-1 \right)m(\tau)$$

(2)

Since we are considering the fixation of the mutant, the initial conditions are given by the steady state of wild type in the absence of the mutant:

$$w\left( 0 \right)= Y_{w}\frac{D\left( K_{w}+s_{r} \right)-s_{r}\mu_{w}^{max}}{D-\mu_{w}^{max}}$$

$$s\left( 0 \right)=\frac{DK_{w}}{\mu_{w}^{max}-D}$$

(3)

Equations 2 and 3 were fitted against the experimental data. The results of the fit are shown in Table S7 and Figure S8. These data indicate that observed experimental data is in agreement with a basic chemostat model of the competitive growth of two microorganisms. The values of the fitted parameters are not the only set of values that describe the data. The data set is too small, and hence more variables would have to be measured to obtain a unique estimation of parameter values.

Table S7: Overview of parameters (fitted and experimental settings).

| Parameter | Wild type (w) | Mutant (m) |
| --- | --- | --- |
| Yield on substrate, Y (g/mM) | 0.076 | 0.08 |
| Maximal growth, μ^max^ (hr^-1^) | 0.71 | 0.59 |
| Monod constant, K (mM) | 0.019 | 0.0033 |
|  | Experiment settings | |
| Dilution rate, D (hr^-1^) | 0.5 | |
| Reservoir substrate concentration, Sr (mM) | 25 | |

***Supplementary materials and methods***

**CDMPC**

The chemically defined medium we developed for prolonged cultivation (CDMPC) was based on the nutrient requirements and biomass composition of *L. lactis* MG1363. The major differences compared to previously published chemically defined media for *L. lactis* [9–11] are (i) the removal of acetate and ammonium ions; (ii) removal of redundant and/or unessential vitamins such as vitamin B_12_, or those that could in addition impose (downstream) technical challenges such as riboflavin, which fluoresces, and folate, which is difficult to dissolve and likely to precipitate during media preparation; (iii) removal of trace elements that are likely to precipitate; (iv) removal of all non-essential nucleic acid precursors; (v) adjustment of phosphate concentration such that it provides enough buffering capacity to enable growth until OD_600_ of 0.8, while not inhibiting growth rate; (vi) adjustment of amino acid composition to 2.5-fold the requirement for 1 gDW∙L^-1^ according to the biomass composition [12]; and (vii) implementation of a new protocol that reduces variations between media batches by avoiding precipitation and heat or light degradation.

**Standardized cryopreservation and inoculation procedure**

The outcome of laboratory evolution experiments can be greatly affected by the composition of the population used to seed each of the prolonged cultivations. To minimize background genetic variation and variations in pre-culture condition, we developed a standardized cryopreservation and inoculation procedure inspired by industrial procedures to create, maintain and distribute single strain isolate stocks (Figure 1, main text). All the prolonged cultivations in this study were carried out from the working stocks generated via this procedure.

*Single colony isolation and parent-stock preparation*

A glycerol stock of *L. lactis* MG1363 (provided by Jan Kok, University of Groningen, the Netherlands) was used to inoculate M17 enriched with 0.5% glucose (GM17). This culture was streaked out from GM17 onto CDMPC agar plates (CDMPCA) and incubated for 48 hours at 30º C. An isolated colony was transferred to CDMPC and incubated for 24 hours at 30º C. A 1% inoculum of the freshly grown culture was used to inoculate CDMPC. After 5 consecutive transfers under the same conditions and using 1% inoculum to seed subsequent cultures, the resulting culture was streaked out in CDMPCA and incubated for 24 hours at 30º C. An isolated colony was transferred to 7.5 ml of CDMPC and incubated for 24 hours at 30º C reaching a final OD_600_ of 1.165. “Parent-stocks” were then prepared by adding 3.75 ml of sterile 60% glycerol and dividing the mixture in 11 aliquots of 1 ml in self-standing Nunc™ Cryotubes (366656), kept at -80º C for at least for 48 hours before reviving or moving.

*Working-stock preparation*

One of the 11 aliquots of the parent-stock was used to inoculate 199 ml of CDMPC (0.5% inoculum) and incubated for 20 hours at 30º C, until the culture was full-grown. 100 ml of sterile 60% glycerol was then added, resulting in 300 ml of 20% glycerol stock of *L. lactis* G0 in CDMPC. “Working-stocks” were then prepared by dividing the latter in 960 aliquots of 0.3 ml in 1.2 ml sterile polypropylene cluster tubes 96-well racked from Corning Incorporated Costar® (4413). These were capped using 8-Cap Strips from Corning Incorporated Costar® (4418) and separated into individual vials using a slightly heated bistoury and ruler. Working-stocks were stored at – 80º C for at least 24 hours before reviving or moving.

*Standardized inoculation procedure*

A working-stock was revived by thawing a 0.3 ml aliquot of 20% glycerol stock of *L. lactis* G0 in CDMPC and used to inoculate 44.7 ml of CDMPC (0.67% inoculum). This culture was incubated for 16 hours at 30º C, which corresponds to the time at which the culture reaches early stationary phase (OD_600_ ~1.00±0.15). This freshly-grown culture cultivated in a standardized fashion and seeded from a controlled inoculum, was then used directly as the inoculum of downstream cultivations and assays (chemostats, batch cultures, etc.) performed by different experimentalists across several laboratories committed to working in a standardized way.

*Moving and storage of cryopreserved stocks*

The 11 aliquots of 20% glycerol stock of *L. lactis* G0 in CDMPC (parent-stock) were divided amongst the laboratories of the University of Amsterdam, VU University Amsterdam, Groningen University and Research Center and Wageningen University, preserved in different -80º C freezers within these institutes. The 960 aliquots of 20% glycerol stock of *L. lactis* G0 in CDMPC (working-stock) were divided amongst all the collaborators involved in standardized systems biology studies using *L. lactis* G0 as the model organism. Both the parent- and working-stocks were kept frozen at all times and transported accordingly.

**Experimental set up**

Our design made use of bioreactor lids that can fit any vessel with a GL45 thread. It allowed for two connectors to be inserted at variable depths, used here to control the working volume and sampling. The custom-made bioreactors were set-up to make four parallel chemostat cultivations controlling stirring, temperature, pH, flow, volume and gas composition. The newly developed caps are versatile and can be readily adapted to other types of controlled cultivation, such as fed-batch, pH-auxostats, turbidostats, retentostats, amongst others. Culture samples were withdrawn directly through a port present in the centre of the cultivation vessel or by rerouting the effluent using acetal quick-disconnect couplings (Masterflex via Cole Parmer, Schiedam, Netherlands) Throughout the prolonged cultivations samples were collected from the effluent to leave the cultivation undisturbed.

For each set of parallel chemostats, the temperature was controlled using a water bath (Thermo Haake DC30-W13/B via Cole Parmer, Schiedam, Netherlands) set to 30ºC. Homogenization was ensured using an immersed stirrer unit connected to a remote controller (Cole Parmer, Schiedam, Netherlands) set to ~300 rpm. Mixing was tested by pulsing with glucose to a final concentration of 60 mM and sampling at three different positions of the bioreactor. Minor differences were observed between samples harvested at different time points (<5%), and at small-time scales (<10 seconds) the solution appeared equilibrated. The pH was measured separately in each culture using a 120 mm gel-filled pH-sensor (Applikon, Schiedam, Netherlands) connected to an Alpha-pH800 (Eutech, Thermoscientific) that controlled a 1 rpm L/S pump drive equipped with an Easy-Load II pump head (Masterflex via Cole Parmer, Schiedam, Netherlands) that titrated the culture with a solution of 2.5 M NaOH. The chemostat was continuously flushed through the headspace with a mixture of 5% CO2 and 95% N2 at a flow rate of 10 culture volumes per hour (i.e. 600 ml/h). The dilution rates of the different prolonged cultivations carried out in this study were set by controlling the flow of CDMPC using a variable speed L/S pump drive equipped with four Easy-Load II pump heads (Masterflex via Cole Parmer, Schiedam, Netherlands). All parallel cultivations were supplied with CDMPC from the same medium vessel.

**Molecular dynamics simulations – free energy calculations**

The free energy of mutating the protein was calculated while protein was bound to DNA and free in solvent and the relative binding free energy of the mutated protein compared to the wild type was obtained by subtracting the free energy of the former from the latter (Figure 7).

The free energy of mutating the protein was calculated using a topology with two side chains at residue 19. The two side chains did not interact with each other and the mutation was done in three steps. In the first step, the charges of the wild type methionine side chain were uncoupled from rest of the system while the mutated side chain was completely non-interacting. In the second step, the Lennard-Jones interactions of methionine side chain were uncoupled while at the same time the Lennard-Jones interactions of the mutated side chain were coupled to the side chain. In the third step, the charges of the mutated side chain were coupled to the system while the methionine side chain was completely non-interacting. The first and third steps were performed using 5 simulations with different lambda values while the second step used 11 lambda values. Soft-core interactions were used in the second step with soft-core parameter **α** set to 0.5. An error estimate is calculated from block averaging each 1 ns of the simulations and using the standard deviation of the obtained values as the error.

In the simulations, the protein monomers can move relatively freely in solution when DNA is not present and sampling of conformations becomes an issue. To ensure that the conformations sampled would be similar for all CcpA variants, rotational and translational constraints were used to prevent the monomers from moving with reference to each other as described in (50).

A 2 fs time step was used with all bonds constrained using the LINCS algorithm [14]. Simulations were run with periodic boundary conditions in all directions. Non-bonded interactions were treated as recommended in [15]. Electrostatics were calculated using PME [16] with a 1.2 nm cut-off. Lennard-Jones interactions were switched off between 1.0 and 1.2 nm and dispersion correction was applied to both energy and pressure. The protein-DNA system and the solvent were separately coupled to a 298 K heat bath using the velocity rescaling thermostat [17] with τ_T_ = 0.5 ps. A 1 bar pressure was kept using an isotropic Parrinello-Rahman [18] pressure coupling with τ_p_ = 4.0 ps and 4.5e-5 bar^-1^ compressibility. Each system was energy minimized and briefly equilibrated with position restraints before being equilibrated for 10 ns. After equilibration, each simulation was run for 20 ns except the systems with deviant *cre* sites that were simulated for 10 ns.

**Enzyme reactions**

L-lactate dehydrogenase (L-LDH) was purchased from Sigma-Aldrich (St. Louis, MO, USA). L-LDH was added to culture supernatant to a final concentration of 0.2U/ml, NADH was added to 2 mM and the reaction incubated for 15 minutes at 25°C. X-prolyl dipeptidyl aminopeptidase (PepX) activity was measured using the chromogenic substrate Ala-Pro-*p-*nitroanilid (Bachem Feinchemicalien AG, Bubendorf, Switzerland) as described [19].

β-galactosidase assays were performed as previously described [20, 21].

**Isolation of proteins from the culture supernatant**

All strains were grown in CDMPC at 30°C, without pH control or stirring, to an OD_600_ of 1.0 which corresponds to late-exponential growth phase. Cells were harvested by centrifugation and the supernatant was filtered through a 0.22 μm filter. Proteins were precipitated by the addition of TCA to a final concentration of 6 %. The resulting pellet was washed with acetone and resuspended in SDS-PAGE loading buffer.

**Protein identification by liquid chromatography-mass spectrometry**

The samples were separated by SDS-PAGE and stained with Bio-Safe Coomassie (Bio-Rad, Hercules, CA, USA). In-gel tryptic digestion was followed by peptide extraction and LC-MS/MS as described in detail in Drop *et al.,* 2011 The MS raw data was submitted to Mascot (Version 2.1, Matrix Science, London, UK) and searched against the *L. lactis* MG1363 proteome. Protein identifications were based on at least 2 unique peptides identified by MS/MS, each with a confidence of identification probability of at least 95%. Identifications were based on at least 2 unique peptides identified by MS/MS, each with a confidence of identification probability of at least 95%.

**Western blotting**

SDS-PAGE and immunoblot analyses were carried out according to established methods [22]. Signal capture and quantification were performed using the FUJIFILM LAS-4000 luminescent image analyzer. Antisera against OppA were from our own laboratory collection. Alkaline phosphatase conjugated anti-rabbit was purchased from Sigma-Aldrich.

**Supplementary figure legends**

**Figure S1.** (A) Rate of utilization of carbon sources present in biology plates. Cells were grown in CDMPC until mid-exponential phase, washed in CDMPC without glucose and resuspended in CDMPC without glucose containing 5 µg/ml chloramphenicol to an OD600 of 2. 150 µl of the culture suspension was added to each of the wells of the biology plate and acid formation was monitored at OD485 for 4 hours. The maximum rates of utilization were calculated and the averages of two separate experiments are shown. Only substrates used by at least one strains are shown. (B) Substrates not utilized under the conditions tested.

**Figure S2**. Transcriptional fidelity in evolved strains is comparable to the original ones. Strains were grown in CDMPC and harvested at the mid-exponential phase of growth. Data are averages of three measurements with the error bars indicating the standard deviation. Specific β-galactosidase activity was measured in the strains indicated using pPG6 (P. Gamba and J.W. Veening, unpublished data) which contains a premature stop codon at Glu13.

**Figure S3.** Growth of the evolved strains is inhibited but not abolished when leucine is supplied as a nonapeptide. (A) Differentially expressed genes involved in oligopeptide uptake in *L. lactis* MG1363. Significant changes were considered for genes with a Bayes p-value score of less than 0.05 and a pfp value of less than 0.05 and are highlighted in green for upregulated genes and in red for down regulated genes. Gene expression changes not meeting the significance cut off values are highlighted in grey. (B) Strains were grown in CDMPC or in CDMPC without leucine but with a leucine-containing nonapeptide. Growth was followed for 16 hours. Shown are Genr0 (black lines) and 445C4 (grey lines) grown in CDMPC (solid lines) or CDMPC with leucine-containing peptide (dashed lines). (C) The difference in the final OD600 values reached after growth for 16 hours are shown for all strains. Shown are the averages between three separate experiments with the standard deviation indicated.

**Figure S4.** Evolved cells are no longer able to respire when heme is added to the medium. (A) No biomass increase is observed in the evolved strains when heme is added to the growth medium. Strains were grown in GM17 medium for 16 hours under aerobic conditions with and without heme which was added to 2 µg/ml. Shown are the average values from three separate experiments with the standard deviation indicated. (B) Differentially expressed genes involved in aerobic respiration in *L. lactis* MG1363. Significant changes were considered for genes with a Bayes p-value score of less than 0.05 and a pfp value of less than 0.05 and are highlighted in red for down regulated genes. Gene expression changes not meeting the significance cut off values are highlighted in grey.

**Figure S5.** Evolved strains are less prone to lysis on prolonged cultivation than the original strain. (A) Expression changes for acmA are shown. Differentially expressed genes involved in aerobic respiration in L. lactis MG1363. Significant changes were considered for genes with a Bayes p-value score of less than 0.05 and a pfp value of less than 0.05 and are highlighted in red for down regulated genes. (B) PepX activity was measured in the culture supernatant at the time points indicated. Activity was measured using the chromogenic substrate Ala-Pro-p-nitroanilid and the color change was monitored at OD405. One representative experiment is shown. (C) Evolved strains sediment during growth. Overnight cultures of Genr0 and 445C1 in CDMPC are shown. (D) Sedimentation is caused by the formation of long cell chains. Typical pictures are shown for Genr0 and 445C1 grown for 16 hours in CDMPC. The cells were visualized using a Zeiss light microscope and a Zeiss digital camera. Magnification, ×1 000.

**Figure S6.** Binding of CcpA to cre sites *in vitro*. The binding of CcpA-Met19 and CppA-Ile19 was tested with DNA sequences identified as cre sites upstream of *ptcB* and *mtlD* as well as a perfect cre site referred to as syn. As a control, the CodY recognition site upstream of *oppD* was also tested.

**Figure S7.** Abundance of nucleotides at third position in the Met19 codon. Indicated are the averages of two pyrosequencing reactions. The PCR was performed from the frozen stocks sampled during the continuous culture process. In green is indicated the prevalence of adenosine, in red thymine, in blue cytosine and in black guanine. The average standard deviation between the separate reactions was 4.4. (A) 445C1, (B) 445C2, (C) 445C3, (D) 445C4.

**Figure S8.** Overview of the fitting results. (A and B) The fitted model (eq. 2 and 3) and the experimental data. (C) Predicted dynamics of the concentration of the limiting nutrient in the chemostat. The mutant wins the competition with the wild type because it can grow at a specific growth rate equal to D at a lower limiting nutrient concentration than the wild type. (D) Influence of parameter values on the objective, indicating that all the parameter values reach their optimal values (reported in Table S7) when the fit objective reaches its minimal value. (E) Illustration that the Monod constant of the wild type and the mutant cannot be independently estimated; i.e. the same objective value is obtained for different values of those parameters. The plot suggests that the ratio of Kw/Km can be fitted but not their individual values.

**Figure S9.** **Strain characteristics during the evolution experiments at D of 0.62h^-1^ and of the resulting evolved strains.** For each of the chemostats (C1, open triangles; C2, open circles; C3, closed triangles; and C4, closed circles) the biomass concentration (A) along with organic acids (C) were measured. The values for lactate (orange), formate (blue), acetate (green) and ethanol (red) are shown. The levels of pyruvate increased in all the chemostats during the evolution experiment (B). Mean concentrations at each generation are shown by data symbol ‘x’ and a 3^rd^ order polynomial fit solely used for visualizing the overall trend.

**References**

1. Levdikov VM, Blagova E, Joseph P, Sonenshein AL, Wilkinson AJ. The Structure of CodY, a GTP- and Isoleucine-responsive Regulator of Stationary Phase and Virulence in Gram-positive Bacteria. J Biol Chem. 2006;281:11366–73.

2. Kuipers OP, de Jong A, Baerends RJS, van Hijum SAFT, Zomer AL, Karsens HA, et al. Transcriptome analysis and related databases of Lactococcus lactis. Antonie Van Leeuwenhoek. 2002;82:113–22.

3. Deutscher J, Francke C, Postma PW. How phosphotransferase system-related protein phosphorylation regulates carbohydrate metabolism in bacteria. Microbiol Mol Biol Rev. 2006;70:939–1031.

4. Tynkkynen S, Buist G, Kunji E, Kok J, Poolman B, Venema G, et al. Genetic and biochemical characterization of the oligopeptide transport system of Lactococcus lactis. J Bacteriol. 1993;175:7523–32.

5. Sanz Y, Lanfermeijer FC, Hellendoorn M, Kok J, Konings WN, Poolman B. Two homologous oligopeptide binding protein genes (oppA) in Lactococcus lactis opp2 [corrected]. Int J Food Microbiol. 2004;97:9–15.

6. Brooijmans RJW, Poolman B, Schuurman-Wolters GK, de Vos WM, Hugenholtz J. Generation of a membrane potential by Lactococcus lactis through aerobic electron transport. J Bacteriol. 2007;189:5203–9.

7. Cesselin D, Lamberet G, Duwat P, Sourice S, Vido K, Gaudu P, et al. Respiration Capacity of the Fermenting Bacterium Lactococcus lactis and Its Positive Effects on Growth and Survival. J Bacteriol. 2001;183:4509–16.

8. Buist G, Kok J, Eenhouts K, Dabrowska M, Venema G, Haandrikman AJ. Molecular Cloning and Nucleotide Sequence of the Gene Encoding the Major Peptidoglycan Hydrolase of Lactococcus lactis, a Muramidase Needed for Cell Separation. J Bacteriol. 1995;177:1554–63.

9. Thomas TD, Ellwood DC, Longyear VM. Change from homo- to heterolactic fermentation by Streptococcus lactis resulting from glucose limitation in anaerobic chemostat cultures. J Bacteriol. 1979;138:109–17.

10. Poolman B, Konings WN. Relation of growth of Streptococcus lactis and Streptococcus cremoris to amino acid transport. J Bacteriol. 1988;170:700–7.

11. Jensen PR, Hammer K. Minimal Requirements for Exponential Growth of Lactococcus lactis. Appl Environ Microbiol. 1993;59:4363–6.

12. Oliveira A, Nielsen J, Förster J. Modeling Lactococcus lactis using a genome-scale flux model. BMC Microbiol. 2005;15:1–15.

13. van Dijk M, Wassenaar TA, Bonvin AMJJ. A Flexible, Grid-Enabled Web Portal for GROMACS Molecular Dynamics Simulations. J Chem Theory Comput. 2012;8:3463–72.

14. Hess B, Bekker H, Berendsen HJC, Fraaije JGEM. LINCS: A Linear Constraint Solver for Molecular Simulations. J Comput Chem. 1997;18:1463–72.

15. Bjelkmar P, Larsson P, Cuendet MA, Hess B, Lindahl E. Implementation of the CHARMM Force Field in GROMACS: Analysis of Protein Stability Effects from Correction Maps, Virtual Interaction Sites, and Water Models. J Chem Theory Comput. 2010;6:459–66.

16. Essmann U, Perera L, Berkowitz ML, Darden T, Lee H, Pedersen LG. A smooth particle mesh Ewald method. J Chem Phys. 1995;103:8577–93.

17. Bussi G, Donadio D, Parrinello M. Canonical sampling through velocity rescaling. J Chem Phys. 2007;126:14101.

18. Parrinello M, Rahman A. Polymorphic transitions in single crystals: A new molecular dynamics method. J Appl Phys. 1981;52:7182–90.

19. Buist G, Venema G, Kok J. Autolysis of Lactococcus lactis Is Influenced by Proteolysis. J Bacteriol . 1998;180:5947–53.

20. Kloosterman TG, Hendriksen WT, Bijlsma JJE, Bootsma HJ, van Hijum SAFT, Kok J, et al. Regulation of Glutamine and Glutamate Metabolism by GlnR and GlnA in Streptococcus pneumoniae. J Biol Chem . 2006;281:25097–109.

21. Israelsen H, Madsen SM, Vrang A, Hansen EB, Johansen E. Cloning and partial characterization of regulated promoters from Lactococcus lactis Tn917-lacZ integrants with the new promoter probe vector, pAK80. Appl Environ Microbiol . 1995;61:2540–7.

22. Towbin H, Staehelin T, Gordon J. Electrophoretic transfer of proteins from polyacrylamide gels to nitrocellulose sheets: procedure and some applications . Proc Natl Acad Sci . 1979;76:4350–4.
