# Supplementary figures and images for: Adaption to glucose limitation is modulated by the pleotropic regulator CcpA, independent of selection pressure strength
Source: BMC Evol Biol. 2019 Jan 10;19:15. doi: 10.1186/s12862-018-1331-x (PMC6327505; doi:10.1186/s12862-018-1331-x)

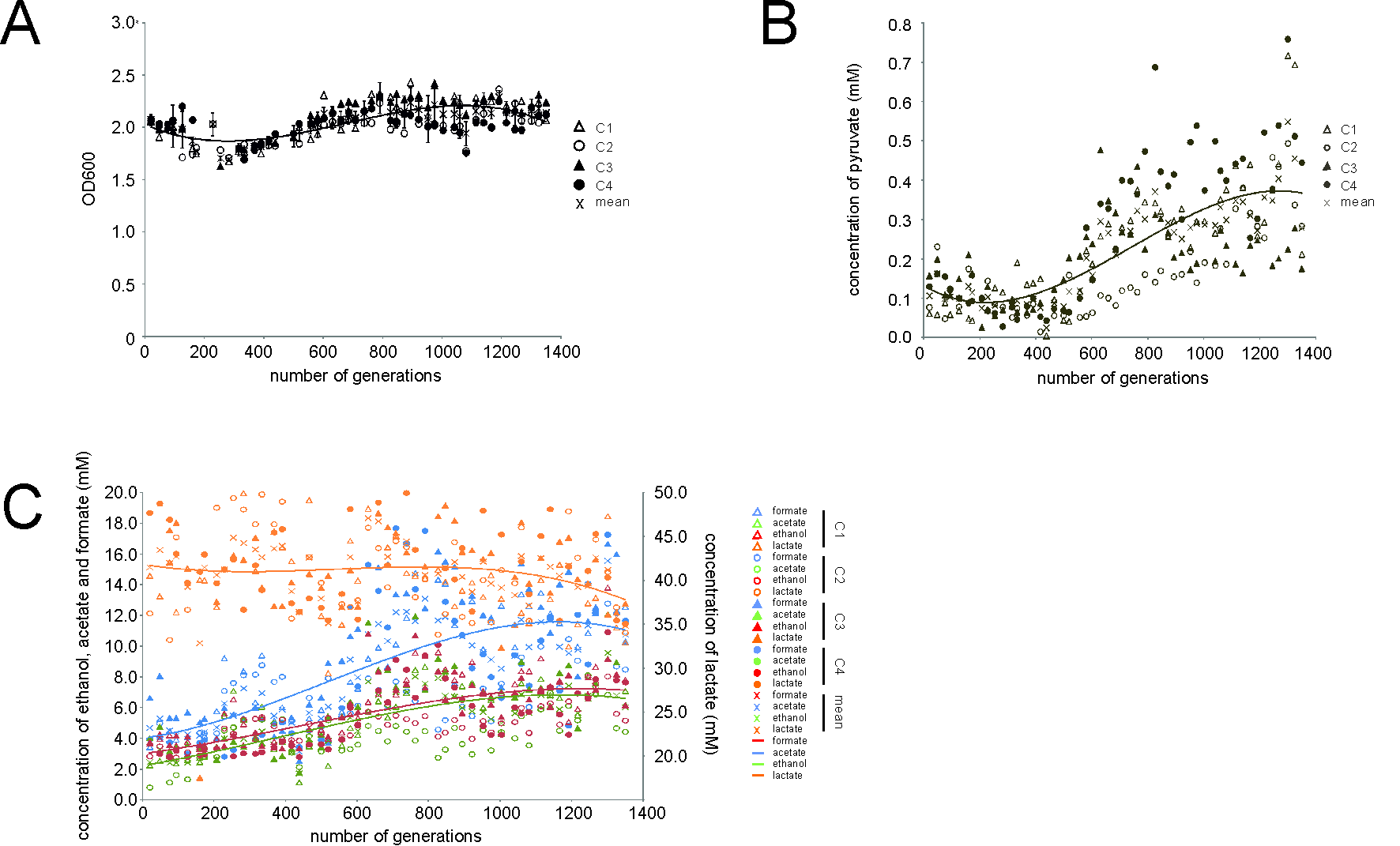

Supplement: Supplementary file 1 — Additional materials and methods and supplementary figures 1–9. (ZIP 12575 kb) [file 12862_2018_1331_MOESM1_ESM.zip › BMC Suppl Figure 9.tif]

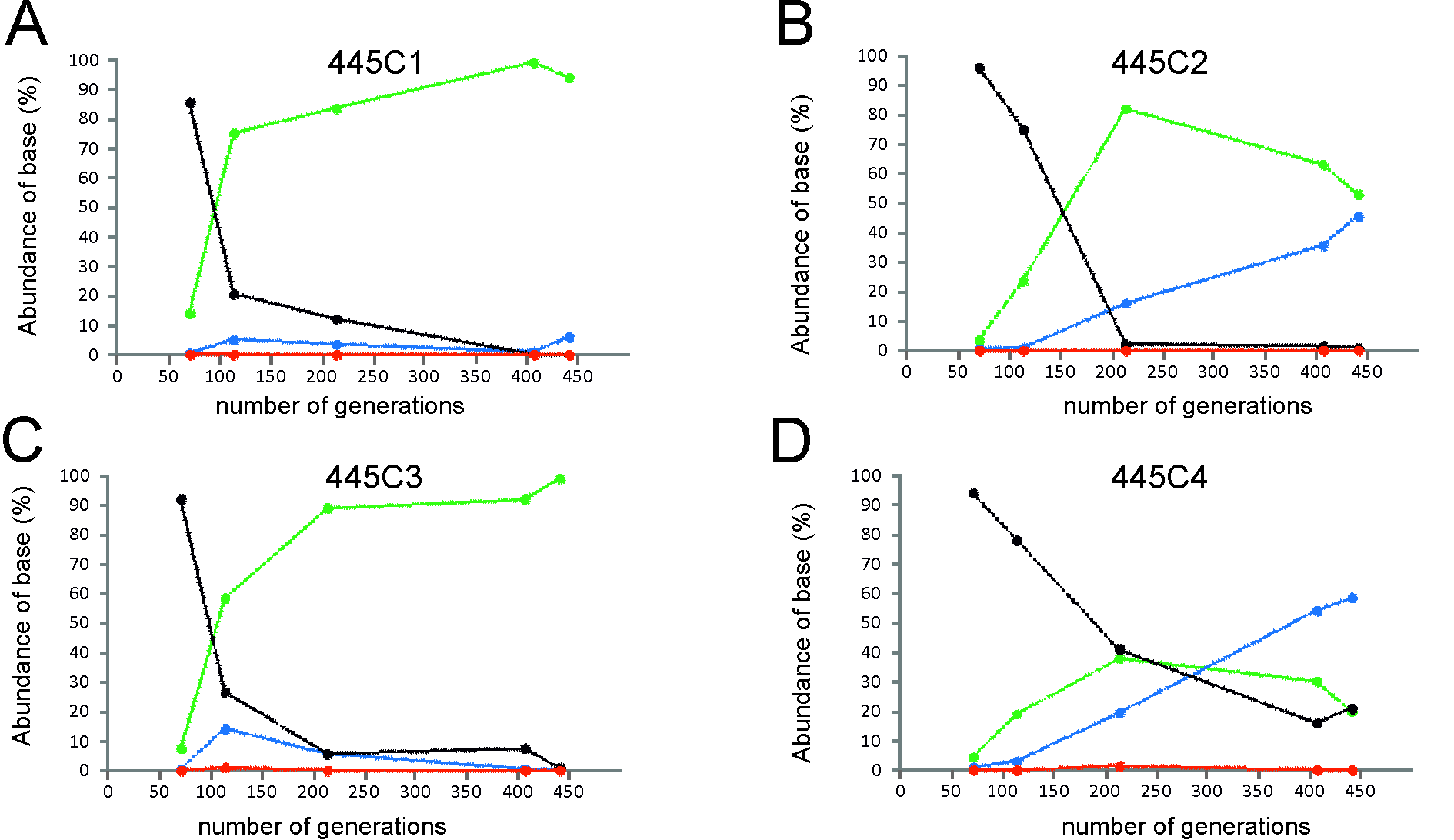

Supplement: Supplementary file 1 — Additional materials and methods and supplementary figures 1–9. (ZIP 12575 kb) [file 12862_2018_1331_MOESM1_ESM.zip › BMC Suppl Figure 7.tif]

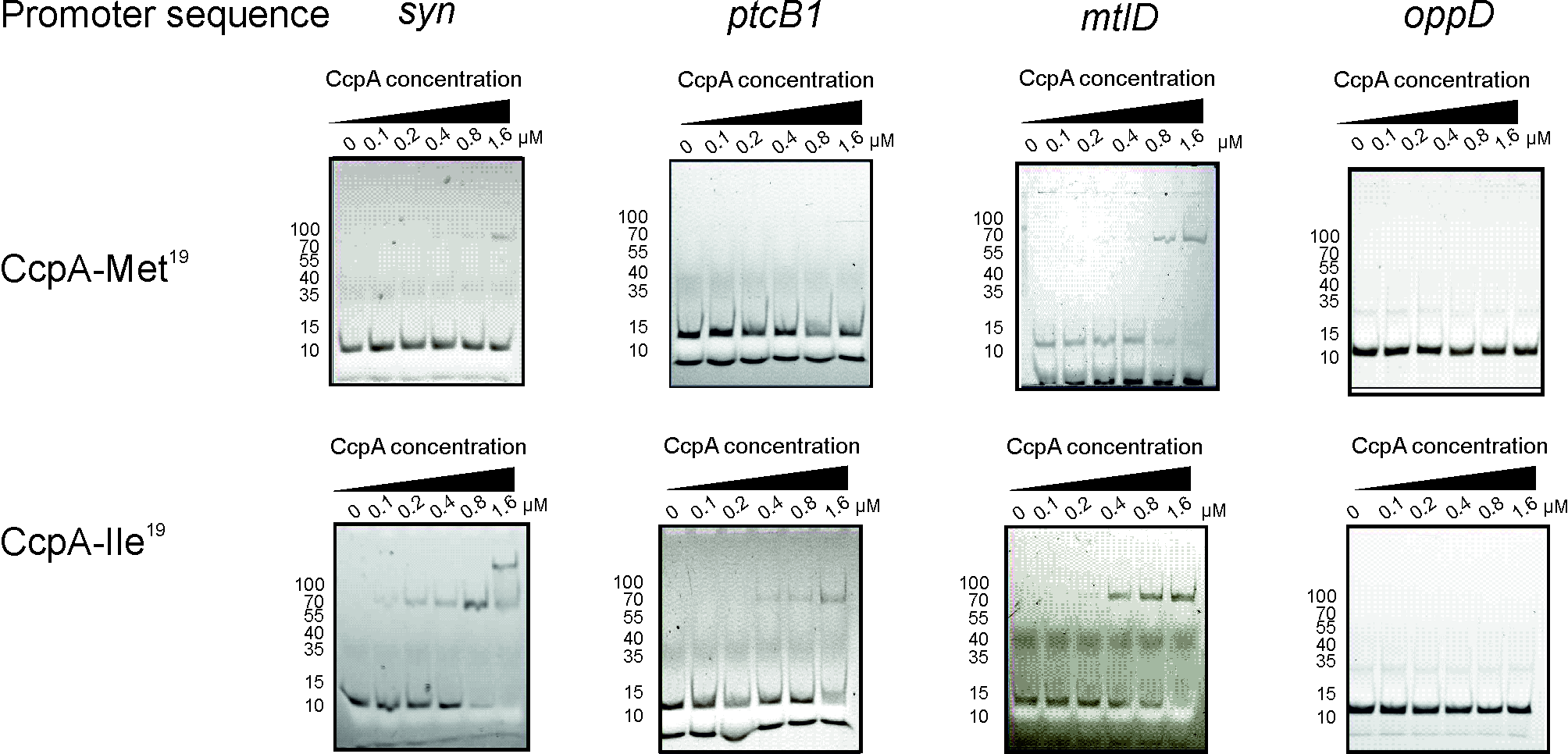

Supplement: Supplementary file 1 — Additional materials and methods and supplementary figures 1–9. (ZIP 12575 kb) [file 12862_2018_1331_MOESM1_ESM.zip › BMC Suppl Figure 6.tif]

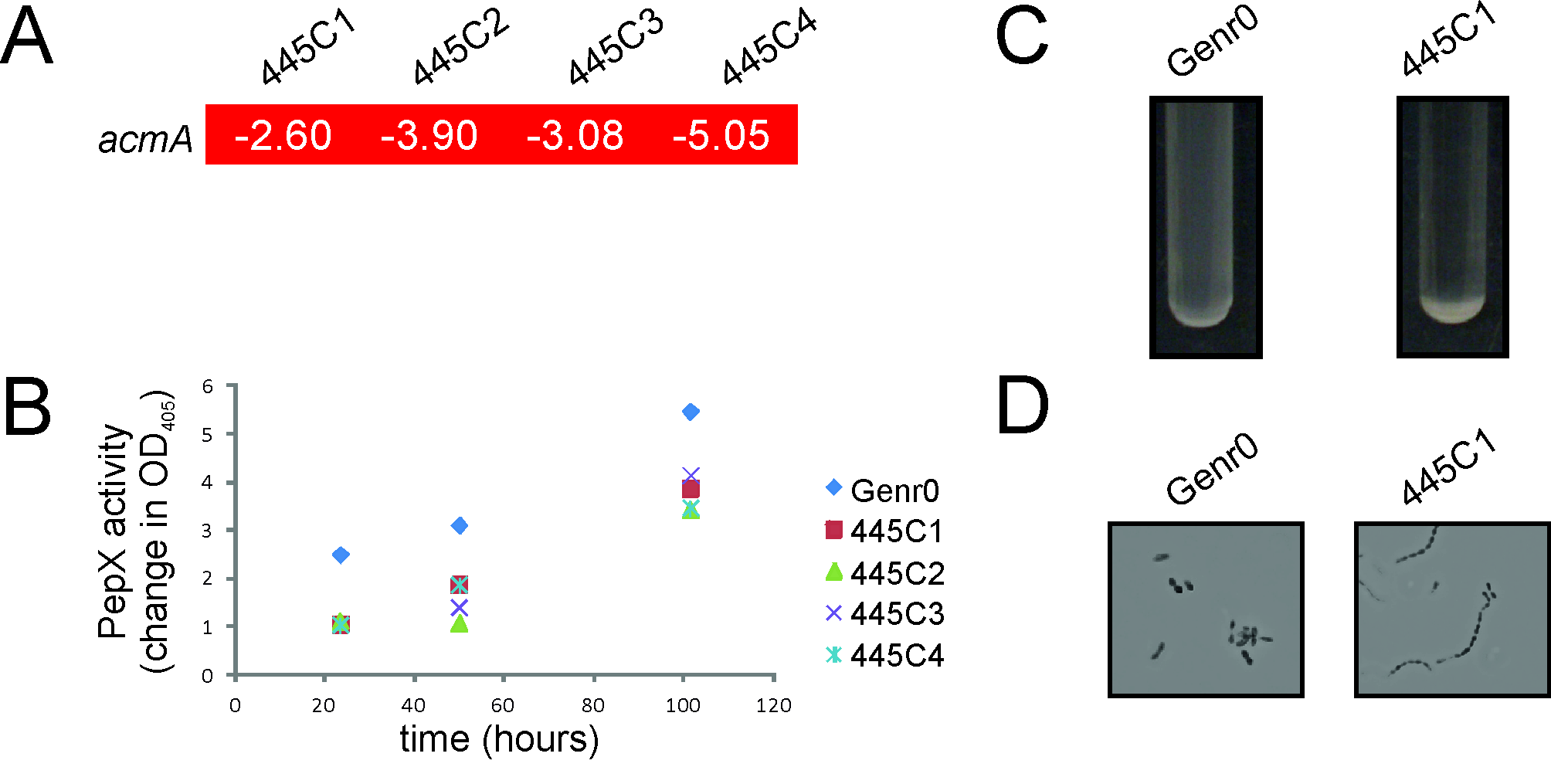

Supplement: Supplementary file 1 — Additional materials and methods and supplementary figures 1–9. (ZIP 12575 kb) [file 12862_2018_1331_MOESM1_ESM.zip › BMC Suppl Figure 5.tif]

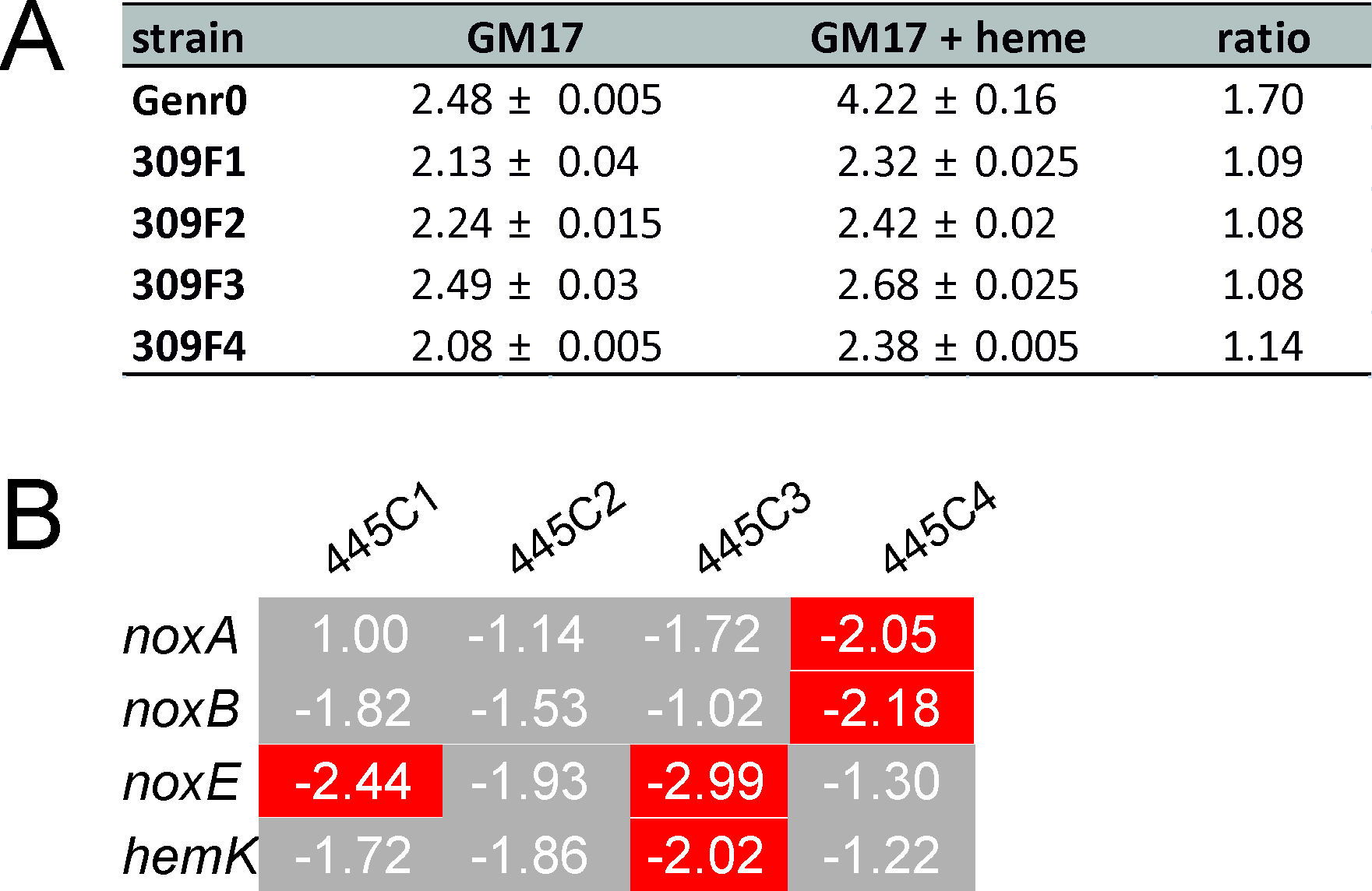

Supplement: Supplementary file 1 — Additional materials and methods and supplementary figures 1–9. (ZIP 12575 kb) [file 12862_2018_1331_MOESM1_ESM.zip › BMC Suppl Figure 4.tif]

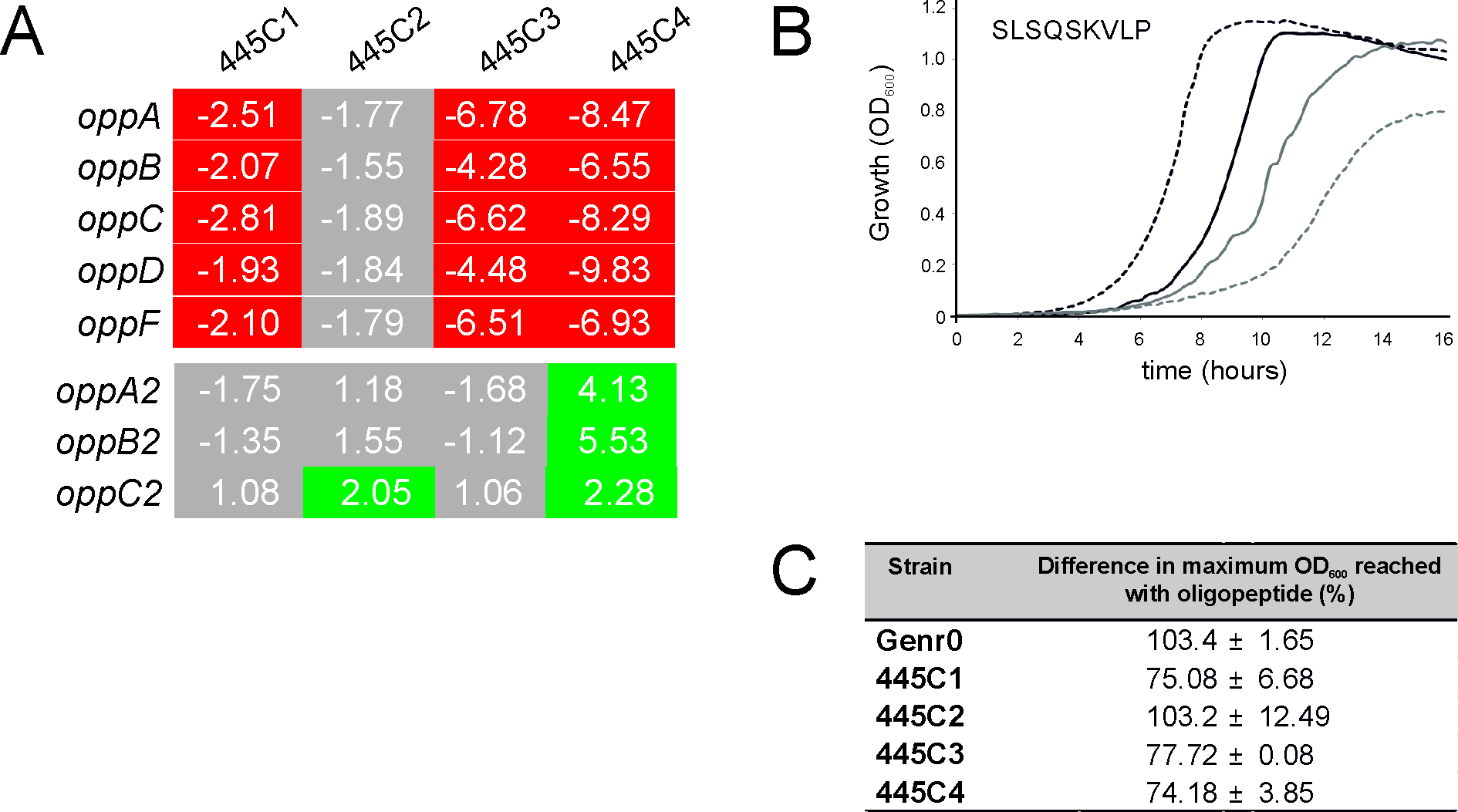

Supplement: Supplementary file 1 — Additional materials and methods and supplementary figures 1–9. (ZIP 12575 kb) [file 12862_2018_1331_MOESM1_ESM.zip › BMC Suppl Figure 3.tif]

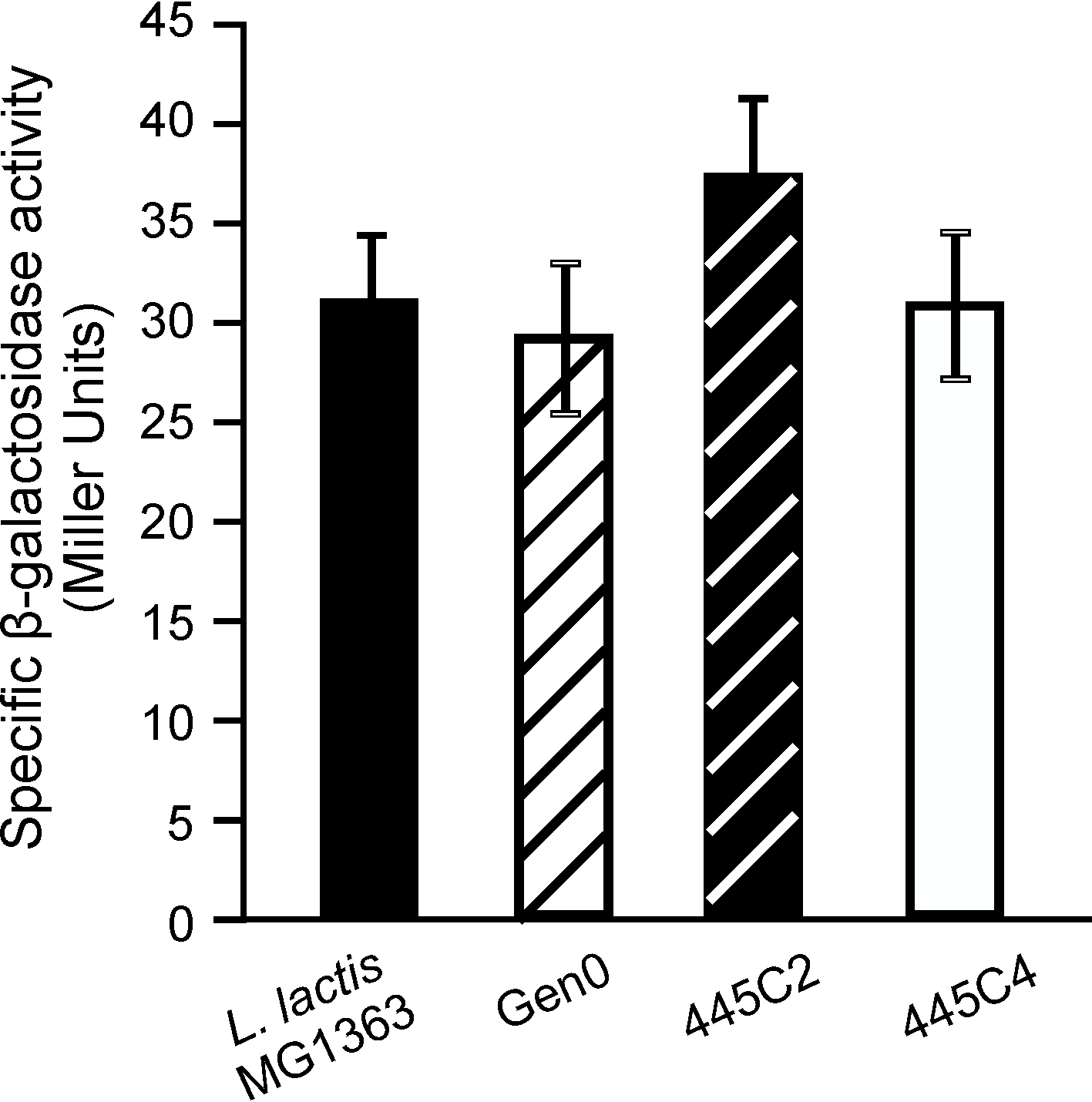

Supplement: Supplementary file 1 — Additional materials and methods and supplementary figures 1–9. (ZIP 12575 kb) [file 12862_2018_1331_MOESM1_ESM.zip › BMC Suppl Figure 2.tif]

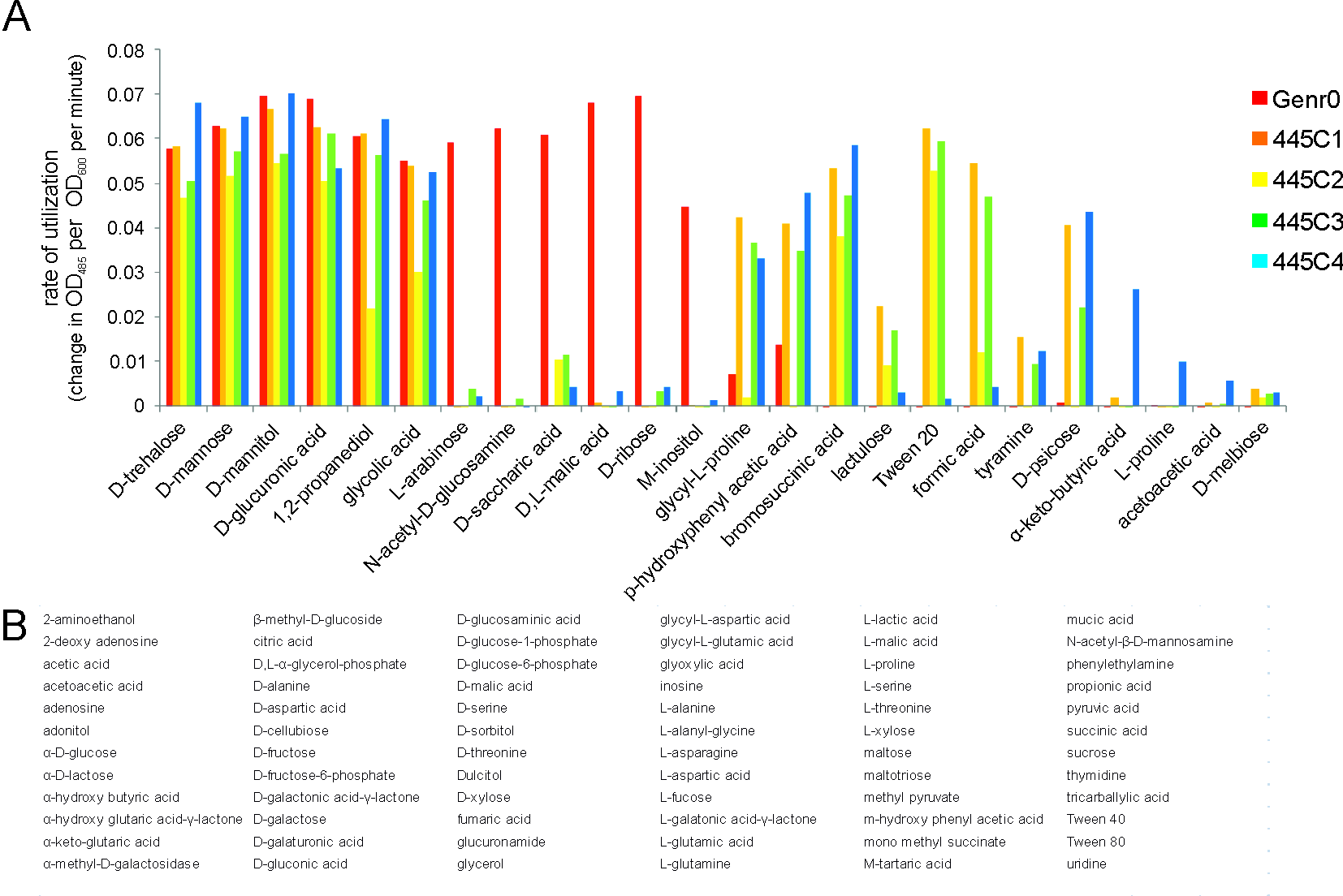

Supplement: Supplementary file 1 — Additional materials and methods and supplementary figures 1–9. (ZIP 12575 kb) [file 12862_2018_1331_MOESM1_ESM.zip › BMC Suppl Figure 1.tif]

**A**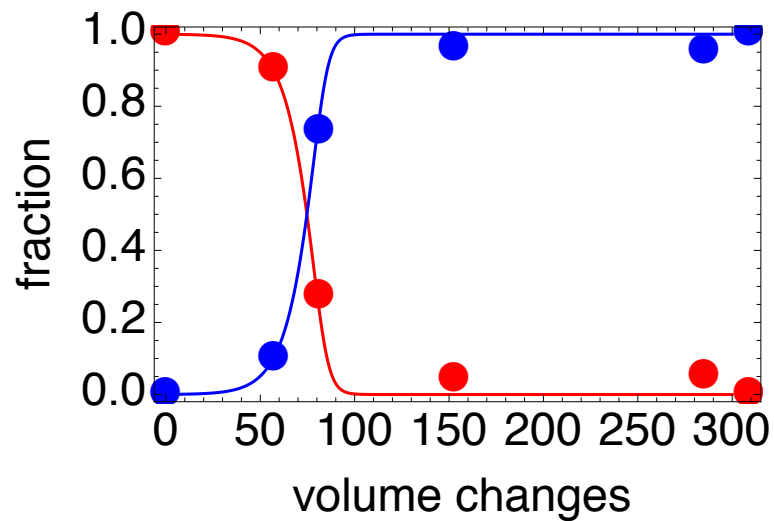**B**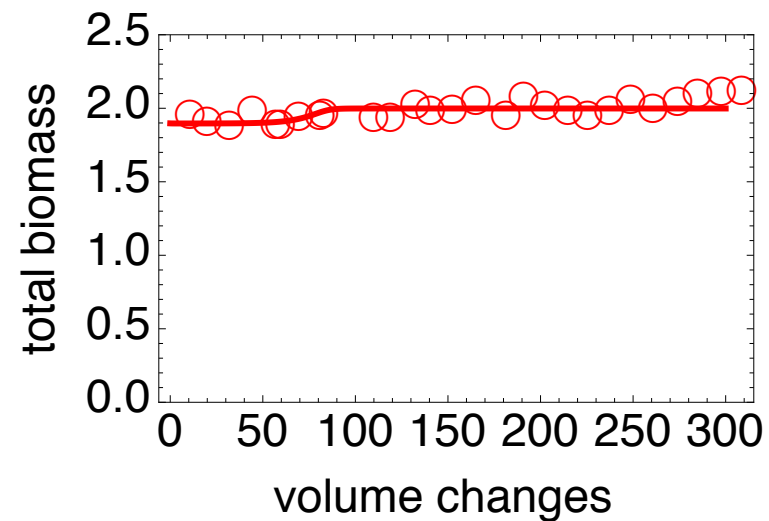**C**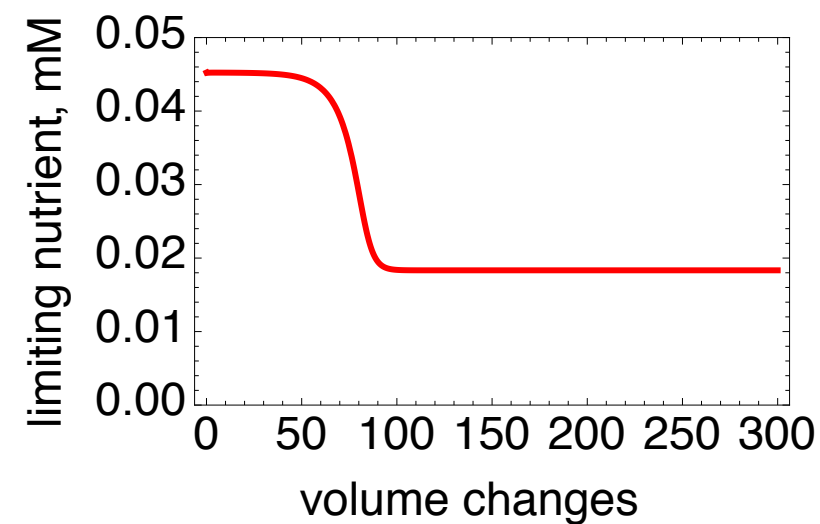**D**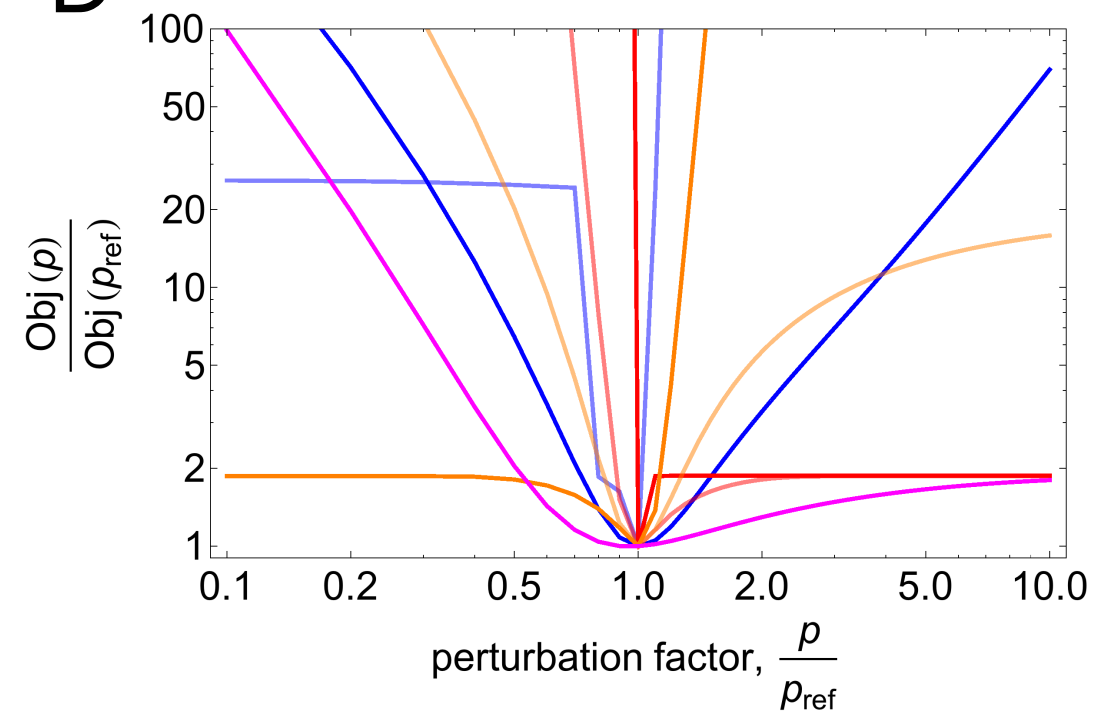**E**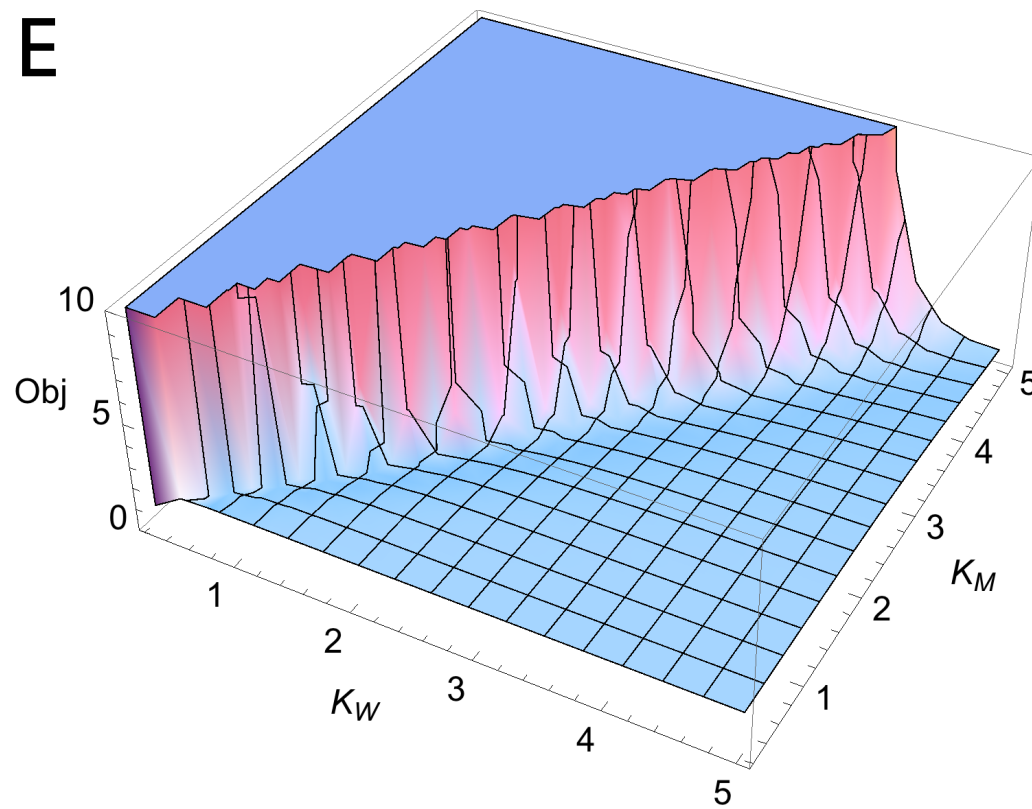

Supplement: Supplementary file 1 — Additional materials and methods and supplementary figures 1–9. (ZIP 12575 kb) [file 12862_2018_1331_MOESM1_ESM.zip › 180710 Suppl Figure 8.pdf]
