# Supplementary material for: Adaption to glucose limitation is modulated by the pleotropic regulator CcpA, independent of selection pressure strength
Source: BMC Evol Biol. 2019 Jan 10;19:15. doi: 10.1186/s12862-018-1331-x (PMC6327505; doi:10.1186/s12862-018-1331-x)
Supplement: Supplementary file 2 — Table S1. Preparation of chemically defined medium for prolonged cultivations (CDMPC) for Lactococcus lactis. (DOCX 29 kb) [file 12862_2018_1331_MOESM2_ESM.docx]

**Additional file 2: Table S1**. Preparation of chemically defined medium for prolonged cultivations (CDMPC) for *Lactococcus lactis*

| **A. Reagents** |  |  |  |  |  |  |
| --- | --- | --- | --- | --- | --- | --- |
| Buffer | MW | CAS | mg/l | mM | Supplier | Cat nº |
| glucose-monohydrate | 198.17 | 14431-43-7 | 4950.00 | 24.979 |  |  |
| Potassium phosphate monobasic | 136.09 | 7778-77-0 | 2750.00 | 20.207 | Sigma | P5655 |
| Sodium chloride | 58.44 | 7647-14-5 | 2900.00 | 49.624 | Sigma | S5886 |
| Sodium phosphate dibasic | 141.96 | 7558-79-4 | 2850.00 | 20.076 | Sigma | S5136 |
|  | | | | | | |
| Vitamins | MW | CAS | mg/l | uM | Supplier | Cat nº |
| (±)-α-Lipoic acid or DL-6,8-Thioctic acid | 206.33 | 1077-28-7 | 2.00 | 9.69 | Sigma | T1395 |
| D-Pantothenic acid hemicalcium salt | 238.27 | 137-08-6 | 0.50 | 2.10 | Sigma | P5155 |
| Biotin | 244.31 | 58-85-5 | 0.10 | 0.41 | Sigma | B4639 |
| Nicotinic acid | 123.11 | 59-67-6 | 1.00 | 8.12 | Sigma | N0761 |
| Pyridoxal hydrochloride | 203.62 | 65-22-5 | 1.00 | 4.91 | Sigma | P6155 |
| Pyridoxine hydrochloride (Pyridoxol.HCl) | 205.64 | 58-56-0 | 1.00 | 4.86 | Sigma | P6280 |
| Thiamine hydrochloride | 337.27 | 67-03-8 | 1.00 | 2.96 | Sigma | T1270 |
|  | | | | | | |
| Metals | MW | CAS | mg/l | uM | Supplier | Cat nº |
| Ammonium molybdate tetrahydrate | 1235.86 | 12054-85-2 | 0.30 | 0.24 | Sigma | M1019 |
| Calcium chloride dihydrate | 147.02 | 10035-04-8 | 3.00 | 20.41 | Sigma | C7902 |
| Cobalt(II) sulfate heptahydrate | 281.10 | 10026-24-1 | 0.30 | 1.07 | Sigma-Aldrich | C6768 |
| Copper(II) sulfate pentahydrate | 249.68 | 7758-99-8 | 0.30 | 1.20 | Sigma | C8027 |
| Iron(II) chloride tetrahydrate | 198.81 | 13478-10-9 | 4.00 | 20.12 | Sigma-Aldrich | 44939 |
| Magnesium chloride hexahydrate | 203.30 | 7791-18-6 | 200.00 | 983.76 | Sigma | M2393 |
| Manganese chloride tetrahydrate | 197.91 | 13446-34-9 | 4.00 | 20.21 | Sigma-Aldrich | M8054 |
| Zinc sulfate heptahydrate | 287.56 | 7446-20-0 | 0.30 | 1.04 | Sigma-Aldrich | Z0251 |
|  | | | | | | |
| Amino acids | MW | CAS | mg/l | mM | Supplier | Cat nº |
| L-Alanine | 89.09 | 56-41-7 | 130 | 1.4592 | Sigma | A7469 |
| L-Arginine | 174.20 | 74-79-3 | 244 | 1.4007 | Sigma | A8094 |
| L-Asparagine | 132.12 | 70-47-3 | 80 | 0.6055 | Sigma | A4159 |
| L-Aspartic acid | 133.10 | 56-84-8 | 137 | 1.0293 | Sigma | A7219 |
| L-Cysteine hydrochloride monohydrate | 175.63 | 7048-04-6 | 61 | 0.3473 | Sigma-Aldrich | C6852 |
| L-Glutamic acid | 147.13 | 56-86-0 | 97 | 0.6593 | Sigma | G8415 |
| L-Glutamine | 146.14 | 56-85-9 | 96 | 0.6569 | Sigma | G8540 |
| Glycine | 75.07 | 56-40-6 | 29 | 0.3863 | Sigma | G8790 |
| L-Histidine | 155.15 | 71-00-1 | 24 | 0.1547 | Sigma | H6034 |
| L-Isoleucine | 131.17 | 73-32-5 | 82 | 0.6251 | Sigma | I7403 |
| L-Leucine | 131.17 | 61-90-5 | 117 | 0.8920 | Sigma | L8912 |
| L-Lysine monohydrochloride | 182.65 | 657-27-2 | 187 | 1.0238 | Sigma | L8662 |
| L-Methionine | 149.21 | 63-68-3 | 38 | 0.2547 | Sigma | M5308 |
| L-Phenylalanine | 165.19 | 63-91-2 | 64 | 0.3874 | Sigma | P5482 |
| L-Proline | 115.13 | 147-85-3 | 412 | 3.5786 | Sigma | P5607 |
| L-Serine | 105.09 | 56-45-1 | 172 | 1.6367 | Sigma | S4311 |
| L-Threonine | 119.12 | 72-19-5 | 68 | 0.5709 | Sigma | T8441 |
| L-Tryptophan | 204.23 | 73-22-3 | 36 | 0.1763 | Sigma | T8941 |
| L-Tyrosine | 181.19 | 60-18-4 | 50 | 0.27595 | Sigma | T8566 |
| L-Valine | 117.15 | 72-18-4 | 86 | 0.7341 | Sigma | V0513 |
|  | | | | | | |
| **B. Reagent Setup** | | | | | | |
| 100x vitamin solution | | | | | | |
| 1\| Weigh the following reagents: | | | | | | |
| ·      200 mg (±)-α-Lipoic acid or DL-6,8-Thioctic acid (final conc. 0.969 mM) | | | | | | |
| ·      50 mg D-Pantothenic acid hemicalcium salt (final conc. 0.210 mM) | | | | | | |
| ·      100 mg Nicotinic acid  (final conc. 0.812 mM) | | | | | | |
| ·      100 mg Pyridoxal hydrochloride (final conc. 0.491 mM) | | | | | | |
| ·      100 mg Pyridoxine hydrochloride (Pyridoxol.HCl) (final conc. 0.486 mM) | | | | | | |
| ·      100 mg Thiamine hydrochloride (final conc. 0.296 mM) | | | | | | |
| 2\| Dissolve in approx. 900 mL of slightly heated dH2O (≤ 40º C). CRITICAL: Avoid unnecessary exposure to heat or light, since some vitamins are light and/or temperature sensitive. | | | | | | |
| 3\| Briefly increase the pH to >9.0 with 2.5 N NaOH while stirring thoroughly, ensuring that all vitamins are dissolved, and then lower it again to pH 7.0 with 2.5 N HCl. CRITICAL: Lower the pH as soon as there is no visible precipitate, since some vitamins are degraded with prolonged exposure to an alkaline pH. | | | | | | |
| 4\| Adjust volume to 1000 mL once all compounds are dissolved. | | | | | | |
| 5\| Aliquot as desired and store at -20º C (use within 6 months). | | | | | | |
|  | | | | | | |
| 100x metal solution | | | | | | |
| 1\| Weigh the following reagents: | | | | | | |
| ·      30 mg Ammonium molybdate tetrahydrate (final conc. 0.024 mM) | | | | | | |
| ·      300 mg Calcium chloride dihydrate (final conc. 2.041 mM) | | | | | | |
| ·      30 mg Cobalt(II) sulfate heptahydrate (final conc. 0.107 mM) | | | | | | |
| ·      30 mg Copper(II) sulfate pentahydrate (final conc. 0.120 mM) | | | | | | |
| ·      20000 mg Magnesium chloride hexahydrate (final conc. 98.376 mM) | | | | | | |
| ·      400 mg Manganese chloride tetrahydrate (final conc. 2.021 mM) | | | | | | |
| ·      30 mg Zinc sulfate heptahydrate (final conc. 0.104 mM) | | | | | | |
| 2\| Dissolve in approx. 900 mL of dH2O ensuring that no precipitate is left. | | | | | | |
| 3\| Weigh 400 mg of Iron(II) chloride tetrahydrate (final conc. 2.012 mM). | | | | | | |
| 4\| Dissolve separately in approx. 20 mL of 18.5% HCl (dissolves readily) and combine with the previous solution containing the other metals. | | | | | | |
| 5\| Adjust volume to 1000 mL, aliquot as desired and store at -20º C (use within 6 months). | | | | | | |
|  | | | | | | |
| 100x alkaline solution | | | | | | |
| 1\| Weigh the following reagents: | | | | | | |
| ·      10 mg Biotin (final conc. 0.041 mM) | | | | | | |
| ·      5000 mg L-Tyrosine (final conc. 27.595 mM) | | | | | | |
| 2\| Dissolve in approx. 900 mL of dH2O increasing the pH to >10.2 until no precipitate is left. | | | | | | |
| 3\| Adjust volume to 1000 mL, aliquot as desired and store at -20º C (use within 6 months). | | | | | | |
|  | | | | | | |
| 10x amino acid solution | | | | | | |
| 1\| Weigh the following reagents: | | | | | | |
| ·      1.30 g L-Alanine (final conc. 14.592 mM) | | | | | | |
| ·      2.44 g L-Arginine  (final conc. 14.007 mM) | | | | | | |
| ·      0.80 g L-Asparagine (final conc. 6.055 mM) | | | | | | |
| ·      1.37 g L-Aspartic acid (final conc. 10.293 mM) | | | | | | |
| ·      0.61 g L-Cysteine hydrochloride monohydrate (final conc. 3.473 mM) | | | | | | |
| ·      0.97 g L-Glutamic acid (final conc. 6.593 mM) | | | | | | |
| ·      0.96 g L-Glutamine (final conc. 6.569 mM) | | | | | | |
| ·      0.29 g Glycine (final conc. 3.863 mM) | | | | | | |
| ·      0.24 g L-Histidine (final conc. 1.547 mM) | | | | | | |
| ·      0.82 g L-Isoleucine  (final conc. 6.251 mM) | | | | | | |
| ·      1.17 g L-Leucine (final conc. 8.920 mM) | | | | | | |
| ·      1.87 g L-Lysine monohydrochloride (final conc. 10.238 mM) | | | | | | |
| ·      0.38 g L-Methionine (final conc. 2.547 mM) | | | | | | |
| ·      0.64 g L-Phenylalanine (final conc. 3.874 mM) | | | | | | |
| ·      4.12 g L-Proline (final conc. 35.786 mM) | | | | | | |
| ·      1.72 g L-Serine (final conc. 16.367 mM) | | | | | | |
| ·      0.68 g L-Threonine (final conc. 5.709 mM) | | | | | | |
| ·      0.36 g L-Tryptophan (final conc. 1.763 mM) | | | | | | |
| ·      0.86 g L-Valine  (final conc. 7.341 mM) | | | | | | |
| 2\| Dissolve in approx. 900 mL of slightly heated dH2O (≤ 50º C). | | | | | | |
| 3\| Briefly increase the pH to >9.0 with 2.5 N NaOH while stirring thoroughly, ensuring that all amino acids are dissolved, and then lower it again to pH 7.0 with 2.5 N HCl. CRITICAL: Lower the pH as soon as there is no visible precipitate, since some amino acids are degraded with prolonged exposure to an alkaline pH. | | | | | | |
| 4\| Adjust volume to 1000 mL once all compounds are dissolved. | | | | | | |
| 5\| Aliquot as desired and store at -20º C (use within 6 months). | | | | | | |
|  | | | | | | |
| **C. Procedure** | | | | | | |
| Here we describe the preparation of 1 L of CDMPC. For the preparation of different volumes scale proportionally the amounts mentioned below. | | | | | | |
| 1\| Weigh the following constituents of the CDMPC buffer: | | | | | | |
| ·      4.95 g Glucose-monohydrate (final conc. 24.979 mM) | | | | | | |
| ·      2.75 g Potassium phosphate monobasic (final conc. 20.207 mM) | | | | | | |
| ·      2.90 g Sodium chloride (final conc. 49.624 mM) | | | | | | |
| ·      2.85 g Sodium phosphate dibasic (final conc. 20.076 mM) | | | | | | |
| 2\| Dissolve in approx. 850 mL of dH2O. CRITICAL: Ensure that all compounds are dissolved before the next step. | | | | | | |
| 3\| Add the following reagents to the buffer solution from step 2 while stirring thoroughly: | | | | | | |
| ·      100 mL of 10x amino acid solution. | | | | | | |
| ·      10 mL of 100x metal solution. | | | | | | |
| ·      10 mL of 100x vitamin solution. | | | | | | |
| ·      10 mL of 100x alkaline solution. | | | | | | |
| CRITICAL: Add all reagents in the order mentioned above and ensure that in between each addition no precipitate is formed | | | | | | |
| 4\| Adjust the final pH to 6.5 with the addition of 2.5 N HCl. | | | | | | |
| 5\| Adjust volume to 1000 mL and filter sterilize through a polyethersulfone (PES) membrane (or tested equivalent) with a pore size ≥0.22 mm. CRITICAL: If the filter used is not with a PES membrane, ensure that the type of membrane used does not introduce additional compounds to the filtrate, such as wetting agents. | | | | | | |
| 6\| Store protected from light at 4ºC and use within 4 weeks. | | | | | | |
