# Supplementary material for: Adaption to glucose limitation is modulated by the pleotropic regulator CcpA, independent of selection pressure strength
Source: BMC Evol Biol. 2019 Jan 10;19:15. doi: 10.1186/s12862-018-1331-x (PMC6327505; doi:10.1186/s12862-018-1331-x)
Supplement: Supplementary file 3 — Table S2. Single nucleotide polymorphisms (SNPs) detected in adapted strains. (DOCX 25 kb) [file 12862_2018_1331_MOESM3_ESM.docx]

**Additional file 3: Table S2.**  Single nucleotide polymorphisms (SNPs) detected  in adapted strains

| **Reference** | | **ORF** | **Gene** | **Protein** | **Linares *et al.,* 2010** | | **Genr0** | | **445C1** | | **445C2** | | **445C3** | | **445C4** | |
| --- | --- | --- | --- | --- | --- | --- | --- | --- | --- | --- | --- | --- | --- | --- | --- | --- |
| **Position^a^** | **nucleotide** |  |  |  | **nucleotide** | **amino acid substitution** | **nucleotide** | **amino acid substitution** | **nucleotide** | **amino acid substitution** | **nucleotide** | **amino acid substitution** | **nucleotide** | **amino acid substitution** | **nucleotide** | **amino acid substitution** |
| 164096 | A | llmg_0172 | *codY* | transcriptional repressor CodY | A |  | A |  | A |  | A |  | A |  | **T** | **Ile214Phe** |
| 490263 | G |  | Intergenic | upstream of *hllA* (DNA binding protein) | G |  | G |  | G |  | G |  | G |  | **A** |  |
| 599198 | G | llmg_0610 | *greA* | transcription elongation factor GreA | G |  | GAP |  | G |  | G |  | G |  | **A/G** | **Glu60Lys** |
| 669804 | T |  | Intergenic | upstream of llmg_0676 (acetyltransferase) | T |  | T |  | T |  | T |  | **C** |  | **T** |  |
| 734315 | G | llmg_0747 | *llrF* | two-component system regulator llrF | G |  | G |  | G |  | **T** | **Leu214Phe** | G |  | G |  |
| 764359 | T | llmg_0775 | *ccpA* | catabolite control protein A | T |  | T |  | T |  | T |  | T |  | **C** | **Met19Thr** |
| 764360 | G | llmg_0775 | *ccpA* | catabolite control protein A | G |  | G |  | **A** | **Met19Ile** | **C** | **Met19Ile** | **A** | **Met19Ile** |  |  |
| 1321265 | T |  | Intergenic | upstream of *xpt*(purine metabolism) | T |  | T |  | T |  | **G** |  | T |  | T |  |
| 1321266 | C |  | Intergenic | upstream of *xpt*(purine metabolism) | C |  | C |  | C |  | **T** |  | C |  | C |  |
| 1571094 | A | llmg_1597 | llmg_1597 | hypothetical protein (uknown function but may bind R | A |  | A |  | A |  | A |  | A |  | **G** | **Tyr3His** |
| 1963018 | T | llmg_1981 | *rpoC* | DNA-directed RNA polymerase subunit beta' | T |  | T |  | T |  | **G** | **Glu1143Asp** | T |  | T |  |
| ^a^NC_009004 | | |  |  |  |  |  |  |  |  |  |  |  |  |  |  |
| Bold text highlights SNPs detected | | | | |  |  |  |  |  |  |  |  |  |  |  |  |
