# Supplementary material for: Adaption to glucose limitation is modulated by the pleotropic regulator CcpA, independent of selection pressure strength
Source: BMC Evol Biol. 2019 Jan 10;19:15. doi: 10.1186/s12862-018-1331-x (PMC6327505; doi:10.1186/s12862-018-1331-x)
Supplement: Supplementary file 4 — Table S3. Genes/operons found to change significantly in at least three of the adapted strains compared to the original strain. Significant changes, indicated in bold, were considered for genes with a Bayes p-value score of less than 0.05 and a pfp value of less than 0.05. (DOCX 46 kb) [file 12862_2018_1331_MOESM4_ESM.docx]

**Additional file 4: Table S3.** Genes/operons  found to change significantly in at least three of the adapted strains compared to the original strain. Significant changes, indicated in bold, were considered for genes with a Bayes p-value score of less than 0.05 and a pfp value of less than 0.05.

| **Gene no.** | **Gene name** | **Protein** | **Fold change** | | | |
| --- | --- | --- | --- | --- | --- | --- |
|  |  |  | **445C1** | **445C2** | **445C3** | **445C4** |
| **PTS systems** | | | | | | |
| llmg_0022 | *mtlA* | PTS system, mannitol-specific IIBC component | **6.31** | **7.84** | **12.38** | **12.24** |
| llmg_0024 | *mtlF* | PTS system, mannitol-specific IIA component | **3.49** | **6.77** | **5.49** | **4.36** |
| llmg_0437 | *ptcB* | cellobiose-specific PTS system IIB component | **-1.88** | -1.90 | **-2.10** | **-2.37** |
| llmg_0438 | *ptcA* | cellobiose-specific PTS system IIA component | **-2.15** | **-2.40** | **-3.14** | **-2.81** |
| llmg_0454 |  | beta-glucoside-specific PTS system IIABC component (trehalose) | **-2.18** | **-2.36** | **-2.02** | **-1.83** |
| llmg_0727 | *ptnD* | mannose-specific PTS system component IID | 1.16 | 1.67 | **1.89** | **3.96** |
| llmg_0728 | *ptnC* | mannose-specific PTS system component IIC | 1.80 | 1.65 | 1.81 | **1.88** |
| llmg_0729 | *ptnAB* | PTS system, mannose-specific IIAB components | **1.84** | 1.82 | **2.05** | **1.98** |
| **ABC transporters** | | | | | | |
| llmg_0312 | *phnD* | phosphonate ABC transporter, phosphonate-binding protein | **-2.11** | **-1.93** | **-2.48** | **-2.59** |
| llmg_0313 | *phnC* | phosphonates import ATP-binding protein | **-2.09** | **-2.49** | **-2.43** | -1.78 |
| llmg_0314 | *phnB* | phosphonate transport system permease protein | -1.41 | **-2.37** | **-2.39** | **-2.04** |
| llmg_0315 |  | phosphonate ABC transporter permease | -1.51 | **-2.38** | **-2.34** | -1.42 |
| llmg_0345 | *cbiQ* | putative cobalt ABC transporter permease protein | **5.79** | **5.94** | **7.67** | -1.53 |
| llmg_0344 | cbiO | putative cobalt ABC transporter ATP-binding protein | **-2.10** | **-2.70** | -1.88 | -1.18 |
| llmg_0347 | *fhuB* | ferrichrome ABC transporter permease protein | **5.17** | **4.68** | **6.22** | **-2.68** |
| llmg_0446 | *msmK* | multiple sugar-binding transport ATP-binding protein | **-6.31** | **-2.59** | **-5.02** | **-5.42** |
| llmg_0490 |  | sugar transport system permease protein | **-5.34** | **-2.75** | **-5.38** | -1.02 |
| llmg_0697 | *oppD* | oligopeptide transport ATP-binding protein | **-1.93** | -1.84 | **-4.48** | **-9.83** |
| llmg_0698 | *oppF* | oligopeptide transport ATP-binding protein | **-2.10** | -1.79 | **-6.51** | **-6.93** |
| llmg_0699 | *oppB* | peptide transport system permease protein | **-2.07** | -1.55 | **-4.28** | **-6.55** |
| llmg_0700 | *oppC* | peptide transport system permease protein | **-2.81** | -1.89 | **-6.62** | **-8.29** |
| llmg_0701 | *oppA* | oligopeptide-binding protein oppA precursor | **-2.51** | -1.77 | **-6.78** | **-8.47** |
| llmg_0737 | *malG* | maltose ABC transporter permease protein | -1.63 | -1.02 | -1.74 | **-2.38** |
| llmg_0738 | *malF* | maltose transport system permease protein | **-2.06** | -1.57 | **-2.50** | **-2.59** |
| llmg_0739 | *malE* | maltose ABC transporter substrate binding protein | **-2.63** | -1.41 | **-2.62** | **-2.88** |
| llmg_1049 | *busAB* | glycine betaine-binding periplasmic protein precursor (binding protein for OpuA) | **-3.05** | **-2.28** | **-2.62** | -1.71 |
| **Other transporters** | | | | | | |
| llmg_0375 |  | amino acid permease | **-4.58** | **-12.45** | **-6.07** | 1.24 |
| llmg_0399 | *nha* | Na+/H+ antiporter | **2.13** | **3.78** | **4.03** | 1.53 |
| llmg_0535 | *gltS* | arginine-binding periplasmic protein 1 precursor | **-2.99** | **-7.79** | **-4.57** | -1.25 |
| llmg_0891 | *pyrP* | uracil permease | **-2.18** | **-2.44** | **-2.66** | -1.80 |
| llmg_1104 |  | drug-export protein (MFS) | **-3.49** | **-4.68** | **-4.44** | 1.32 |
| llmg_2446 | *lmrP* | multidrug resistance protein (MFS) | **-6.62** | **-5.80** | **-6.84** | 1.16 |
| **Amino acid metabolism** | | | | | | |
| llmg_0138 | *argG* | argininosuccinate synthase | **-5.98** | **-5.86** | **-7.28** | -1.31 |
| llmg_0139 | *argH* | argininosuccinate lyase | **-4.56** | **-8.18** | **-5.87** | -1.12 |
| llmg_0508 |  | cysteine synthase | **4.14** | **3.74** | **4.29** | -1.30 |
| llmg_0536 | *argE* | acetylornithine deacetylase | **-3.78** | **-8.65** | **-4.78** | 1.13 |
| llmg_1757 | *argJ* | bifunctional ornithine acetyltransferase/N-acetylglutamate synthase protein | **-2.33** | **-7.44** | **-3.46** | 1.14 |
| llmg_1758 | *argC* | N-acetyl-gamma-glutamyl-phosphate reductase | **-2.85** | **-12.76** | **-5.14** | 1.07 |
| **Sugar metabolism (glycolysis, pentose phosphate pathway, fermentation, Leloir pathway)** | | | | | | |
| llmg_0025 | *mtlD* | mannitol-1-phosphate 5-hydrogenase | **2.98** | **8.22** | **6.58** | **3.18** |
| llmg_0455 | *trePP* | putative trehalose/maltose hydrolase | **-2.14** | **-3.09** | **-2.77** | -1.58 |
| llmg_0740 | *dexC* | neopullulanase | **-2.35** | **-2.42** | **-3.17** | -1.54 |
| llmg_0742 | *maa* | maltose O-acetyltransferase | **-3.19** | **-3.12** | **-3.87** | -1.71 |
| llmg_0745 | *mapA* | maltose phosphorylase | **-2.07** | **-2.16** | **-2.47** | **-2.25** |
| llmg_0751 | *ascB* | 6-phospho-beta-glucosidase | **-2.74** | **-3.45** | **-2.33** | **-2.05** |
| llmg_2321 | *poxL* | pyruvate oxidase | **2.04** | **2.18** | **2.77** | 1.28 |
| **Pyrimidine metabolism** | | | | | | |
| llmg_0762 | *udk* | uridine kinase | **-2.27** | **-2.84** | **-2.40** | 1.29 |
| llmg_0890 | *pyrR* | bifunctional pyrimidine regulatory protein PyrR uracil phosphoribosyltransferase | **-1.86** | **-2.66** | **-2.18** | **-2.59** |
| llmg_0893 | *pyrB* | aspartate carbamoyltransferase catalytic subunit | **-2.32** | 1.16 | **-2.54** | **-2.31** |
| llmg_0894 | *carA* | carbamoyl phosphate synthase small subunit | **-2.20** | **-2.08** | **-2.49** | **-2.26** |
| llmg_1508 | *pyrC* | dihydroorotase | **-2.60** | **-2.45** | **-2.29** | **-2.67** |
| llmg_1509 | *pyrE* | orotate phosphoribosyltransferase | **-2.06** | -1.71 | -1.47 | **-2.35** |
| llmg_1720 | *udp* | uridine phosphorylase | **-2.00** | 1.19 | **-2.21** | -1.50 |
| **Transcriptional regulators** | | | | | | |
| llmg_0023 | *mtlR* | transcriptional regulator mtl operon | **7.18** | **8.18** | **10.83** | **9.19** |
| llmg_0435 | *hexR* | putative HTH-type transcriptional regulator | **-2.71** | **-2.72** | **-2.44** | 1.07 |
| llmg_0439 |  | LacI family transcriptional regulator | **-5.90** | **-12.80** | **-10.29** | **-2.39** |
| llmg_0775 | *ccpA* | catabolite control protein A | **-2.31** | **-2.38** | **-2.96** | -1.46 |
| llmg_1224 |  | transcriptional regulator | **-2.00** | **-2.14** | **-2.70** | -1.14 |
| llmg_1247 | *arsD* | arsenical resistance operon trans-acting repressor arsD | **-1.85** | **-3.14** | **-2.20** | -1.22 |
| llmg_1576 | *hrcA* | heat-inducible transcription repressor | **2.80** | 1.30 | **2.14** | **3.58** |
| llmg_0340 | *plpD* | D-methionine-binding lipoprotein plpD precursor | **2.65** | **5.43** | **4.61** | -1.01 |
| **Chaperone** | | | | | | |
| llmg_0309 | *gcp* | putative DNA-binding/iron metalloprotein/AP endonuclease | **2.36** | **2.00** | **2.63** | 1.25 |
| llmg_0410 | *groES* | co-chaperonin | **2.11** | -1.00 | **2.12** | **2.15** |
| **Cell envelope** | | | | | | |
| llmg_0162 |  | hypothetical protein (bacterial adhesion, collagen binding domain, predicted signal sequence) | **1.99** | **2.27** | **2.81** | **8.97** |
| llmg_0280 | *acmA* | N-acetylglucosaminidase | **-2.60** | **-3.90** | **-3.08** | **-5.05** |
| llmg_1091 |  | putative secreted protein | **-2.02** | **-2.96** | **-2.32** | -1.64 |
| **Oxidative stress** | |  |  |  |  |  |
| llmg_0201 | *msrB* | methionine sulfoxide reductase B | **-3.12** | **-2.22** | **-2.08** | -1.56 |
| **DNA replication, transcription and translation** | | | | | | |
| llmg_0384 | *rluE* | ribosomal large subunit pseudouridine synthase | **-2.20** | **-7.08** | **-3.09** | 1.17 |
| llmg_0409 | *ssbA* | single-stranded DNA-binding protein | **-2.32** | **-3.06** | **-3.45** | -1.07 |
| llmg_0768 | *holA* | DNA polymerase III subunit delta | **-2.03** | **-2.67** | **-2.77** | -1.37 |
| llmg_1208 | *rplL* | 50S ribosomal protein L7/L12 | **-1.91** | **-2.26** | **-2.25** | -1.49 |
| llmg_1399 | *orf10 (engB)* | ribosome biogenesis GTP-binding protein YsxC | **2.47** | **3.44** | **4.73** | **5.90** |
| llmg_2035 | *gidA* | tRNA uridine 5-carboxymethylaminomethyl  modification enzyme GidA | **-2.31** | -1.60 | **-2.05** | **-2.88** |
| llmg_2354 | *rpoA* | DNA-directed RNA polymerase subunit alpha | **-2.05** | **-2.65** | **-2.90** | -1.83 |
| llmg_2488 | *ruvA* | Holliday junction DNA helicase RuvA | **3.67** | **6.78** | **5.97** | -1.19 |
| llmg_2523 | *recG* | ATP-dependent DNA helicase RecG | **6.58** | **7.41** | **9.72** | -1.94 |
| **Transposable elements** | | | | | | |
| llmg_0674 | *tnp1297* | transposase for insertion sequence element IS1297 | **2.30** | **4.92** | **3.30** | **4.91** |
| llmg_1371 | *matR* | maturase | **1.87** | **1.98** | 1.53 | **2.34** |
| **Peptidases** | | | | | | |
| llmg_0702 | *pepO* | endopeptidase | **-2.27** | -1.87 | **-5.49** | **-5.86** |
| llmg_2226 |  | M16 family peptidase | **-2.96** | **-2.86** | **-2.72** | **2.56** |
| **Aerobic growth** | | | | | | |
| llmg_2321 | *poxL* | pyruvate oxidase | **2.04** | **2.18** | **2.77** | 1.28 |
| llmg_2510 | *mutT* | putative mutator protein | -1.80 | **-2.85** | **-2.69** | **-2.74** |
| **Oxido/reductases** | | | | | | |
| llmg_0146 |  | aryl-alcohol dehydrogenase | **-1.86** | **-2.30** | **-2.16** | **-2.19** |
| llmg_0160 |  | oxygen-insensitive NAD(P)H nitroreductase | **1.88** | **2.23** | **2.09** | -1.09 |
| **Miscellaneous** | | | | | | |
| llmg_0734 |  | putative amidase | 1.42 | **2.02** | **2.26** | **2.68** |
| llmg_0995 |  | hydrolase, haloacid dehalogenase-like family protein | **-2.27** | **-2.16** | **-2.99** | -1.25 |
| llmg_1531 | *ribB* | riboflavin synthase subunit alpha | **4.48** | **7.74** | **4.31** | -1.00 |
| llmg_1572 | *mycA* | myosin-cross-reactive antigen | **3.14** | **4.02** | **3.17** | 1.18 |
| llmg_2089 | *ps453* | phage tail component with transglycosylase domain | **-2.53** | **-8.17** | **-4.50** | -1.11 |
| **No significant similarity/inferred function** | | | | | | |
| llmg_0034 | *ps122* | hypothetical protein | **-2.02** | **-2.34** | **-2.80** | -1.27 |
| llmg_0526 |  | hypothetical protein | **1.91** | **2.24** | **2.03** | **5.36** |
| llmg_0675 |  | hypothetical protein | **2.13** | **4.86** | **2.88** | **4.37** |
| llmg_0755 |  | hypothetical protein | **-2.14** | -1.54 | **-2.67** | -1.11 |
| llmg_1092 |  | hypothetical protein | **-2.78** | **-4.62** | **-2.76** | **-2.01** |
| llmg_1096 |  | hypothetical protein | **-2.36** | **-3.03** | **-2.54** | -1.52 |
| llmg_1108 |  | hypothetical protein | **-1.99** | -1.90 | **-2.06** | -1.53 |
| llmg_1186 |  | hypothetical protein | **-2.57** | **-2.23** | **-2.21** | -1.28 |
| llmg_1659 |  | hypothetical protein | **5.96** | **4.86** | **5.30** | **2.67** |
| llmg_1912 |  | hypothetical protein | **1.94** | 1.98 | **2.37** | **4.45** |
| llmg_2431 |  | hypothetical protein | **-3.92** | **-3.28** | **-2.95** | -1.45 |
| llmg_2465 |  | hypothetical protein | **2.48** | **2.71** | **2.24** | **2.56** |
| llmg_2515 |  | hypothetical protein | **-2.88** | **-7.78** | **-6.15** | **-2.45** |
| **Pseudogenes** | |  |  |  |  |  |
| llmg_pseudo59 | | pseudogene | **-2.50** | **-3.11** | **-3.87** | -1.22 |
| llmg_0396 | *pseudo* | pseudogene | **2.50** | **5.84** | **5.88** | -1.09 |
