# Supplementary material for: Adaption to glucose limitation is modulated by the pleotropic regulator CcpA, independent of selection pressure strength
Source: BMC Evol Biol. 2019 Jan 10;19:15. doi: 10.1186/s12862-018-1331-x (PMC6327505; doi:10.1186/s12862-018-1331-x)
Supplement: Supplementary file 5 — Table S4. Changes in gene regulation in the adapted strains compared to the original strain. Significant changes, indicated in bold, were considered for genes with a Bayes p-value score of less than 0.05 and a pfp value of less than 0.05. (DOCX 115 kb) [file 12862_2018_1331_MOESM5_ESM.docx]

**Additional file 5: Table S4**. Changes in gene regulation in the adapted strains compared to the original strain. Significant changes, indicated in bold, were considered for genes with a Bayes p-value score of less than 0.05 and a pfp value of less than 0.05.

| **Gene no.** | | **Gene name** | **Protein** | **Fold change** | | | |
| --- | --- | --- | --- | --- | --- | --- | --- |
|  |  |  |  | **445C1** | **445C2** | **445C3** | **445C4** |
| **Amino acid metabolism** | | |  |  |  |  |  |
|  | llmg_0138 | *argG* | argininosuccinate synthase | **-5.98** | **-5.86** | **-7.28** | -1.31 |
|  | llmg_0139 | *argH* | argininosuccinate lyase | **-4.56** | **-8.18** | **-5.87** | -1.12 |
|  | llmg_0185 |  | acetyltransferase | -1.34 | **-3.54** | -1.64 | -1.31 |
|  | llmg_0508 |  | cysteine synthase | **4.14** | **3.74** | **4.29** | -1.30 |
|  | llmg_0536 | *argE* | acetylornithine deacetylase | **-3.78** | **-8.65** | **-4.78** | 1.13 |
|  | llmg_0676 |  | acetyltransferase | 1.63 | **3.63** | 1.91 | **4.04** |
|  | llmg_0930 | *thiL* | ThiL protein | 1.57 | **2.34** | 1.33 | -1.14 |
|  | llmg_1219 | *dltA* | D-alanine--poly(phosphoribitol) ligase subunit 1 | -1.82 | **-2.31** | **-2.06** | 1.28 |
|  | llmg_1277 | *ilvC* | ketol-acid reductoisomerase | 1.05 | 1.07 | 1.00 | **2.12** |
|  | llmg_1278 | *ilvH (ilvN)* | acetolactate synthase 3 regulatory subunit | 1.18 | 1.33 | 1.23 | **1.98** |
|  | llmg_1279 | *ilvB* | acetolactate synthase catalytic subunit | 1.29 | 1.58 | 1.22 | **2.40** |
|  | llmg_1280 | *ilvD* | dihydroxy-acid dehydratase | 1.32 | **2.00** | 1.50 | **2.07** |
|  | llmg_1282 | *leuD* | isopropylmalate isomerase small subunit | -1.02 | -1.61 | -1.06 | 1.40 |
|  | llmg_1284 | *leuC* | isopropylmalate isomerase large subunit | 1.09 | **-2.02** | 1.28 | **3.05** |
|  | llmg_1288 | *hisK* | histidinol-phosphatase | 1.16 | 1.34 | -1.17 | **3.39** |
|  | llmg_1289 | *hisI* | bifunctional phosphoribosyl-AMP cyclohydrolase/phosphoribosyl-ATP pyrophosphatase protein | -1.17 | -1.50 | **-3.46** | **4.27** |
|  | llmg_1290 | *hisF* | imidazole glycerol phosphate synthase subunit | 1.68 | 1.48 | 1.83 | **2.14** |
|  | llmg_1291 | *hisA* | 1-(5-phosphoribosyl)-5-[(5-phosphoribosylamino) methylideneamino] | 1.47 | 1.24 | -1.01 | **4.72** |
|  |  |  | imidazole-4-carboxamide isomerase |  |  |  |  |
|  | llmg_1292 | *hisH* | imidazole glycerol phosphate synthase subunit | 1.87 | 1.42 | 1.80 | **2.88** |
|  | llmg_1293 |  | hypothetical protein | 1.47 | 1.33 | 1.38 | **2.43** |
|  | llmg_1294 | *hisB* | imidazoleglycerol-phosphate dehydratase | 1.84 | 1.48 | 1.47 | **2.04** |
|  | llmg_1295 | *hisD* | histidinol dehydrogenase | -1.36 | -1.45 | **-2.96** | **4.14** |
|  | llmg_1296 | *hisG* | ATP phosphoribosyltransferase catalytic subunit | **2.32** | 1.62 | 1.80 | **3.14** |
|  | llmg_1297 | *hisZ* | ATP phosphoribosyltransferase regulatory subunit | **2.34** | 1.54 | 1.60 | **3.88** |
|  | llmg_1298 | *hisC* | histidinol-phosphate aminotransferase | 1.60 | 1.41 | 1.69 | **4.42** |
|  | llmg_1756 | *argD* | acetylornithine aminotransferase | -1.48 | **-5.44** | -1.98 | 1.71 |
|  | llmg_1757 | *argJ* | bifunctional ornithine acetyltransferase/N-acetylglutamate synthase protein | **-2.33** | **-7.44** | **-3.46** | 1.14 |
|  | llmg_1758 | *argC* | N-acetyl-gamma-glutamyl-phosphate reductase | **-2.85** | **-12.76** | **-5.14** | 1.07 |
|  | llmg_1926 | aroA | 3-phosphoshikimate 1-carboxyvinyltransferase | **2.26** | **3.86** | 1.58 | -1.33 |
|  | llmg_2181 | *metB1* | cystathionine gamma-synthase | 1.27 | **2.03** | 1.39 | 1.05 |
|  | llmg_2307 | *arcD2* | arginine/ornithine antiporter | -1.58 | -1.38 | -1.43 | **-2.01** |
|  | llmg_2308 | *arcT* | transaminase | -1.26 | **5.86** | 1.26 | **-1.93** |
|  | llmg_2309 | *arcC2* | carbamate kinase | -1.38 | -1.08 | -1.31 | **-2.56** |
|  | llmg_2310 | *arcC1* | carbamate kinase | -1.16 | 1.20 | -1.26 | **-2.30** |
|  | llmg_2311 | *arcD1* | arginine/ornithine antiporter | -1.25 | -1.14 | -1.56 | **-2.80** |
|  | llmg_2312 | *arcB* | ornithine carbamoyltransferase | -1.84 | -1.27 | -1.79 | **-3.01** |
|  | llmg_2313 | *arcA* | arginine deiminase | -1.36 | **-2.62** | -1.53 | -1.52 |
|  | llmg_pseu | *leuB* | pseudogene | 1.05 | 1.28 | 1.07 | **1.52** |
|  | llmg_pseu | *leuA* | pseudogene | 1.01 | 1.06 | -1.07 | **1.87** |
| **Cell envelope** | |  |  |  |  |  |  |
|  | **Cell wall synthesis/hydrolysis** | | |  |  |  |  |
|  | llmg_0134 |  | bactoprenol glucosyl transferase | -1.03 | **3.56** | 1.05 | -1.10 |
|  | llmg_0280 | *acmA* | N-acetylglucosaminidase | **-2.60** | **-3.90** | **-3.08** | **-5.05** |
|  | llmg_0360 | *ddl* | D-alanyl-alanine synthetase A | **-2.21** | -1.98 | **-2.46** | -1.12 |
|  | llmg_0412 | vicX | ribonuclease Z | **2.17** | 1.36 | 1.18 | 1.16 |
|  | llmg_0413 | *kinC* | sensor histidine kinase | -1.63 | -1.06 | -1.61 | **-2.07** |
|  | llmg_0760 |  | transglycosylase | 1.71 | 1.24 | 1.33 | **2.18** |
|  | llmg_0851 | *ps356* | endolysin | -1.05 | **2.32** | 1.96 | -1.29 |
|  | llmg_1678 | *mraY* | phospho-N-acetylmuramoyl-pentapeptide-transferase | **2.10** | 1.46 | **2.20** | 1.43 |
|  | llmg_2165 | *acmB* | N-acetylmuramoyl-L-alanine amidase | -1.24 | -1.49 | -1.93 | **3.83** |
| **Cell membrane synthesis** | | |  |  |  |  |  |
|  | llmg_0119 |  | putative acyltransferase | 1.48 | **2.39** | **2.13** | -1.06 |
|  | llmg_0627 | *fadD* | long-chain acyl-CoA synthetase | -1.62 | -1.03 | **-4.35** | 1.20 |
|  | llmg_0935 |  | putative lysophospholipase | -1.77 | -1.78 | **-2.24** | 1.21 |
|  | **Inlvolved in secretion/secreted proteins** | | |  |  |  |  |
|  | llmg_0162 |  | hypothetical protein | **1.99** | **2.27** | **2.81** | **8.97** |
|  | llmg_0601 |  | putative secreted protein | -1.51 | **-2.26** | -1.80 | -1.38 |
|  | llmg_1091 |  | putative secreted protein | **-2.02** | **-2.96** | **-2.32** | -1.64 |
|  | llmg_1093 |  | putative secreted protein | -1.31 | **-2.32** | -1.69 | -1.32 |
|  | llmg_1127 |  | cell wall surface anchor family protein | -1.31 | **-2.72** | -1.49 | -1.13 |
|  | llmg_1449 | *srtA* | sortase | 1.48 | 1.62 | 1.68 | **2.46** |
|  | llmg_1650 |  | putative secreted protein | **2.22** | -1.00 | **2.27** | -1.66 |
| **Central metabolism** | | |  |  |  |  |  |
|  | **Glycolysis** |  |  |  |  |  |  |
|  | llmg_0264 | *fbp* | fructose-bisphosphatase | **-2.03** | -1.78 | **-2.33** | -1.56 |
|  | llmg_0530 | *gapA* | glyceraldehyde 3-phosphate dehydrogenase | 1.24 | 1.74 | **2.32** | 1.15 |
|  | llmg_1579 | *gpmB* | phosphoglycerate mutase | 1.83 | **2.09** | 1.94 | 1.16 |
|  | llmg_1923 | *gpmC* | phosphoglycerate mutase | 1.75 | **3.51** | 1.41 | -1.03 |
|  | **Conversion of sugars to glucose/glycolytic intermediates** | | |  |  |  |  |
|  | llmg_0025 | *mtlD* | mannitol-1-phosphate 5-hydrogenase | **2.98** | **8.22** | **6.58** | **3.18** |
|  | llmg_0266 |  | putative sugar kinase | **-2.20** | -1.14 | **-2.28** | 1.76 |
|  | llmg_0455 | *trePP* | putative trehalose/maltose hydrolase | **-2.14** | **-3.09** | **-2.77** | -1.58 |
|  | llmg_0456 | *pgmB* | beta-phosphoglucomutase | -1.81 | -1.72 | **-2.75** | -1.46 |
|  | llmg_0740 | *dexC* | neopullulanase | **-2.35** | **-2.42** | **-3.17** | -1.54 |
|  | llmg_0741 | *dexA* | oligo-1,6-alpha-glucosidase | -1.64 | **-2.02** | -1.99 | -1.76 |
|  | llmg_0742 | *maa* | maltose O-acetyltransferase | **-3.19** | **-3.12** | **-3.87** | -1.71 |
|  | llmg_0744 | *agl* | alpha-glucosidase | -1.41 | -1.23 | **-2.22** | -1.60 |
|  | llmg_0745 | *mapA* | maltose phosphorylase | **-2.07** | **-2.16** | **-2.47** | **-2.25** |
|  | llmg_0751 | *ascB* | 6-phospho-beta-glucosidase | **-2.74** | **-3.45** | **-2.33** | **-2.05** |
|  | llmg_2235 | *galK* | galactokinase | **2.46** | 1.22 | 1.17 | -1.24 |
|  | **Mixed acid fermentation** | | |  |  |  |  |
|  | llmg_0629 | *pfl* | formate acetyltransferase | **-2.52** | -1.12 | **-4.63** | -1.08 |
|  | llmg_1275 | *aldB* | alpha-acetolactate decarboxylase | 1.01 | 1.21 | 1.00 | **2.12** |
|  | llmg_1641 | *butA* | acetoin reductase | 1.05 | 1.02 | -1.03 | **2.53** |
|  | llmg_1642 | *butB* | 2,3-butanediol dehydrogenase | 1.07 | 1.05 | 1.13 | **2.03** |
|  | llmg_2289 | *ackA2* | acetate kinase | 1.47 | 1.84 | 1.45 | **-2.97** |
|  | **Glycogen synthesis** | |  |  |  |  |  |
|  | llmg_1873 | *glgD* | glucose-1-phosphate adenylyltransferase | **-2.21** | -1.91 | **-2.75** | 1.53 |
|  | llmg_0158 | *glgB* | glycogen branching enzyme | 1.48 | 1.69 | 1.74 | **2.59** |
|  | **Pentose Phosphate pathway** | | |  |  |  |  |
|  | llmg_0586 | *gnd* | 6-phosphogluconate dehydrogenase | **-2.73** | -1.20 | **-7.93** | -1.02 |
|  | **ATP synthesis** | |  |  |  |  |  |
|  | llmg_1948 | *atpA* | F0F1 ATP synthase subunit alpha | 1.75 | -1.06 | **2.37** | 1.30 |
|  | **Aerobic growth** | |  |  |  |  |  |
|  | llmg_0408 | *noxE* | NADH oxidase | **-2.44** | -1.93 | **-2.99** | -1.30 |
|  | llmg_0560 | *hemK* | putative protoporphyrinogen oxidase | -1.72 | -1.86 | **-2.02** | -1.22 |
|  | llmg_1734 | *noxB* | NADH dehydrogenase | -1.82 | -1.53 | -1.02 | **-2.18** |
|  | llmg_1735 | *noxA* | NADH dehydrogenase | 1.00 | -1.14 | -1.72 | **-2.05** |
|  | llmg_1916 |  | putative electron transport protein | 1.58 | 1.18 | **2.01** | **-2.48** |
|  | llmg_2321 | *poxL* | pyruvate oxidase | **2.04** | **2.18** | **2.77** | 1.28 |
|  | **Other oxido/reductases** | |  |  |  |  |  |
|  | llmg_0146 |  | aryl-alcohol dehydrogenase | **-1.86** | **-2.30** | **-2.16** | **-2.19** |
|  | llmg_0160 |  | oxygen-insensitive NAD(P)H nitroreductase | **1.88** | **2.23** | **2.09** | -1.09 |
| **DNA replication, transcription and translation** | | | |  |  |  |  |
|  | **DNA replication** | |  |  |  |  |  |
|  | llmg_0444 | *ligA* | NAD-dependent DNA ligase LigA | **1.98** | -1.46 | **3.12** | 1.00 |
|  | llmg_0768 | *holA* | DNA polymerase III subunit delta | **-2.03** | **-2.67** | **-2.77** | -1.37 |
|  | llmg_2409 | *polC* | DNA polymerase III PolC | 1.27 | 1.18 | 1.18 | **2.03** |
|  | **Homologous recombination** | | |  |  |  |  |
|  | llmg_0409 | *ssbA* | single-stranded DNA-binding protein | **-2.32** | **-3.06** | **-3.45** | -1.07 |
|  | llmg_2153 | *priA* | primosome assembly protein PriA | 1.04 | **6.89** | -1.18 | -1.15 |
|  | llmg_2488 | *ruvA* | Holliday junction DNA helicase RuvA | **3.67** | **6.78** | **5.97** | -1.19 |
|  | llmg_2523 | *recG* | ATP-dependent DNA helicase RecG | **6.58** | **7.41** | **9.72** | -1.94 |
|  | **Transcription/RNA degradation** | | |  |  |  |  |
|  | llmg_0369 | *rheA* | ATP-dependent RNA helicase | -1.82 | **2.56** | -1.78 | **-2.14** |
|  | llmg_0521 | *rpoD* | RNA polymerase sigma factor RpoD | 1.28 | **-2.28** | 1.52 | 1.35 |
|  | llmg_2354 | *rpoA* | DNA-directed RNA polymerase subunit alpha | **-2.05** | **-2.65** | **-2.90** | -1.83 |
|  | **Translation** |  |  |  |  |  |  |
|  | llmg_0007 |  | GTP-dependent nucleic acid-binding protein EngD | -1.62 | **-3.45** | -1.62 | 1.02 |
|  | llmg_0251 | *rpsU* | 30S ribosomal protein S21 | -1.80 | -1.67 | **-2.14** | -1.08 |
|  | llmg_0296 | *rpsD* | 30S ribosomal protein S4 | **-2.26** | 1.02 | -1.82 | -1.54 |
|  | llmg_0384 | *rluE* | ribosomal large subunit pseudouridine synthase | **-2.20** | **-7.08** | **-3.09** | 1.17 |
|  | llmg_0722 | *serS* | seryl-tRNA synthetase | 1.32 | 1.25 | 1.44 | **2.67** |
|  | llmg_0932 | *rpsP* | 30S ribosomal protein S16 | -1.46 | -1.76 | **-2.02** | -1.88 |
|  | llmg_1208 | *rplL* | 50S ribosomal protein L7/L12 | **-1.91** | **-2.26** | **-2.25** | -1.49 |
|  | llmg_1399 | *orf10 (eng* | ribosome biogenesis GTP-binding protein YsxC | **2.47** | **3.44** | **4.73** | **5.90** |
|  | llmg_1477 | *glyS* | glycyl-tRNA synthetase subunit beta | -1.34 | -1.29 | -1.26 | 1.45 |
|  | llmg_1478 | *glyQ* | glycyl-tRNA synthetase subunit alpha | -1.30 | -1.25 | -1.50 | **-2.42** |
|  | llmg_1791 | *rbfA* | ribosome-binding factor A | -1.62 | **-2.34** | -1.36 | 1.10 |
|  | llmg_2035 | *gidA* | tRNA uridine 5-carboxymethylaminomethyl  modification enzyme GidA | **-2.31** | -1.60 | **-2.05** | **-2.88** |
|  | llmg_2078 | *rpsO* | 30S ribosomal protein S15 | -1.54 | -1.41 | **-1.43** | 1.10 |
|  | llmg_2277 | *rplK* | 50S ribosomal protein L11 | -1.79 | -1.47 | **-2.24** | -1.40 |
|  | llmg_2380 | *rplB* | 50S ribosomal protein L2 | **-3.12** | -1.40 | -1.30 | 1.21 |
|  | llmg_2382 | *rplD* | 50S ribosomal protein L4 | -1.56 | -1.18 | -1.14 | **-2.72** |
|  | llmg_2383 | *rplC* | 50S ribosomal protein L3 | -1.34 | -1.07 | -1.13 | **-2.15** |
|  | llmg_2384 | *rpsJ* | 30S ribosomal protein S10 | **-2.55** | -1.53 | **-2.10** | -1.88 |
|  | llmg_2430 | *rpsB* | 30S ribosomal protein S2 | **-2.11** | -1.92 | **-2.09** | -1.10 |
|  | llmg_2473 | *rpsR* | 30S ribosomal protein S18 | **-2.15** | -1.26 | **-2.09** | -1.30 |
|  | llmg_2475 | *rpsF* | 30S ribosomal protein S6 | 1.15 | -1.15 | 1.03 | **-2.06** |
|  | **DNA uptake** | |  |  |  |  |  |
|  | llmg_1370 | *ltrB* | group II intron-interrupted relaxase LtrB | -1.81 | **-2.06** | -1.79 | -1.10 |
|  | llmg_1483 | *comFA* | superfamily II DNA/RNA helicase | 1.05 | **3.75** | 1.08 | -1.48 |
|  | **Transposable elements** | |  |  |  |  |  |
|  | llmg_0674 | *tnp1297* | transposase for insertion sequence element IS1297 | **2.30** | **4.92** | **3.30** | **4.91** |
|  | llmg_0711 | *tnpR* | DNA-invertase/resolvase | 1.03 | -1.39 | -1.07 | **-2.47** |
|  | llmg_1371 | *matR* | maturase | **1.87** | **1.98** | 1.53 | **2.34** |
|  | llmg_1654 | *tnp981* | transposase for insertion sequence element IS981M | **2.08** | 1.28 | 1.41 | 1.69 |
|  | llmg_1893 | *tnp904* | transposase for insertion sequence IS904H | 1.63 | 1.23 | 1.53 | **2.27** |
|  | llmg_2493 | *tnp905* | transposase for insertion sequence element IS905N | **2.67** | 1.38 | 1.06 | -1.17 |
| **Peptidases** | |  |  |  |  |  |  |
|  | llmg_0702 | *pepO* | endopeptidase | **-2.27** | -1.87 | **-5.49** | **-5.86** |
|  | llmg_1357 | orf49 | hypothetical protein (oligoendopeptidase, pepF/M3 family) | -1.68 | **-2.55** | -1.78 | 1.11 |
|  | llmg_2226 |  | M16 family peptidase | **-2.96** | **-2.86** | **-2.72** | **2.56** |
| **Phosphoryl transfer** | | |  |  |  |  |  |
|  | llmg_0254 | *hadL* | cryptic haloacid dehalogenase 1 | 1.32 | -1.73 | 1.03 | **2.49** |
|  | llmg_0995 |  | hydrolase, haloacid dehalogenase-like family protein | **-2.27** | **-2.16** | **-2.99** | -1.25 |
|  | llmg_2034 |  | HAD superfamily hydrolase | 1.86 | 1.50 | **2.06** | 1.02 |
| **Purine/pyrimidine metabolism** | | | |  |  |  |  |
|  | llmg_0299 | *add* | adenosine deaminase | 1.49 | **2.09** | 1.60 | 1.84 |
|  | llmg_0316 | *cpdC* | 2',3'-cyclic-nucleotide 2'-phosphodiesterase | -1.34 | -1.02 | **-2.17** | -1.88 |
|  | llmg_0607 | *apt* | adenine phosphoribosyltransferase | -1.38 | -1.34 | -1.46 | -1.84 |
|  | llmg_0762 | *udk* | uridine kinase | **-2.27** | **-2.84** | **-2.40** | 1.29 |
|  | llmg_0776 | *trxB2* | TrxB2 protein | -1.15 | **-2.08** | -1.85 | -1.57 |
|  | llmg_0890 | *pyrR* | bifunctional pyrimidine regulatory protein PyrR uracil phosphoribosyltransferase | **-1.86** | **-2.66** | **-2.18** | **-2.59** |
|  | llmg_0891 | *pyrP* | uracil permease | **-2.18** | **-2.44** | **-2.66** | -1.80 |
|  | llmg_0893 | *pyrB* | aspartate carbamoyltransferase catalytic subunit | **-2.32** | 1.16 | **-2.54** | **-2.31** |
|  | llmg_0894 | *carA* | carbamoyl phosphate synthase small subunit | **-2.20** | **-2.08** | **-2.49** | **-2.26** |
|  | llmg_1063 | *cdd* | Cdd protein | **-1.89** | **-2.14** | -1.92 | -1.40 |
|  | llmg_1105 | *pyrK* | dihydroorotate dehydrogenase, electron transfer subunit | -1.15 | -1.47 | 1.02 | **-2.11** |
|  | llmg_1106 | *pyrDB* | dihydroorotate dehydrogenase 1B | -1.70 | -1.91 | -1.68 | **-1.77** |
|  | llmg_1107 | *pyrF* | orotidine 5'-phosphate decarboxylase | -1.63 | -1.77 | -1.81 | **-2.15** |
|  | llmg_1508 | *pyrC* | dihydroorotase | **-2.60** | **-2.45** | **-2.29** | **-2.67** |
|  | llmg_1509 | *pyrE* | orotate phosphoribosyltransferase | **-2.06** | -1.71 | -1.47 | **-2.35** |
|  | llmg_1541 | *nrdH* | glutaredoxin-like protein | -1.62 | -1.85 | **-2.06** | **-1.52** |
|  | llmg_1542 | *nrdI* | ribonucleotide reductase stimulatory protein | -1.75 | -1.72 | -1.71 | **-1.88** |
|  | llmg_1543 | *nrdE* | ribonucleotide-diphosphate reductase subunit alpha | -1.44 | -1.44 | -1.71 | **-2.30** |
|  | llmg_1544 | *nrdF* | ribonucleotide-diphosphate reductase subunit beta | -1.52 | -1.82 | -1.80 | **-1.93** |
|  | llmg_1720 | *udp* | uridine phosphorylase | **-2.00** | 1.19 | **-2.21** | -1.50 |
| **Stress response** | | |  |  |  |  |  |
|  | **Chaperones** |  |  |  |  |  |  |
|  | llmg_0410 | *groES* | co-chaperonin | **2.11** | -1.00 | **2.12** | **2.15** |
|  | llmg_0411 | *groEL* | chaperonin | 1.21 | 1.11 | 1.58 | **2.31** |
|  | llmg_1400 | orf9 | Hypothetical protein with DnaJ/HSP40 domain | 1.40 | **2.43** | **2.25** | 1.11 |
|  | llmg_1574 | *dnaK* | molecular chaperone | **2.43** | 1.20 | **2.12** | **1.90** |
|  | llmg_2502 | *dnaJ* | chaperonen protein | 1.50 | 1.32 | 1.75 | **1.86** |
|  | **Other** |  |  |  |  |  |  |
|  | llmg_0201 | *msrB* | methionine sulfoxide reductase B | **-3.12** | **-2.22** | **-2.08** | -1.56 |
|  | llmg_0528 | *clpE* | ATP-dependent Clp protease ATP-binding subunit clpE | **2.24** | 1.11 | 1.58 | 1.56 |
|  | llmg_1352 | *telA* | putative tellurium resistance protein | -1.87 | **-3.64** | **-2.15** | -1.19 |
|  | llmg_1489 | *phoH* | PhoH-like protein (starvation induced) | **2.49** | 1.22 | 1.68 | 1.15 |
|  | llmg_1575 | *grpE* | heat shock protein | **2.26** | -1.32 | 1.71 | 1.48 |
|  | llmg_2047 |  | universal stress protein E | -1.29 | **-2.16** | -1.39 | 1.44 |
| **Transcriptional regulators** | | |  |  |  |  |  |
|  | **Activators** |  |  |  |  |  |  |
|  | llmg_0274 |  | hypothetical protein (putative transcriptional regulator) | 1.26 | 1.12 | **4.05** | 1.33 |
|  | llmg_0350 | *fhuR* | HTH-type transcriptional regulator | -1.02 | -1.10 | -1.13 | **-2.38** |
|  | llmg_2512 | *rcfB* | transcriptional regulator | -1.95 | **1.09** | -1.52 | **-2.02** |
|  | **Repressors** |  |  |  |  |  |  |
|  | llmg_0435 | *hexR* | putative HTH-type transcriptional regulator | **-2.71** | **-2.72** | **-2.44** | 1.07 |
|  | llmg_0439 |  | LacI family transcriptional regulator | **-5.90** | **-12.80** | **-10.29** | **-2.39** |
|  | llmg_0775 | *ccpA* | catabolite control protein A | **-2.31** | **-2.38** | **-2.96** | -1.46 |
|  | llmg_1209 | *rmaX* | MarR family transcriptional regulator | 1.55 | 1.81 | 1.93 | **4.44** |
|  | llmg_1224 |  | transcriptional regulator | **-2.00** | **-2.14** | **-2.70** | -1.14 |
|  | llmg_1247 | *arsD* | arsenical resistance operon trans-acting repressor arsD | **-1.85** | **-3.14** | **-2.20** | -1.22 |
|  | llmg_1274 | *aldR* | putative regulator | -1.00 | 1.21 | 1.36 | **3.43** |
|  | llmg_1576 | *hrcA* | heat-inducible transcription repressor | **2.80** | 1.30 | **2.14** | **3.58** |
|  | llmg_2198 | *padR* | putative transcriptional repressor of PadC | 1.04 | -1.04 | -1.39 | 1.28 |
|  | **Antiterminator** | |  |  |  |  |  |
|  | llmg_0023 | *mtlR* | transcriptional regulator mtl operon | **7.18** | **8.18** | **10.83** | **9.19** |
|  | **Activator/repressor** | |  |  |  |  |  |
|  | llmg_0340 | *plpD* | D-methionine-binding lipoprotein plpD precursor | **2.65** | **5.43** | **4.61** | -1.01 |
|  | llmg_0414 | *llrC* | two-component system regulator | -1.29 | -1.41 | -1.52 | **-2.66** |
|  | llmg_0747 | *llrF* | two-component system regulator llrF | -1.75 | -1.62 | **-2.03** | 1.67 |
|  | llmg_1648 | *llrD* | two-component system regulator llrD | **2.58** | 1.06 | **2.43** | -1.26 |
|  | llmg_2067 | *rlrB* | LysR family transcriptional regulator | **3.38** | **2.11** | **2.80** | -1.15 |
|  | **Unknown** |  |  |  |  |  |  |
|  | llmg_0163 | *epsR* | transcriptional regulator | 1.31 | **2.47** | 1.64 | 1.78 |
|  | llmg_1868 |  | HTH-type transcriptional regulator | 1.26 | **2.91** | **2.09** | -1.30 |
|  | llmg_1983 | *codZ* | CodY family transcriptional regulator | -1.19 | **2.45** | 1.35 | **5.54** |
| **Transport systems** | | |  |  |  |  |  |
|  | **PTS systems** | |  |  |  |  |  |
|  | llmg_0022 | *mtlA* | PTS system, mannitol-specific IIBC component | **6.31** | **7.84** | **12.38** | **12.24** |
|  | llmg_0024 | *mtlF* | PTS system, mannitol-specific IIA component | **3.49** | **6.77** | **5.49** | **4.36** |
|  | llmg_0437 | *ptcB* | cellobiose-specific PTS system IIB component | **-1.88** | -1.90 | **-2.10** | **-2.37** |
|  | llmg_0438 | *ptcA* | cellobiose-specific PTS system IIA component | **-2.15** | **-2.40** | **-3.14** | **-2.81** |
|  | llmg_0453 |  | sucrose-specific PTS enzyme IIABC (likely trehalose specific) | -1.37 | -1.98 | -1.53 | **-1.86** |
|  | llmg_0454 |  | beta-glucoside-specific PTS system IIABC component (trehalose) | **-2.18** | **-2.36** | **-2.02** | **-1.83** |
|  | llmg_0727 | *ptnD* | mannose-specific PTS system component IID | 1.16 | 1.67 | **1.89** | **3.96** |
|  | llmg_0728 | *ptnC* | mannose-specific PTS system component IIC | 1.80 | 1.65 | 1.81 | **1.88** |
|  | llmg_0729 | *ptnAB* | PTS system, mannose-specific IIAB components | **1.84** | 1.82 | **2.05** | **1.98** |
|  | **ABC transporters** | |  |  |  |  |  |
|  | llmg_0269 |  | ABC transporter ATP binding and permease protein | 1.28 | 1.41 | 1.48 | **2.45** |
|  | llmg_0312 | *phnD* | phosphonate ABC transporter, phosphonate-binding protein | **-2.11** | **-1.93** | **-2.48** | **-2.59** |
|  | llmg_0313 | *phnC* | phosphonates import ATP-binding protein | **-2.09** | **-2.49** | **-2.43** | -1.78 |
|  | llmg_0314 | *phnB* | phosphonate transport system permease protein | -1.41 | **-2.37** | **-2.39** | **-2.04** |
|  | llmg_0315 |  | phosphonate ABC transporter permease | -1.51 | **-2.38** | **-2.34** | -1.42 |
|  | llmg_0344 | cbiO | putative cobalt ABC transporter ATP-binding protein | **-2.10** | **-2.70** | -1.88 | -1.18 |
|  | llmg_0345 | *cbiQ* | putative cobalt ABC transporter permease protein | **5.79** | **5.94** | **7.67** | -1.53 |
|  | llmg_0347 | *fhuB* | ferrichrome ABC transporter permease protein | **5.17** | **4.68** | **6.22** | **-2.68** |
|  | llmg_0446 | *msmK* | multiple sugar-binding transport ATP-binding protein | **-6.31** | **-2.59** | **-5.02** | **-5.42** |
|  | llmg_0525 |  | ABC transporter ATP binding protein | 1.22 | **2.07** | 1.49 | **4.34** |
|  | llmg_0697 | *oppD* | oligopeptide transport ATP-binding protein | **-1.93** | -1.84 | **-4.48** | **-9.83** |
|  | llmg_0698 | *oppF* | oligopeptide transport ATP-binding protein | **-2.10** | -1.79 | **-6.51** | **-6.93** |
|  | llmg_0699 | *oppB* | peptide transport system permease protein | **-2.07** | -1.55 | **-4.28** | **-6.55** |
|  | llmg_0700 | *oppC* | peptide transport system permease protein | **-2.81** | -1.89 | **-6.62** | **-8.29** |
|  | llmg_0701 | *oppA* | oligopeptide-binding protein oppA precursor | **-2.51** | -1.77 | **-6.78** | **-8.47** |
|  | llmg_0737 | *malG* | maltose ABC transporter permease protein | -1.63 | -1.02 | -1.74 | **-2.38** |
|  | llmg_0738 | *malF* | maltose transport system permease protein | **-2.06** | -1.57 | **-2.50** | **-2.59** |
|  | llmg_0739 | *malE* | maltose ABC transporter substrate binding protein | **-2.63** | -1.41 | **-2.62** | **-2.88** |
|  | | | | | | | |
|  | llmg_1049 | *busAB* | glycine betaine-binding periplasmic protein precursor (binding protein for OpuA) | **-3.05** | **-2.28** | **-2.62** | -1.71 |
|  | llmg_1202 |  | ABC transporter ABC binding and permease protein | -1.38 | -1.27 | -1.49 | **-2.21** |
|  | llmg_1203 |  | ABC transporter ABC binding and permease protein | -1.02 | -1.36 | -1.84 | **-1.96** |
|  | llmg_1512 |  | Putative ABC transporter ATP-binding protein | -1.02 | 1.15 | 1.11 | **-2.16** |
|  | llmg_1552 |  | putative ABC type transport system permease protein | 1.72 | 1.58 | **2.04** | **2.12** |
|  | llmg_1675 |  | ABC transporter ATP-binding protein | 1.90 | **2.86** | 1.37 | 1.80 |
|  | llmg_1676 |  | ABC transporter permease protein | **2.12** | 3.88 | 1.46 | **2.08** |
|  | llmg_2024 | *oppA2* | oligopeptide-binding protein | -1.75 | 1.18 | -1.68 | **4.13** |
|  | llmg_2025 | *oppC2* | oligopeptide transport system permease protein | 1.08 | **2.05** | 1.06 | **2.28** |
|  | llmg_2026 | *oppB2* | peptide transport system permease protein | -1.35 | 1.55 | -1.12 | **5.53** |
|  | llmg_2398 | *zitP* | zinc ABC transporter permease protein | 1.66 | 1.68 | **2.12** | 1.36 |
|  | llmg_2399 | *zitQ* | zinc ABC transporter ATP binding protein | 1.60 | 1.93 | **2.26** | 1.22 |
|  | llmg_2400 | *zitS* | zinc ABC transporter substrate binding protein | 1.79 | 1.93 | 1.71 | **2.01** |
|  | llmg_pseu | *oppD2* | pseudogene | 1.15 | 1.79 | 1.13 | 1.67 |
|  | **Other transporters** | |  |  |  |  |  |
|  | llmg_0070 |  | putative permease | **2.59** | 1.23 | **2.05** | -1.29 |
|  | llmg_0118 | *ctrA (bcaP)* | putative amino-acid transporter | **-2.17** | 1.27 | 1.01 | -1.10 |
|  | llmg_0137 |  | permease | -1.03 | **-2.25** | **-2.70** | -1.22 |
|  | llmg_0140 | *pmrB* | multidrug resistance efflux pump (MFS) | -1.49 | **-2.56** | -1.31 | 1.76 |
|  | llmg_0330 |  | putative permease | -1.79 | -1.82 | **-2.78** | -1.38 |
|  | llmg_0375 |  | amino acid permease | **-4.58** | **-12.45** | **-6.07** | 1.24 |
|  | llmg_0376 |  | amino acid permease | -1.44 | **-2.96** | -1.32 | 1.33 |
|  | llmg_0386 | *lysQ* | amino-acid permease LysQ | 1.33 | **-2.60** | 1.88 | **8.97** |
|  | llmg_0399 | *nha* | Na+/H+ antiporter | **2.13** | **3.78** | **4.03** | 1.53 |
|  | llmg_0490 |  | sugar transport system permease protein | **-5.34** | **-2.75** | **-5.38** | -1.02 |
|  | llmg_0535 | *gltS* | arginine-binding periplasmic protein 1 precursor | **-2.99** | **-7.79** | **-4.57** | -1.25 |
|  | llmg_0547 | *ctpE* | cation transporter E1-E2 family ATPase (HAD domain) | **-2.15** | **2.04** | -1.30 | -1.07 |
|  | llmg_0631 | *pmrA* | multidrug resistance efflux pump (MFS) | **-2.27** | 1.47 | **-3.29** | 1.16 |
|  | llmg_1104 |  | drug-export protein (MFS) | **-3.49** | **-4.68** | **-4.44** | 1.32 |
|  | llmg_1178 | *gadC* | putative glutamate/gamma-aminobutyrate antiporter | 1.77 | 1.56 | **2.40** | -1.27 |
|  | llmg_1210 |  | multidrug resistance protein | 1.06 | 1.95 | 1.43 | **3.03** |
|  | llmg_1474 |  | putative voltage gated chloride channel | 1.14 | **2.38** | 1.50 | 1.01 |
|  | llmg_2171 | *mntA* | putative Mn and Fe transporters | **1.87** | **2.02** | 1.19 | 1.00 |
|  | llmg_2446 | *lmrP* | multidrug resistance protein (MFS) | **-6.62** | **-5.80** | **-6.84** | 1.16 |
|  | llmg_pseu | *dppP* | pseudogene | -1.09 | **2.25** | 1.45 | **2.16** |
| **Vitamin/cofactor synthesis** | | | |  |  |  |  |
|  | llmg_1131 | *panE* | 2-dehydropantoate 2-reductase | 1.43 | **2.35** | 1.67 | 1.62 |
|  | llmg_1336 | *folP* | dihydropteroate synthase | **2.00** | **2.24** | 1.71 | -1.18 |
|  | llmg_1470 | *nadE* | NAD synthetase | 1.06 | **-2.55** | 1.43 | 1.18 |
|  | llmg_1530 | ribA | riboflavin biosynthesis protein ribA | **2.17** | 1.34 | 1.37 | 1.08 |
|  | llmg_1531 | *ribB* | riboflavin synthase subunit alpha | **4.48** | **7.74** | **4.31** | -1.00 |
| **Miscellaneous** | |  |  |  |  |  |  |
|  | llmg_0309 | *gcp* | putative DNA-binding/iron metalloprotein/AP endonuclease | **2.36** | **2.00** | **2.63** | 1.25 |
|  | llmg_0734 |  | putative amidase | 1.42 | **2.02** | **2.26** | **2.68** |
|  | llmg_1572 | *mycA* | myosin-cross-reactive antigen | **3.14** | **4.02** | **3.17** | 1.18 |
|  | llmg_1905 |  | putative esterase | 1.01 | **3.08** | 1.16 | -1.35 |
|  | llmg_2189 | *tmrB* | putative tunicamycin resistance protein | -1.01 | **-2.19** | -1.66 | -1.08 |
|  | llmg_2197 | *padC* | probable phenolic acid decarboxylase | -1.32 | -1.60 | -1.30 | **2.67** |
|  | llmg_2199 | *chiC* | acidic endochitinase precursor | -1.43 | -1.94 | -1.51 | **-2.93** |
|  | llmg_2510 | *mutT* | putative mutator protein | -1.80 | **-2.85** | **-2.69** | **-2.74** |
| **Phage associated genes** | | |  |  |  |  |  |
|  | llmg_0028 | *ps128* | hypothetical protein | -1.46 | **-2.47** | **-2.15** | -1.11 |
|  | llmg_0034 | *ps122* | hypothetical protein | **-2.02** | **-2.34** | **-2.80** | -1.27 |
|  | llmg_0035 | *ps121* | putative DNA binding protein | -1.01 | -1.94 | **-3.08** | -1.20 |
|  | llmg_0036 | *ps120* | hypothetical protein | 1.67 | 1.19 | **2.53** | 1.12 |
|  | llmg_0041 | *ps115* | phage repressor | 1.59 | **3.55** | 1.91 | -1.33 |
|  | llmg_0598 | *ps205* | cI-like repressor | -1.04 | **-2.46** | 1.13 | -1.04 |
|  | llmg_0802 | *ps312* | hypothetical protein | -1.10 | **-2.29** | -1.75 | -1.08 |
|  | llmg_2089 | *ps453* | phage tail component with transglycosylase domain | **-2.53** | **-8.17** | **-4.50** | -1.11 |
|  | llmg_2130 | *ps413* | hypothetical protein | 1.64 | **2.06** | 1.54 | 1.15 |
|  | llmg_2141 | *ps402* | hypothetical protein | 1.63 | 1.55 | **2.01** | -1.30 |
| **No significant similarity/inferred function** | | | |  |  |  |  |
|  | llmg_0018 |  |  | **2.17** | 1.95 | **2.17** | 1.21 |
|  | llmg_0099 |  | hypothetical protein | **-2.13** | 1.01 | **-1.96** | -1.70 |
|  | llmg_0131 |  | hypothetical protein | 1.83 | **3.53** | -1.04 | -1.23 |
|  | llmg_0165 |  | hypothetical protein | 1.31 | **-2.56** | -1.39 | -1.21 |
|  | llmg_0183 |  | hypothetical protein | -1.15 | -1.86 | **-2.12** | -1.55 |
|  | llmg_0247 |  | hypothetical protein | **2.26** | 1.51 | 1.51 | 1.68 |
|  | llmg_0258 |  | hypothetical protein | -1.25 | **-2.33** | -1.25 | 1.12 |
|  | llmg_0266 |  | hypothetical protein | **-2.20** | -1.13 | **-2.28** | 1.76 |
|  | llmg_0268 |  | hypothetical protein | **-2.27** | -1.02 | -1.79 | **4.05** |
|  | llmg_0387 |  | hypothetical protein | -1.33 | **-7.68** | **-2.72** | -1.45 |
|  | llmg_0526 |  | hypothetical protein | **1.91** | **2.24** | **2.03** | **5.36** |
|  | llmg_0541 |  | hypothetical protein | **-2.06** | 1.70 | -1.94 | -1.42 |
|  | llmg_0668 |  | hypothetical protein | -1.21 | 1.36 | **-2.12** | -1.35 |
|  | llmg_0673 |  | hypothetical protein | 1.10 | **2.52** | 1.51 | 1.55 |
|  | llmg_0675 |  | hypothetical protein | **2.13** | **4.86** | **2.88** | **4.37** |
|  | llmg_0679 |  | hypothetical protein | -1.65 | **-2.23** | **-2.14** | -1.08 |
|  | llmg_0685 |  | hypothetical protein | 1.03 | -1.96 | **-2.96** | -1.48 |
|  | llmg_0705 |  | hypothetical protein | -1.50 | **-2.10** | -1.46 | -1.68 |
|  | llmg_0750 |  | hypothetical protein | 1.39 | **2.42** | 1.64 | 1.15 |
|  | llmg_0755 |  | hypothetical protein | **-2.14** | -1.54 | **-2.67** | -1.11 |
|  | llmg_0939 |  | hypothetical protein | -1.36 | **-2.11** | -1.33 | -1.08 |
|  | llmg_1024 |  | hypothetical protein | 1.04 | **-2.18** | -1.67 | **-2.24** |
|  | llmg_1061 |  | hypothetical protein | -1.10 | **-2.05** | -1.75 | -1.79 |
|  | llmg_1089 |  | hypothetical protein | **-2.85** | **-2.10** | -1.79 | -1.83 |
|  | llmg_1090 |  | hypothetical protein | **-1.95** | **-2.50** | -1.89 | -1.68 |
|  | llmg_1092 |  | hypothetical protein | **-2.78** | **-4.62** | **-2.76** | **-2.01** |
|  | llmg_1094 |  | hypothetical protein | -1.75 | **-3.51** | **-2.36** | -1.51 |
|  | llmg_1095 |  | hypothetical protein | -1.74 | **-3.30** | **-2.27** | -1.74 |
|  | llmg_1096 |  | hypothetical protein | **-2.36** | **-3.03** | **-2.54** | -1.52 |
|  | llmg_1108 |  | hypothetical protein | **-1.99** | -1.90 | **-2.06** | -1.53 |
|  | llmg_1186 |  | hypothetical protein | **-2.57** | **-2.23** | **-2.21** | -1.28 |
|  | llmg_1211 |  | hypothetical protein | 1.75 | 1.89 | **2.20** | **10.00** |
|  | llmg_1229 |  | hypothetical protein | -1.77 | **-2.70** | **-3.04** | -1.27 |
|  | llmg_1230 |  | hypothetical protein | 1.29 | **2.08** | 1.48 | 1.19 |
|  | llmg_1259 |  | hypothetical protein | 1.19 | -1.92 | **-2.12** | 1.16 |
|  | llmg_1301 |  | hypothetical protein | -1.12 | -1.62 | **-2.03** | **-2.51** |
|  | llmg_1302 |  | hypothetical protein | -1.52 | **-2.20** | **-2.21** | -1.94 |
|  | llmg_1303 |  | hypothetical protein | -1.40 | -0.29 | **-2.41** | **-2.25** |
|  | llmg_1306 |  | hypothetical protein, putative adhesin precursor | 1.05 | **-3.29** | -1.26 | -1.31 |
|  | llmg_1344 |  | hypothetical protein | 1.02 | **-3.44** | -1.99 | -1.26 |
|  | llmg_1349 | orf57 | hypothetical protein, putative integral membrane protein | -1.36 | **-2.48** | -1.38 | 1.14 |
|  | llmg_1355 | orf51 | hypothetical protein | -1.45 | **-2.06** | -1.34 | 1.22 |
|  | llmg_1362 | orf44 | hypothetical protein | 1.13 | **-2.25** | -1.13 | -1.02 |
|  | llmg_1409 | orf3 | hypothetical protein (ATPase/GPTase conserved domains) | 1.69 | **2.16** | 1.41 | 1.11 |
|  | llmg_1411 | orf1 | hypothetical protein | **2.61** | **2.22** | 1.41 | -1.26 |
|  | llmg_1446 |  | hypothetical protein | -1.62 | **-3.16** | **-2.04** | 1.07 |
|  | llmg_1447 |  | hypothetical protein (may be membrane protein) | -1.27 | **-2.10** | -1.62 | -1.32 |
|  | llmg_1507 |  | hypothetical protein | 1.19 | **-2.55** | 1.27 | 1.03 |
|  | llmg_1526 |  | hypothetical protein | -1.31 | **2.19** | -1.24 | -1.21 |
|  | llmg_1650 |  | hypothetical protein | **2.22** |  | **2.27** | -1.66 |
|  | llmg_1659 |  | hypothetical protein | **5.96** | **4.86** | **5.30** | **2.67** |
|  | llmg_1677 |  | hypothetical protein | 1.16 | **2.03** | 1.02 | 1.59 |
|  | llmg_1698 |  | hypothetical protein | 1.17 | 1.39 | 1.23 | **2.76** |
|  | llmg_1912 |  | hypothetical protein | **1.94** | 1.98 | **2.37** | **4.45** |
|  | llmg_1944 |  | hypothetical protein | 1.92 | **2.12** | 1.75 | **2.55** |
|  | llmg_1980 |  | hypothetical protein | -1.52 | -1.71 | **-2.03** | -1.21 |
|  | llmg_1988 |  | hypothetical membrane protein | **-2.65** | -1.83 | **-2.56** | -1.49 |
|  | llmg_2006 |  | hypothetical protein | 1.36 | **2.29** | **2.01** | -1.18 |
|  | llmg_2010 |  | hypothetical protein | **2.23** | 1.14 | **2.10** | 1.14 |
|  | llmg_2041 |  | hypothetical protein | 1.16 | **-2.66** | 1.18 | -1.15 |
|  | llmg_2163 |  | hypothetical protein | 1.23 | 1.88 | **2.11** | 1.34 |
|  | llmg_2164 |  | hypothetical protein | 1.33 | 1.07 | 1.64 | **2.77** |
|  | llmg_2168 |  | hypothetical protein | **2.56** | 1.76 | 1.45 | -1.01 |
|  | llmg_2194 |  | hypothetical protein | 1.21 | **2.38** | 1.74 | -1.61 |
|  | llmg_2207 |  | hypothetical protein | -1.14 | **-2.02** | -1.17 | -1.22 |
|  | llmg_2211 |  | hypothetical protein | -1.88 | -1.37 | **-2.10** | -1.00 |
|  | llmg_2298 |  | hypothetical protein | 1.17 | **2.17** | -1.01 | -1.25 |
|  | llmg_2324 |  | hypothetical protein | **2.49** | **2.32** | 1.62 | 1.34 |
|  | llmg_2342 |  | hypothetical protein | -1.00 | **-2.07** | 1.08 | **2.90** |
|  | llmg_2423 |  | hypothetical protein | 1.24 | 1.88 | 1.23 | **3.96** |
|  | llmg_2431 |  | hypothetical protein | **-3.92** | **-3.28** | **-2.95** | -1.45 |
|  | llmg_2465 |  | hypothetical protein | **2.48** | **2.71** | **2.24** | **2.56** |
|  | llmg_2479 |  | hypothetical protein | -1.51 | **2.21** | 1.10 | 1.20 |
|  | llmg_2515 |  | hypothetical protein | **-2.88** | **-7.78** | **-6.15** | **-2.45** |
|  | llmg_2547 |  | hypothetical protein | -1.99 | **-2.05** | -1.62 | 1.21 |
| **Pseudogenes** | |  |  |  |  |  |  |
|  | llmg_pseudo24 | | pseudogene | -1.03 | 1.03 | 1.08 | **10.05** |
|  | llmg_pseudo29 | | pseudogene | **-2.07** | -1.29 | -1.70 | 1.20 |
|  | llmg_pseudo39 | | pseudogene | 1.26 | **4.04** | **2.04** | -1.04 |
|  | llmg_pseudo40 | | pseudogene | -1.66 | -1.78 | **-2.27** | 1.36 |
|  | llmg_pseudo59 | | pseudogene | **-2.50** | **-3.11** | **-3.87** | -1.22 |
|  | llmg_0396 |  | pseudogene | **2.50** | **5.84** | **5.88** | -1.09 |
