# Supplementary material for: Adaption to glucose limitation is modulated by the pleotropic regulator CcpA, independent of selection pressure strength
Source: BMC Evol Biol. 2019 Jan 10;19:15. doi: 10.1186/s12862-018-1331-x (PMC6327505; doi:10.1186/s12862-018-1331-x)
Supplement: Supplementary file 6 — Table S6. Protein sequence alignment of CcpA from gram positive organisms for which the sequences are available in the public domain. (DOCX 157 kb) [file 12862_2018_1331_MOESM6_ESM.docx]

**Additional file 6: Table S6**.  Protein sequence alignment of CcpA from gram positive organisms for which the sequences are available in the public domain.

|  | | | | | | | | | | | | | | | | | | | | | | | | | | | | | | | | | | | | | | | | | | | | | | | | | | | | | | | |  |  |  |  |  |  |  |  |  |  |  |  |  |  |  |  |  |  |  |  |  |  |  |  |  |  |  |  |  |  |  |  |  |  |  |  |  |  |  |  |  |  |  |  |  |  |  |  |  |  |  |  |  |  |  |  |  |  |  |  |  |  |  |  |  |  |  |  |  |  |  |  |  |  |  |  |  |  |  |  |  |  |  |  |  |  |  |  |  |  |  |  |  |  |  |  |  |  |  |  |  |  |  |  |  |  |  |  |  |  |  |  |  |  |  |  |  |  |  |  |  |  |  |  |  |  |  |  |  |  |  |  |  |  |  |  |  |  |  |  |  |  |  |  |  |  |  |  |  |  |  |  |  |  |  |  |  |  |  |  |  |  |  |  |  |  |  |  |  |  |  |  |  |  |  |  |  |  |  |  |  |  |  |  |  |  |  |  |  |  |  |  |  |  |  |  |  |  |  |  |  |  |  |  |  |  |  |  |  |  |  |  |  |  |  |  |  |  |  |  |  |  |  |  |  |  |  |  |  |  |  |  |  |  |  |  |  |  |  |  |  |  |  |  |  |  |  |  |  |  |  |  |  |  |  |  |  |  |  |  |  |  |  |  |  |  |
| --- | --- | --- | --- | --- | --- | --- | --- | --- | --- | --- | --- | --- | --- | --- | --- | --- | --- | --- | --- | --- | --- | --- | --- | --- | --- | --- | --- | --- | --- | --- | --- | --- | --- | --- | --- | --- | --- | --- | --- | --- | --- | --- | --- | --- | --- | --- | --- | --- | --- | --- | --- | --- | --- | --- | --- | --- | --- | --- | --- | --- | --- | --- | --- | --- | --- | --- | --- | --- | --- | --- | --- | --- | --- | --- | --- | --- | --- | --- | --- | --- | --- | --- | --- | --- | --- | --- | --- | --- | --- | --- | --- | --- | --- | --- | --- | --- | --- | --- | --- | --- | --- | --- | --- | --- | --- | --- | --- | --- | --- | --- | --- | --- | --- | --- | --- | --- | --- | --- | --- | --- | --- | --- | --- | --- | --- | --- | --- | --- | --- | --- | --- | --- | --- | --- | --- | --- | --- | --- | --- | --- | --- | --- | --- | --- | --- | --- | --- | --- | --- | --- | --- | --- | --- | --- | --- | --- | --- | --- | --- | --- | --- | --- | --- | --- | --- | --- | --- | --- | --- | --- | --- | --- | --- | --- | --- | --- | --- | --- | --- | --- | --- | --- | --- | --- | --- | --- | --- | --- | --- | --- | --- | --- | --- | --- | --- | --- | --- | --- | --- | --- | --- | --- | --- | --- | --- | --- | --- | --- | --- | --- | --- | --- | --- | --- | --- | --- | --- | --- | --- | --- | --- | --- | --- | --- | --- | --- | --- | --- | --- | --- | --- | --- | --- | --- | --- | --- | --- | --- | --- | --- | --- | --- | --- | --- | --- | --- | --- | --- | --- | --- | --- | --- | --- | --- | --- | --- | --- | --- | --- | --- | --- | --- | --- | --- | --- | --- | --- | --- | --- | --- | --- | --- | --- | --- | --- | --- | --- | --- | --- | --- | --- | --- | --- | --- | --- | --- | --- | --- | --- | --- | --- | --- | --- | --- | --- | --- | --- | --- | --- | --- | --- | --- | --- | --- | --- | --- | --- | --- | --- | --- | --- | --- | --- | --- | --- | --- | --- | --- | --- | --- | --- |
|  |  |  |  |  |  |  |  |  |  | 10 |  |  |  |  |  |  |  |  | 20 |  |  |  |  |  |  |  |  | 30 |  |  |  |  |  |  |  | 40 |  |  |  |  |  |  |  |  | 50 |  |  |  |  |  |  |  |  | 60 |  | 7080 | | | | | | | | | | | | | | | | | | | | | | | | | | | | | | | | | | | | | | | | | | | | | | | | | | | | | | | | | | | | | | | | | | | | | | | | | | | | | | | | | | | | | | | | | | | | | | | | | | | | | | | | | | | | | | | | | | | | | | | | | | | | | | | | | | | | | | | | | | | | | | | | | | | | | | | | | | | | | | | | | | | | | | | | | | | | | | | | | | | | | | | | | | | | | | | | | | | | | | | | | | | | | | | | | | | | | | | | | | | | | | | | | | | | | | | | | | | | | | | | | | | | | | | | | | | | | | | | | | | | | |  |  |  |  |
| *Bacillus amyloliquefaciens* | | | | | | | | | | | | | | | | | | | | | | | | | | | | | | | | | | | | | | | | | | | | | | | | | | | | | | | | | - | | - | | - | | M | | S | | N | | I | | | T | | | I | | | Y | | | D | | | V | | | A | | | | R | | | E | | | A | | | N | | | | V | | | | S | | | | **M** | | | | | A | | | | T | | | | | | | V | | | | | | | | | | | | | | | | | | | | | S | | | | | | | | | | | | | | | | | | R | | | | | | | | | V | | | | V | | | | N | | | | G | | | | N | | | | P | | | | N | | | V | | | | K | | | | P | | | | T | | | | T | | R | | | | K | | | K | | | V | | | | L | | | | | E | | | | A | | | I | | | | D | | | | R | | | | L | | | G | | | | Y | | | | R | | | | P | | | N | | | | A | | | | V | | | | A | | | | R | | | | G | | | L | | | A | | | S | | KKTTTVGVIIPDISSIFYS | | | | | | | | | | |  |  |  |
| *Bacillus anthracis* | | | | | | | | | | | | | | | | | | | | | | | | | | | | | | | | | | | | | | | | | | | | | | | | | | | | | | | | | - | | - | | - | | - | | M | | N | | V | | | T | | | I | | | Y | | | D | | | V | | | A | | | | R | | | E | | | A | | | N | | | | V | | | | S | | | | **M** | | | | | A | | | | T | | | | | | | V | | | | | | | | | | | | | | | | | | | | | S | | | | | | | | | | | | | | | | | | R | | | | | | | | | V | | | | V | | | | N | | | | G | | | | N | | | | P | | | | N | | | V | | | | K | | | | P | | | | T | | | | T | | R | | | | K | | | K | | | V | | | | L | | | | | E | | | | A | | | I | | | | D | | | | R | | | | L | | | G | | | | Y | | | | R | | | | P | | | N | | | | A | | | | V | | | | A | | | | R | | | | G | | | L | | | A | | | S | | KKTTTVGVIIPDISNTFYA | | | | | | | | | | |  |  |  |
| *Bacillus licheniformis* | | | | | | | | | | | | | | | | | | | | | | | | | | | | | | | | | | | | | | | | | | | | | | | | | | | | | | | | | - | | - | | - | | M | | S | | N | | V | | | T | | | I | | | Y | | | D | | | V | | | A | | | | R | | | E | | | A | | | N | | | | V | | | | S | | | | **M** | | | | | A | | | | T | | | | | | | V | | | | | | | | | | | | | | | | | | | | | S | | | | | | | | | | | | | | | | | | R | | | | | | | | | V | | | | V | | | | N | | | | G | | | | N | | | | P | | | | N | | | V | | | | K | | | | P | | | | T | | | | T | | R | | | | K | | | K | | | V | | | | L | | | | | E | | | | A | | | I | | | | E | | | | R | | | | L | | | G | | | | Y | | | | R | | | | P | | | N | | | | A | | | | V | | | | A | | | | R | | | | G | | | L | | | A | | | S | | KKTTTVGVIIPDISSIFYS | | | | | | | | | | |  |  |  |
| *Bacillus megaterium* | | | | | | | | | | | | | | | | | | | | | | | | | | | | | | | | | | | | | | | | | | | | | | | | | | | | | | | | | - | | - | | - | | - | | M | | N | | V | | | T | | | I | | | Y | | | D | | | V | | | A | | | | R | | | E | | | A | | | S | | | | V | | | | S | | | | **M** | | | | | A | | | | T | | | | | | | V | | | | | | | | | | | | | | | | | | | | | S | | | | | | | | | | | | | | | | | | R | | | | | | | | | V | | | | V | | | | N | | | | G | | | | N | | | | P | | | | N | | | V | | | | K | | | | P | | | | S | | | | T | | R | | | | K | | | K | | | V | | | | L | | | | | E | | | | T | | | I | | | | E | | | | R | | | | L | | | G | | | | Y | | | | R | | | | P | | | N | | | | A | | | | V | | | | A | | | | R | | | | G | | | L | | | A | | | S | | KKTTTVGVIIPDISNIFYA | | | | | | | | | | |  |  |  |
| *Bacillus subtilis* | | | | | | | | | | | | | | | | | | | | | | | | | | | | | | | | | | | | | | | | | | | | | | | | | | | | | | | | | - | | - | | - | | M | | S | | N | | I | | | T | | | I | | | Y | | | D | | | V | | | A | | | | R | | | E | | | A | | | N | | | | V | | | | S | | | | **M** | | | | | A | | | | T | | | | | | | V | | | | | | | | | | | | | | | | | | | | | S | | | | | | | | | | | | | | | | | | R | | | | | | | | | V | | | | V | | | | N | | | | G | | | | N | | | | P | | | | N | | | V | | | | K | | | | P | | | | T | | | | T | | R | | | | K | | | K | | | V | | | | L | | | | | E | | | | A | | | I | | | | E | | | | R | | | | L | | | G | | | | Y | | | | R | | | | P | | | N | | | | A | | | | V | | | | A | | | | R | | | | G | | | L | | | A | | | S | | KKTTTVGVIIPDISSIFYS | | | | | | | | | | |  |  |  |
| *Enterococcus faecalis* | | | | | | | | | | | | | | | | | | | | | | | | | | | | | | | | | | | | | | | | | | | | | | | | | | | | | | | | | - | | M | | E | | K | | Q | | T | | I | | | T | | | I | | | Y | | | D | | | V | | | A | | | | R | | | E | | | A | | | N | | | | V | | | | S | | | | **M** | | | | | A | | | | T | | | | | | | V | | | | | | | | | | | | | | | | | | | | | S | | | | | | | | | | | | | | | | | | R | | | | | | | | | V | | | | V | | | | N | | | | G | | | | N | | | | P | | | | N | | | V | | | | K | | | | P | | | | A | | | | T | | R | | | | K | | | K | | | V | | | | L | | | | | E | | | | V | | | I | | | | D | | | | R | | | | L | | | D | | | | Y | | | | R | | | | P | | | N | | | | A | | | | V | | | | A | | | | R | | | | G | | | L | | | A | | | S | | KKTTTVGVIIPDVSNAFFA | | | | | | | | | | |  |  |  |
| *Lactobacillus. casei*subsp.*casei* | | | | | | | | | | | | | | | | | | | | | | | | | | | | | | | | | | | | | | | | | | | | | | | | | | | | | | | | | - | | M | | E | | K | | Q | | T | | I | | | T | | | I | | | Y | | | D | | | V | | | A | | | | R | | | E | | | A | | | N | | | | V | | | | S | | | | **M** | | | | | A | | | | T | | | | | | | V | | | | | | | | | | | | | | | | | | | | | S | | | | | | | | | | | | | | | | | | R | | | | | | | | | V | | | | V | | | | N | | | | G | | | | N | | | | P | | | | N | | | V | | | | K | | | | P | | | | A | | | | T | | R | | | | K | | | K | | | V | | | | L | | | | | E | | | | V | | | I | | | | E | | | | R | | | | L | | | D | | | | Y | | | | R | | | | P | | | N | | | | A | | | | V | | | | A | | | | R | | | | G | | | L | | | A | | | S | | KKTTTVGVIIPDVTNMFFS | | | | | | | | | | |  |  |  |
| *Lactobacillus delbrueckii*subsp.*bulgaricus* | | | | | | | | | | | | | | | | | | | | | | | | | | | | | | | | | | | | | | | | | | | | | | | | | | | | | | | | | - | | M | | N | | K | | Q | | D | | V | | | T | | | I | | | Y | | | D | | | V | | | A | | | | R | | | E | | | A | | | K | | | | V | | | | S | | | | **M** | | | | | A | | | | T | | | | | | | V | | | | | | | | | | | | | | | | | | | | | S | | | | | | | | | | | | | | | | | | R | | | | | | | | | V | | | | V | | | | N | | | | G | | | | N | | | | N | | | | N | | | V | | | | R | | | | K | | | | E | | | | T | | R | | | | D | | | R | | | V | | | | M | | | | | E | | | | V | | | I | | | | K | | | | R | | | | L | | | H | | | | Y | | | | Q | | | | P | | | N | | | | A | | | | V | | | | A | | | | Q | | | | G | | | L | | | A | | | S | | KRTTTVGLIVPDLTNLYFA | | | | | | | | | | |  |  |  |
| *Lactobacillus pentosus* | | | | | | | | | | | | | | | | | | | | | | | | | | | | | | | | | | | | | | | | | | | | | | | | | | | | | | | | | - | | M | | E | | K | | Q | | T | | V | | | T | | | I | | | Y | | | D | | | V | | | A | | | | R | | | E | | | A | | | A | | | | V | | | | S | | | | **M** | | | | | A | | | | T | | | | | | | V | | | | | | | | | | | | | | | | | | | | | S | | | | | | | | | | | | | | | | | | R | | | | | | | | | V | | | | V | | | | N | | | | G | | | | N | | | | P | | | | N | | | V | | | | K | | | | P | | | | A | | | | T | | R | | | | K | | | K | | | V | | | | L | | | | | A | | | | V | | | I | | | | E | | | | R | | | | L | | | D | | | | Y | | | | R | | | | P | | | N | | | | A | | | | V | | | | A | | | | R | | | | G | | | L | | | A | | | S | | KRSTTVGVIIPDVTNIYFA | | | | | | | | | | |  |  |  |
| *Lactobacillus plantarum* | | | | | | | | | | | | | | | | | | | | | | | | | | | | | | | | | | | | | | | | | | | | | | | | | | | | | | | | | - | | M | | E | | K | | Q | | T | | V | | | T | | | I | | | Y | | | D | | | V | | | A | | | | A | | | E | | | A | | | A | | | | V | | | | S | | | | **M** | | | | | A | | | | T | | | | | | | V | | | | | | | | | | | | | | | | | | | | | S | | | | | | | | | | | | | | | | | | R | | | | | | | | | V | | | | V | | | | N | | | | G | | | | N | | | | P | | | | N | | | V | | | | K | | | | P | | | | A | | | | T | | A | | | | K | | | K | | | V | | | | L | | | | | A | | | | V | | | I | | | | E | | | | R | | | | L | | | D | | | | Y | | | | R | | | | P | | | N | | | | A | | | | V | | | | A | | | | R | | | | G | | | L | | | A | | | S | | KRSTTVGVIIPDVTNIYFA | | | | | | | | | | |  |  |  |
| *Lactobacillus reuteri* | | | | | | | | | | | | | | | | | | | | | | | | | | | | | | | | | | | | | | | | | | | | | | | | | | | | | | | | | - | | M | | E | | K | | Q | | S | | V | | | T | | | I | | | Y | | | T | | | V | | | A | | | | R | | | E | | | A | | | R | | | | V | | | | S | | | | **M** | | | | | A | | | | T | | | | | | | V | | | | | | | | | | | | | | | | | | | | | S | | | | | | | | | | | | | | | | | | R | | | | | | | | | V | | | | V | | | | N | | | | G | | | | N | | | | P | | | | N | | | V | | | | K | | | | P | | | | E | | | | T | | R | | | | Q | | | K | | | V | | | | L | | | | | D | | | | V | | | I | | | | K | | | | Q | | | | L | | | N | | | | Y | | | | R | | | | P | | | N | | | | A | | | | V | | | | A | | | | R | | | | G | | | L | | | A | | | S | | KKTTTVGVVIPNITDPYFA | | | | | | | | | | |  |  |  |
| *Lactobacillus sakei*subsp. *sakei* | | | | | | | | | | | | | | | | | | | | | | | | | | | | | | | | | | | | | | | | | | | | | | | | | | | | | | | | | - | | M | | E | | K | | Q | | T | | I | | | T | | | I | | | Y | | | D | | | V | | | A | | | | R | | | E | | | A | | | D | | | | V | | | | S | | | | **M** | | | | | A | | | | T | | | | | | | V | | | | | | | | | | | | | | | | | | | | | S | | | | | | | | | | | | | | | | | | R | | | | | | | | | V | | | | V | | | | N | | | | G | | | | N | | | | P | | | | N | | | V | | | | K | | | | P | | | | A | | | | T | | R | | | | K | | | K | | | V | | | | L | | | | | E | | | | V | | | I | | | | D | | | | R | | | | L | | | D | | | | Y | | | | R | | | | P | | | N | | | | A | | | | V | | | | A | | | | R | | | | G | | | L | | | A | | | S | | KKTTTVGVIIPDVSDIYFA | | | | | | | | | | |  |  |  |
| *Lactococcus lactis*AM2 | | | | | | | | | | | | | | | | | | | | | | | | | | | | | | | | | | | | | | | | | | | | | | | | | | | | | | | | | - | | M | | V | | E | | S | | - | | T | | | T | | | I | | | Y | | | D | | | V | | | A | | | | R | | | V | | | A | | | G | | | | V | | | | S | | | | **M** | | | | | A | | | | T | | | | | | | V | | | | | | | | | | | | | | | | | | | | | S | | | | | | | | | | | | | | | | | | R | | | | | | | | | V | | | | V | | | | N | | | | G | | | | N | | | | A | | | | N | | | V | | | | K | | | | E | | | | K | | | | T | | R | | | | Q | | | K | | | V | | | | L | | | | | E | | | | A | | | I | | | | A | | | | E | | | | L | | | D | | | | Y | | | | R | | | | P | | | N | | | | A | | | | V | | | | A | | | | R | | | | G | | | L | | | A | | | S | | KRTTTVGVILPTITSTYFA | | | | | | | | | | |  |  |  |
| *Lactococcus lactis*LMG9447 | | | | | | | | | | | | | | | | | | | | | | | | | | | | | | | | | | | | | | | | | | | | | | | | | | | | | | | | | - | | M | | V | | E | | S | | T | | T | | | T | | | I | | | Y | | | D | | | V | | | A | | | | R | | | V | | | A | | | G | | | | V | | | | S | | | | **M** | | | | | A | | | | T | | | | | | | V | | | | | | | | | | | | | | | | | | | | | S | | | | | | | | | | | | | | | | | | R | | | | | | | | | V | | | | V | | | | N | | | | G | | | | N | | | | A | | | | N | | | V | | | | K | | | | E | | | | K | | | | T | | R | | | | Q | | | K | | | V | | | | L | | | | | E | | | | A | | | I | | | | A | | | | E | | | | L | | | D | | | | Y | | | | R | | | | P | | | N | | | | A | | | | V | | | | A | | | | R | | | | G | | | L | | | A | | | S | | KRTTTVGVILPTITSTYFA | | | | | | | | | | |  |  |  |
| *Lactococcus lactis*P7304 | | | | | | | | | | | | | | | | | | | | | | | | | | | | | | | | | | | | | | | | | | | | | | | | | | | | | | | | | - | | M | | V | | E | | S | | T | | T | | | T | | | I | | | Y | | | D | | | V | | | A | | | | R | | | V | | | A | | | G | | | | V | | | | S | | | | **T** | | | | | A | | | | T | | | | | | | V | | | | | | | | | | | | | | | | | | | | | S | | | | | | | | | | | | | | | | | | R | | | | | | | | | V | | | | V | | | | N | | | | G | | | | N | | | | A | | | | N | | | V | | | | K | | | | E | | | | K | | | | T | | R | | | | Q | | | K | | | V | | | | L | | | | | E | | | | A | | | I | | | | A | | | | E | | | | L | | | D | | | | Y | | | | R | | | | P | | | N | | | | A | | | | V | | | | A | | | | R | | | | G | | | L | | | A | | | S | | KRTTTVGVILPTITSTYFA | | | | | | | | | | |  |  |  |
| *Lactococcus lactis*subsp.*cremoris*MG1363 | | | | | | | | | | | | | | | | | | | | | | | | | | | | | | | | | | | | | | | | | | | | | | | | | | | | | | | | | - | | M | | V | | E | | S | | T | | T | | | T | | | I | | | Y | | | D | | | V | | | A | | | | R | | | V | | | A | | | G | | | | V | | | | S | | | | **M** | | | | | A | | | | T | | | | | | | V | | | | | | | | | | | | | | | | | | | | | S | | | | | | | | | | | | | | | | | | R | | | | | | | | | V | | | | V | | | | N | | | | G | | | | N | | | | A | | | | N | | | V | | | | K | | | | E | | | | K | | | | T | | R | | | | Q | | | K | | | V | | | | L | | | | | E | | | | A | | | I | | | | A | | | | E | | | | L | | | D | | | | Y | | | | R | | | | P | | | N | | | | A | | | | V | | | | A | | | | R | | | | G | | | L | | | A | | | S | | KRTTTVGVILPTITSTYFA | | | | | | | | | | |  |  |  |
| *Lactococcus lactis*subsp.*lactis*Il1403 | | | | | | | | | | | | | | | | | | | | | | | | | | | | | | | | | | | | | | | | | | | | | | | | | | | | | | | | | - | | M | | V | | E | | S | | T | | T | | | T | | | I | | | Y | | | D | | | V | | | A | | | | R | | | V | | | A | | | G | | | | V | | | | S | | | | **M** | | | | | A | | | | T | | | | | | | V | | | | | | | | | | | | | | | | | | | | | S | | | | | | | | | | | | | | | | | | R | | | | | | | | | V | | | | V | | | | N | | | | G | | | | N | | | | A | | | | N | | | V | | | | K | | | | E | | | | K | | | | T | | R | | | | Q | | | K | | | V | | | | L | | | | | E | | | | A | | | I | | | | A | | | | E | | | | L | | | D | | | | Y | | | | R | | | | P | | | N | | | | A | | | | V | | | | A | | | | R | | | | G | | | L | | | A | | | S | | KRTTTVGVILPTITSTYFA | | | | | | | | | | |  |  |  |
| *Lactococcus lactis*subsp.*lactis*KF147 | | | | | | | | | | | | | | | | | | | | | | | | | | | | | | | | | | | | | | | | | | | | | | | | | | | | | | | | | - | | M | | V | | E | | S | | T | | T | | | T | | | I | | | Y | | | D | | | V | | | A | | | | R | | | V | | | A | | | G | | | | V | | | | S | | | | **M** | | | | | A | | | | T | | | | | | | V | | | | | | | | | | | | | | | | | | | | | S | | | | | | | | | | | | | | | | | | R | | | | | | | | | V | | | | V | | | | N | | | | G | | | | N | | | | A | | | | N | | | V | | | | K | | | | E | | | | K | | | | T | | R | | | | Q | | | K | | | V | | | | L | | | | | E | | | | A | | | I | | | | A | | | | E | | | | L | | | D | | | | Y | | | | R | | | | P | | | N | | | | A | | | | V | | | | A | | | | R | | | | G | | | L | | | A | | | S | | KRTTTVGVILPTITSTYFA | | | | | | | | | | |  |  |  |
| *Lactococcus lactis*UC317 | | | | | | | | | | | | | | | | | | | | | | | | | | | | | | | | | | | | | | | | | | | | | | | | | | | | | | | | | - | | M | | V | | E | | S | | T | | T | | | T | | | I | | | Y | | | D | | | V | | | A | | | | R | | | V | | | A | | | G | | | | V | | | | S | | | | **M** | | | | | A | | | | T | | | | | | | V | | | | | | | | | | | | | | | | | | | | | S | | | | | | | | | | | | | | | | | | R | | | | | | | | | V | | | | V | | | | N | | | | G | | | | N | | | | A | | | | N | | | V | | | | K | | | | E | | | | K | | | | T | | R | | | | Q | | | K | | | V | | | | L | | | | | E | | | | A | | | I | | | | A | | | | E | | | | L | | | D | | | | Y | | | | R | | | | P | | | N | | | | A | | | | V | | | | A | | | | R | | | | G | | | L | | | A | | | S | | KRTTTVGVILPTITSTYFA | | | | | | | | | | |  |  |  |
| *Listeria monocytogenes* | | | | | | | | | | | | | | | | | | | | | | | | | | | | | | | | | | | | | | | | | | | | | | | | | | | | | | | | | - | | - | | - | | - | | M | | N | | V | | | T | | | I | | | Y | | | D | | | V | | | A | | | | R | | | E | | | A | | | N | | | | V | | | | S | | | | **M** | | | | | A | | | | T | | | | | | | V | | | | | | | | | | | | | | | | | | | | | S | | | | | | | | | | | | | | | | | | R | | | | | | | | | V | | | | V | | | | N | | | | G | | | | N | | | | P | | | | N | | | V | | | | K | | | | P | | | | V | | | | T | | R | | | | K | | | K | | | V | | | | L | | | | | D | | | | V | | | I | | | | N | | | | Q | | | | L | | | G | | | | Y | | | | R | | | | P | | | N | | | | A | | | | V | | | | A | | | | R | | | | G | | | L | | | A | | | S | | KRTTTVGVIIPDISNVFYA | | | | | | | | | | |  |  |  |
| *Paenibacillus mucilaginosus* | | | | | | | | | | | | | | | | | | | | | | | | | | | | | | | | | | | | | | | | | | | | | | | | | | | | | | | | | - | | - | | - | | - | | - | | - | | - | | | - | | | - | | | - | | | - | | | - | | | - | | | | - | | | - | | | - | | | - | | | | - | | | | - | | | | **M** | | | | | A | | | | T | | | | | | | V | | | | | | | | | | | | | | | | | | | | | S | | | | | | | | | | | | | | | | | | R | | | | | | | | | V | | | | V | | | | N | | | | N | | | | N | | | | P | | | | N | | | V | | | | K | | | | P | | | | Q | | | | T | | R | | | | K | | | K | | | V | | | | F | | | | | E | | | | A | | | I | | | | E | | | | R | | | | L | | | G | | | | Y | | | | R | | | | P | | | N | | | | A | | | | V | | | | A | | | | R | | | | G | | | L | | | A | | | S | | KKTTTVGVVIPDISNSIFS | | | | | | | | | | |  |  |  |
| *Staphalococcus aureus*subsp.*aureus* | | | | | | | | | | | | | | | | | | | | | | | | | | | | | | | | | | | | | | | | | | | | | | | | | | | | | | | | | - | | - | | - | | - | | M | | T | | V | | | T | | | I | | | Y | | | D | | | V | | | A | | | | R | | | E | | | A | | | R | | | | V | | | | S | | | | **M** | | | | | A | | | | T | | | | | | | V | | | | | | | | | | | | | | | | | | | | | S | | | | | | | | | | | | | | | | | | R | | | | | | | | | V | | | | V | | | | N | | | | G | | | | N | | | | Q | | | | N | | | V | | | | K | | | | A | | | | E | | | | T | | K | | | | N | | | K | | | V | | | | N | | | | | E | | | | V | | | I | | | | K | | | | R | | | | L | | | N | | | | Y | | | | R | | | | P | | | N | | | | A | | | | V | | | | A | | | | R | | | | G | | | L | | | A | | | S | | KKTTTVGVIIPDISNIYYS | | | | | | | | | | |  |  |  |
| *Staphalococcus xylosus* | | | | | | | | | | | | | | | | | | | | | | | | | | | | | | | | | | | | | | | | | | | | | | | | | | | | | | | | | - | | - | | - | | - | | M | | T | | V | | | T | | | I | | | Y | | | D | | | V | | | A | | | | R | | | E | | | A | | | R | | | | V | | | | S | | | | **M** | | | | | A | | | | T | | | | | | | V | | | | | | | | | | | | | | | | | | | | | S | | | | | | | | | | | | | | | | | | R | | | | | | | | | V | | | | V | | | | N | | | | G | | | | N | | | | Q | | | | N | | | V | | | | K | | | | P | | | | E | | | | T | | R | | | | D | | | K | | | V | | | | N | | | | | E | | | | V | | | I | | | | K | | | | K | | | | L | | | N | | | | Y | | | | R | | | | P | | | N | | | | A | | | | V | | | | A | | | | R | | | | G | | | L | | | A | | | S | | KRTTTVGVIIPDISNVYYS | | | | | | | | | | |  |  |  |
| *Staphalococcus bovis* | | | | | | | | | | | | | | | | | | | | | | | | | | | | | | | | | | | | | | | | | | | | | | | | | | | | | | | | | M | | N | | T | | D | | D | | T | | I | | | T | | | I | | | Y | | | D | | | V | | | A | | | | R | | | E | | | A | | | G | | | | V | | | | S | | | | **M** | | | | | A | | | | T | | | | | | | V | | | | | | | | | | | | | | | | | | | | | S | | | | | | | | | | | | | | | | | | R | | | | | | | | | V | | | | V | | | | N | | | | G | | | | N | | | | K | | | | N | | | V | | | | K | | | | E | | | | N | | | | T | | R | | | | K | | | K | | | V | | | | L | | | | | E | | | | V | | | I | | | | D | | | | R | | | | L | | | D | | | | Y | | | | R | | | | P | | | N | | | | A | | | | V | | | | A | | | | R | | | | G | | | L | | | A | | | S | | KKTTTVGVVIPNIANSYFS | | | | | | | | | | |  |  |  |
| *Streptococcus equi*subsp. *zooepidemicus* | | | | | | | | | | | | | | | | | | | | | | | | | | | | | | | | | | | | | | | | | | | | | | | | | | | | | | | | | M | | N | | T | | D | | D | | T | | I | | | T | | | I | | | Y | | | D | | | V | | | A | | | | R | | | E | | | A | | | G | | | | V | | | | S | | | | **M** | | | | | A | | | | T | | | | | | | V | | | | | | | | | | | | | | | | | | | | | S | | | | | | | | | | | | | | | | | | R | | | | | | | | | V | | | | V | | | | N | | | | G | | | | N | | | | K | | | | N | | | V | | | | K | | | | E | | | | N | | | | T | | R | | | | K | | | K | | | V | | | | L | | | | | E | | | | V | | | I | | | | D | | | | R | | | | L | | | D | | | | Y | | | | R | | | | P | | | N | | | | A | | | | V | | | | A | | | | R | | | | G | | | L | | | A | | | S | | KKTTTVGVVIPNIANSYFS | | | | | | | | | | |  |  |  |
| *Streptococcus gallolyticus* | | | | | | | | | | | | | | | | | | | | | | | | | | | | | | | | | | | | | | | | | | | | | | | | | | | | | | | | | M | | N | | T | | D | | D | | T | | I | | | T | | | I | | | Y | | | D | | | V | | | A | | | | R | | | E | | | A | | | G | | | | V | | | | S | | | | **M** | | | | | A | | | | T | | | | | | | V | | | | | | | | | | | | | | | | | | | | | S | | | | | | | | | | | | | | | | | | R | | | | | | | | | V | | | | V | | | | N | | | | G | | | | N | | | | K | | | | N | | | V | | | | K | | | | E | | | | N | | | | T | | R | | | | K | | | K | | | V | | | | L | | | | | E | | | | V | | | I | | | | D | | | | R | | | | L | | | D | | | | Y | | | | R | | | | P | | | N | | | | A | | | | V | | | | A | | | | R | | | | G | | | L | | | A | | | S | | KKTTTVGVVIPNIANSYFS | | | | | | | | | | |  |  |  |
| *Streptococcus mutans* | | | | | | | | | | | | | | | | | | | | | | | | | | | | | | | | | | | | | | | | | | | | | | | | | | | | | | | | | M | | N | | T | | D | | D | | T | | I | | | T | | | I | | | Y | | | D | | | V | | | A | | | | R | | | E | | | A | | | G | | | | V | | | | S | | | | **M** | | | | | A | | | | T | | | | | | | V | | | | | | | | | | | | | | | | | | | | | S | | | | | | | | | | | | | | | | | | R | | | | | | | | | V | | | | V | | | | N | | | | G | | | | N | | | | K | | | | N | | | V | | | | K | | | | E | | | | N | | | | T | | R | | | | K | | | K | | | V | | | | L | | | | | E | | | | V | | | I | | | | D | | | | R | | | | L | | | D | | | | Y | | | | R | | | | P | | | N | | | | A | | | | V | | | | A | | | | R | | | | G | | | L | | | A | | | S | | KKTTTVGVVIPNIANAYFS | | | | | | | | | | |  |  |  |
| *Streptococcus thermophilus* | | | | | | | | | | | | | | | | | | | | | | | | | | | | | | | | | | | | | | | | | | | | | | | | | | | | | | | | | M | | N | | T | | D | | E | | T | | I | | | T | | | I | | | Y | | | D | | | V | | | A | | | | R | | | E | | | A | | | G | | | | V | | | | S | | | | **M** | | | | | A | | | | T | | | | | | | V | | | | | | | | | | | | | | | | | | | | | S | | | | | | | | | | | | | | | | | | R | | | | | | | | | V | | | | V | | | | N | | | | G | | | | N | | | | K | | | | N | | | V | | | | K | | | | E | | | | N | | | | T | | R | | | | K | | | K | | | V | | | | L | | | | | E | | | | V | | | I | | | | D | | | | R | | | | L | | | D | | | | Y | | | | R | | | | P | | | N | | | | A | | | | V | | | | A | | | | R | | | | G | | | L | | | A | | | S | | KKTTTVGVVIPNIVNSYFA | | | | | | | | | | |  |  |  |
|  | | | | | | | | | | | | | | | | | | | | | | | | | | | | | | | | | | | | | | | | | | | | | | | | | | | | | | | | | | | | | | | | | | | | | | | | | | | | | | | | | | | | | | | | | | | | | | | | | | | | | | | | | | | | | | | | | | | | | | | | | | | | | | | | | | | | | | | | | | | | | | | | | | | | | | | | | | | | | | | | | | | | | | | | | | | | | | | | | | | | | | | | | | | | | | | | | | | | | | | | | | | | | | | | | | | | | | | | | | | | | | | | | | | | | | | | | | | | | | | | | | | | | | | | | | | | | | | | | | | | | | | | | | | | | | | | | | | | | | | | | | | | | | | | | | | | | | | | | | | | | | | | | | | |  | | | | | | | | | | |  |  |  |
| *Bacillus amyloliquefaciens* | | | | | | | | | | | | | | | | | | | | | | | | | | | | | | | | | | | | | | | | | | | | | | | | | | | | | | | | | E | L | | A | | R | | G | | I | | E | | D | | | I | | | A | | | T | | | | | M | | | | Y | | | | K | | | Y | | | N | | | | I | | | | I | | | | L | | | | | S | | | | N | | | | S | | | | | | | | | | | | D | | | | | | | | | | | | | | | | | | | | | Q | | | | | | | | | | | | | | | | | | N | | | | L | | | | E | | | | | K | | | | E | | L | | | | | H | | | | L | | | | L | | | N | | | | T | | | | M | | | | | L | | | | | G | | | K | | | Q | | | | V | | | | D | | | | G | | | | I | | | | V | | | | F | | | M | | | | | G | | | | G | | | | N | | | | | I | | | | T | | | | D | | | | E | | | | H | | | | V | | | E | | | E | | | F | | | K | | | R | | | SPVPIVLAASVEEQGETPS | | | |  |  |
| *Bacillus anthracis* | | | | | | | | | | | | | | | | | | | | | | | | | | | | | | | | | | | | | | | | | | | | | | | | | | | | | | | | | E | L | | A | | R | | G | | I | | E | | D | | | I | | | A | | | T | | | | | M | | | | Y | | | | K | | | Y | | | N | | | | I | | | | I | | | | L | | | | | S | | | | N | | | | S | | | | | | | | | | | | D | | | | | | | | | | | | | | | | | | | | | Q | | | | | | | | | | | | | | | | | | N | | | | K | | | | E | | | | | K | | | | E | | F | | | | | H | | | | L | | | | L | | | N | | | | T | | | | M | | | | | L | | | | | G | | | K | | | Q | | | | V | | | | D | | | | G | | | | I | | | | V | | | | F | | | M | | | | | G | | | | E | | | | D | | | | | I | | | | T | | | | D | | | | I | | | | H | | | | I | | | E | | | E | | | F | | | K | | | K | | | SPVPIVLAASFDEQNETPS | | | |  |  |
| *Bacillus licheniformis* | | | | | | | | | | | | | | | | | | | | | | | | | | | | | | | | | | | | | | | | | | | | | | | | | | | | | | | | | E | L | | A | | R | | G | | I | | E | | D | | | I | | | A | | | T | | | | | M | | | | Y | | | | K | | | Y | | | N | | | | I | | | | I | | | | L | | | | | S | | | | N | | | | S | | | | | | | | | | | | D | | | | | | | | | | | | | | | | | | | | | Q | | | | | | | | | | | | | | | | | | N | | | | M | | | | D | | | | | K | | | | E | | L | | | | | H | | | | L | | | | L | | | N | | | | T | | | | M | | | | | L | | | | | G | | | K | | | Q | | | | V | | | | D | | | | G | | | | I | | | | V | | | | F | | | M | | | | | S | | | | G | | | | N | | | | | V | | | | T | | | | E | | | | E | | | | H | | | | V | | | E | | | E | | | F | | | K | | | R | | | SPVPIVLAASVEEKGETPS | | | |  |  |
| *Bacillus megaterium* | | | | | | | | | | | | | | | | | | | | | | | | | | | | | | | | | | | | | | | | | | | | | | | | | | | | | | | | | E | L | | A | | R | | G | | I | | E | | D | | | I | | | A | | | T | | | | | M | | | | Y | | | | K | | | Y | | | N | | | | I | | | | I | | | | L | | | | | S | | | | N | | | | S | | | | | | | | | | | | D | | | | | | | | | | | | | | | | | | | | | Q | | | | | | | | | | | | | | | | | | N | | | | Q | | | | D | | | | | K | | | | E | | L | | | | | H | | | | L | | | | L | | | N | | | | N | | | | M | | | | | L | | | | | G | | | K | | | Q | | | | V | | | | D | | | | G | | | | I | | | | I | | | | F | | | M | | | | | S | | | | G | | | | N | | | | | V | | | | T | | | | E | | | | E | | | | H | | | | V | | | E | | | E | | | L | | | K | | | K | | | SPVPVVLAASIESTNQIPS | | | |  |  |
| *Bacillus subtilis* | | | | | | | | | | | | | | | | | | | | | | | | | | | | | | | | | | | | | | | | | | | | | | | | | | | | | | | | | E | L | | A | | R | | G | | I | | E | | D | | | I | | | A | | | T | | | | | M | | | | Y | | | | K | | | Y | | | N | | | | I | | | | I | | | | L | | | | | S | | | | N | | | | S | | | | | | | | | | | | D | | | | | | | | | | | | | | | | | | | | | Q | | | | | | | | | | | | | | | | | | N | | | | M | | | | E | | | | | K | | | | E | | L | | | | | H | | | | L | | | | L | | | N | | | | T | | | | M | | | | | L | | | | | G | | | K | | | Q | | | | V | | | | D | | | | G | | | | I | | | | V | | | | F | | | M | | | | | G | | | | G | | | | N | | | | | I | | | | T | | | | D | | | | E | | | | H | | | | V | | | A | | | E | | | F | | | K | | | R | | | SPVPIVLAASVEEQEETPS | | | |  |  |
| *Enterococcus faecalis* | | | | | | | | | | | | | | | | | | | | | | | | | | | | | | | | | | | | | | | | | | | | | | | | | | | | | | | | | S | L | | A | | R | | G | | I | | D | | D | | | V | | | A | | | T | | | | | M | | | | Y | | | | K | | | Y | | | N | | | | I | | | | I | | | | L | | | | | A | | | | N | | | | S | | | | | | | | | | | | D | | | | | | | | | | | | | | | | | | | | | G | | | | | | | | | | | | | | | | | | D | | | | D | | | | Q | | | | | K | | | | E | | V | | | | | T | | | | V | | | | L | | | N | | | | N | | | | L | | | | | L | | | | | A | | | K | | | Q | | | | V | | | | D | | | | G | | | | I | | | | I | | | | F | | | M | | | | | G | | | | H | | | | R | | | | | I | | | | T | | | | D | | | | D | | | | I | | | | R | | | G | | | E | | | F | | | S | | | R | | | SKTPVVLAGSIDPDEQVGS | | | |  |  |
| *Lactobacillus. casei*subsp.*casei* | | | | | | | | | | | | | | | | | | | | | | | | | | | | | | | | | | | | | | | | | | | | | | | | | | | | | | | | | S | L | | A | | R | | G | | I | | D | | D | | | V | | | A | | | T | | | | | M | | | | Y | | | | K | | | Y | | | N | | | | I | | | | I | | | | L | | | | | A | | | | N | | | | S | | | | | | | | | | | | D | | | | | | | | | | | | | | | | | | | | | E | | | | | | | | | | | | | | | | | | N | | | | N | | | | Q | | | | | K | | | | E | | V | | | | | T | | | | V | | | | L | | | N | | | | T | | | | L | | | | | L | | | | | A | | | K | | | Q | | | | V | | | | D | | | | G | | | | L | | | | I | | | | F | | | M | | | | | G | | | | H | | | | E | | | | | L | | | | T | | | | D | | | | S | | | | I | | | | R | | | A | | | E | | | F | | | S | | | R | | | SKTPVVLAGSIDPDEQVGS | | | |  |  |
| *Lactobacillus delbrueckii*subsp.*bulgaricus* | | | | | | | | | | | | | | | | | | | | | | | | | | | | | | | | | | | | | | | | | | | | | | | | | | | | | | | | | E | L | | S | | K | | G | | I | | D | | D | | | I | | | A | | | V | | | | | L | | | | Y | | | | K | | | Y | | | N | | | | I | | | | I | | | | I | | | | | S | | | | S | | | | V | | | | | | | | | | | | E | | | | | | | | | | | | | | | | | | | | | N | | | | | | | | | | | | | | | | | | R | | | | L | | | | M | | | | | K | | | | E | | D | | | | | A | | | | V | | | | I | | | Q | | | | G | | | | L | | | | | L | | | | | N | | | K | | | Q | | | | V | | | | D | | | | G | | | | V | | | | I | | | | Y | | | M | | | | | S | | | | N | | | | K | | | | | L | | | | S | | | | E | | | | E | | | | A | | | | A | | | E | | | A | | | F | | | K | | | R | | | TDTPVVLAGTVSDNLEFPS | | | |  |  |
| *Lactobacillus pentosus* | | | | | | | | | | | | | | | | | | | | | | | | | | | | | | | | | | | | | | | | | | | | | | | | | | | | | | | | | S | L | | A | | R | | G | | I | | D | | D | | | I | | | A | | | M | | | | | M | | | | Y | | | | K | | | Y | | | N | | | | I | | | | I | | | | L | | | | | T | | | | N | | | | S | | | | | | | | | | | | D | | | | | | | | | | | | | | | | | | | | | D | | | | | | | | | | | | | | | | | | A | | | | G | | | | E | | | | | Q | | | | E | | V | | | | | N | | | | V | | | | L | | | N | | | | T | | | | L | | | | | M | | | | | A | | | K | | | Q | | | | V | | | | D | | | | G | | | | V | | | | I | | | | F | | | M | | | | | G | | | | N | | | | H | | | | | I | | | | D | | | | D | | | | K | | | | L | | | | R | | | A | | | E | | | F | | | K | | | R | | | AKAPVVLAGTVDPNNETPS | | | |  |  |
| *Lactobacillus plantarum* | | | | | | | | | | | | | | | | | | | | | | | | | | | | | | | | | | | | | | | | | | | | | | | | | | | | | | | | | A | L | | R | | P | | G | | I | | D | | D | | | I | | | A | | | M | | | | | M | | | | Y | | | | K | | | Y | | | N | | | | I | | | | I | | | | L | | | | | T | | | | N | | | | S | | | | | | | | | | | | D | | | | | | | | | | | | | | | | | | | | | D | | | | | | | | | | | | | | | | | | A | | | | G | | | | E | | | | | Q | | | | E | | V | | | | | N | | | | V | | | | L | | | N | | | | T | | | | L | | | | | M | | | | | G | | | K | | | Q | | | | V | | | | D | | | | G | | | | V | | | | I | | | | F | | | M | | | | | G | | | | N | | | | Y | | | | | I | | | | D | | | | D | | | | K | | | | L | | | | R | | | A | | | E | | | F | | | K | | | R | | | AKAPVVLAGTVDPNNESPS | | | |  |  |
| *Lactobacillus reuteri* | | | | | | | | | | | | | | | | | | | | | | | | | | | | | | | | | | | | | | | | | | | | | | | | | | | | | | | | | E | L | | A | | L | | G | | I | | D | | D | | | V | | | A | | | S | | | | | M | | | | Y | | | | K | | | Y | | | N | | | | I | | | | I | | | | L | | | | | T | | | | N | | | | S | | | | | | | | | | | | D | | | | | | | | | | | | | | | | | | | | | S | | | | | | | | | | | | | | | | | | D | | | | D | | | | E | | | | | K | | | | I | | L | | | | | K | | | | V | | | | V | | | R | | | | S | | | | L | | | | | L | | | | | A | | | K | | | Q | | | | V | | | | D | | | | G | | | | L | | | | I | | | | F | | | M | | | | | G | | | | H | | | | D | | | | | V | | | | S | | | | D | | | | D | | | | L | | | | R | | | N | | | E | | | F | | | E | | | S | | | TNTPVVVAGSVVNDDALPS | | | |  |  |
| *Lactobacillus sakei*subsp. *sakei* | | | | | | | | | | | | | | | | | | | | | | | | | | | | | | | | | | | | | | | | | | | | | | | | | | | | | | | | | S | L | | A | | R | | G | | I | | D | | D | | | V | | | A | | | T | | | | | M | | | | Y | | | | K | | | Y | | | N | | | | I | | | | I | | | | L | | | | | A | | | | N | | | | S | | | | | | | | | | | | D | | | | | | | | | | | | | | | | | | | | | E | | | | | | | | | | | | | | | | | | N | | | | N | | | | Q | | | | | K | | | | E | | V | | | | | Q | | | | V | | | | L | | | N | | | | T | | | | L | | | | | L | | | | | A | | | K | | | Q | | | | V | | | | D | | | | G | | | | L | | | | I | | | | Y | | | M | | | | | G | | | | H | | | | S | | | | | I | | | | S | | | | D | | | | A | | | | I | | | | R | | | A | | | E | | | F | | | A | | | R | | | SKTPIVLAGSIDPDEQVGS | | | |  |  |
| *Lactococcus lactis*AM2 | | | | | | | | | | | | | | | | | | | | | | | | | | | | | | | | | | | | | | | | | | | | | | | | | | | | | | | | | A | I | | T | | R | | G | | V | | D | | D | | | I | | | A | | | S | | | | | M | | | | Y | | | | K | | | Y | | | N | | | | M | | | | I | | | | L | | | | | A | | | | N | | | | S | | | | | | | | | | | | D | | | | | | | | | | | | | | | | | | | | | N | | | | | | | | | | | | | | | | | | D | | | | V | | | | E | | | | | K | | | | E | | E | | | | | K | | | | V | | | | L | | | E | | | | T | | | | F | | | | | L | | | | | S | | | K | | | Q | | | | V | | | | D | | | | G | | | | I | | | | V | | | | Y | | | M | | | | | G | | | | S | | | | S | | | | | L | | | | D | | | | E | | | | K | | | | I | | | | R | | | T | | | S | | | L | | | K | | | N | | | SRTPVVLVGTIDGDKEIPS | | | |  |  |
| *Lactococcus lactis*LMG9447 | | | | | | | | | | | | | | | | | | | | | | | | | | | | | | | | | | | | | | | | | | | | | | | | | | | | | | | | | A | I | | T | | R | | G | | V | | D | | D | | | I | | | A | | | S | | | | | M | | | | Y | | | | K | | | Y | | | N | | | | M | | | | I | | | | L | | | | | A | | | | N | | | | I | | | | | | | | | | | | D | | | | | | | | | | | | | | | | | | | | | N | | | | | | | | | | | | | | | | | | D | | | | V | | | | E | | | | | K | | | | E | | Q | | | | | K | | | | V | | | | L | | | E | | | | T | | | | F | | | | | L | | | | | S | | | K | | | Q | | | | V | | | | D | | | | G | | | | I | | | | V | | | | Y | | | M | | | | | G | | | | S | | | | S | | | | | L | | | | D | | | | E | | | | K | | | | I | | | | R | | | T | | | S | | | L | | | N | | | N | | | STTPVVLVGTIYGDK---- | | | |  |  |
| *Lactococcus lactis*P7304 | | | | | | | | | | | | | | | | | | | | | | | | | | | | | | | | | | | | | | | | | | | | | | | | | | | | | | | | | A | I | | T | | R | | G | | V | | D | | D | | | I | | | A | | | S | | | | | M | | | | Y | | | | K | | | Y | | | N | | | | M | | | | I | | | | L | | | | | A | | | | N | | | | S | | | | | | | | | | | | D | | | | | | | | | | | | | | | | | | | | | N | | | | | | | | | | | | | | | | | | D | | | | V | | | | E | | | | | K | | | | E | | E | | | | | K | | | | V | | | | L | | | E | | | | T | | | | F | | | | | L | | | | | S | | | K | | | Q | | | | V | | | | D | | | | G | | | | I | | | | V | | | | Y | | | M | | | | | G | | | | S | | | | S | | | | | L | | | | D | | | | E | | | | K | | | | I | | | | R | | | T | | | S | | | L | | | K | | | N | | | SRTPVVLVGTIDGDKEIPS | | | |  |  |
| *Lactococcus lactis*subsp.*cremoris*MG1363 | | | | | | | | | | | | | | | | | | | | | | | | | | | | | | | | | | | | | | | | | | | | | | | | | | | | | | | | | A | I | | T | | R | | G | | V | | D | | D | | | I | | | A | | | S | | | | | M | | | | Y | | | | K | | | Y | | | N | | | | M | | | | I | | | | L | | | | | A | | | | N | | | | S | | | | | | | | | | | | D | | | | | | | | | | | | | | | | | | | | | N | | | | | | | | | | | | | | | | | | D | | | | V | | | | E | | | | | K | | | | E | | E | | | | | K | | | | V | | | | L | | | E | | | | T | | | | F | | | | | L | | | | | S | | | K | | | Q | | | | V | | | | D | | | | G | | | | I | | | | V | | | | Y | | | M | | | | | G | | | | S | | | | S | | | | | L | | | | D | | | | E | | | | K | | | | I | | | | R | | | T | | | S | | | L | | | K | | | N | | | SRTPVVLVGTIDGDKEIPS | | | |  |  |
| *Lactococcus lactis*subsp.*lactis*Il1403 | | | | | | | | | | | | | | | | | | | | | | | | | | | | | | | | | | | | | | | | | | | | | | | | | | | | | | | | | A | I | | T | | R | | G | | V | | D | | D | | | I | | | D | | | S | | | | | M | | | | Y | | | | K | | | Y | | | N | | | | M | | | | I | | | | L | | | | | A | | | | N | | | | S | | | | | | | | | | | | D | | | | | | | | | | | | | | | | | | | | | N | | | | | | | | | | | | | | | | | | D | | | | V | | | | E | | | | | K | | | | E | | E | | | | | K | | | | V | | | | L | | | E | | | | T | | | | F | | | | | L | | | | | S | | | K | | | Q | | | | V | | | | D | | | | G | | | | I | | | | V | | | | Y | | | M | | | | | G | | | | S | | | | S | | | | | L | | | | D | | | | E | | | | K | | | | I | | | | R | | | T | | | S | | | L | | | K | | | N | | | SRTPVVLVGTIDGDKEIPS | | | |  |  |
| *Lactococcus lactis*subsp.*lactis*KF147 | | | | | | | | | | | | | | | | | | | | | | | | | | | | | | | | | | | | | | | | | | | | | | | | | | | | | | | | | A | I | | T | | R | | G | | V | | D | | D | | | I | | | A | | | S | | | | | M | | | | Y | | | | K | | | Y | | | N | | | | M | | | | I | | | | L | | | | | A | | | | N | | | | S | | | | | | | | | | | | D | | | | | | | | | | | | | | | | | | | | | N | | | | | | | | | | | | | | | | | | D | | | | V | | | | E | | | | | K | | | | E | | E | | | | | K | | | | V | | | | L | | | E | | | | T | | | | F | | | | | L | | | | | S | | | K | | | Q | | | | V | | | | D | | | | G | | | | I | | | | V | | | | Y | | | M | | | | | G | | | | S | | | | S | | | | | L | | | | D | | | | E | | | | K | | | | I | | | | R | | | T | | | S | | | L | | | K | | | N | | | SRTPVVLVGTIDGDKEIPS | | | |  |  |
| *Lactococcus lactis*UC317 | | | | | | | | | | | | | | | | | | | | | | | | | | | | | | | | | | | | | | | | | | | | | | | | | | | | | | | | | A | I | | T | | R | | G | | V | | D | | D | | | I | | | A | | | S | | | | | M | | | | Y | | | | K | | | Y | | | N | | | | M | | | | I | | | | L | | | | | A | | | | N | | | | S | | | | | | | | | | | | D | | | | | | | | | | | | | | | | | | | | | N | | | | | | | | | | | | | | | | | | D | | | | V | | | | E | | | | | K | | | | E | | K | | | | | K | | | | V | | | | L | | | E | | | | T | | | | F | | | | | L | | | | | S | | | K | | | Q | | | | V | | | | D | | | | G | | | | I | | | | V | | | | Y | | | M | | | | | G | | | | S | | | | S | | | | | S | | | | D | | | | E | | | | K | | | | I | | | | K | | | T | | | S | | | L | | | K | | | N | | | SRTPVVFFGTIDGDKEIPP | | | |  |  |
| *Listeria monocytogenes* | | | | | | | | | | | | | | | | | | | | | | | | | | | | | | | | | | | | | | | | | | | | | | | | | | | | | | | | | E | L | | A | | R | | G | | I | | E | | D | | | I | | | A | | | T | | | | | M | | | | Y | | | | K | | | Y | | | N | | | | I | | | | I | | | | L | | | | | S | | | | N | | | | S | | | | | | | | | | | | D | | | | | | | | | | | | | | | | | | | | | E | | | | | | | | | | | | | | | | | | N | | | | E | | | | D | | | | | K | | | | E | | L | | | | | Q | | | | V | | | | L | | | N | | | | T | | | | L | | | | | L | | | | | G | | | K | | | Q | | | | V | | | | D | | | | G | | | | I | | | | I | | | | Y | | | M | | | | | G | | | | E | | | | R | | | | | I | | | | S | | | | E | | | | Q | | | | L | | | | Q | | | E | | | E | | | F | | | D | | | R | | | SPAPVVLAGAVDMENKFAS | | | |  |  |
| *Paenibacillus mucilaginosus* | | | | | | | | | | | | | | | | | | | | | | | | | | | | | | | | | | | | | | | | | | | | | | | | | | | | | | | | | E | V | | A | | R | | G | | I | | E | | D | | | I | | | A | | | N | | | | | M | | | | Y | | | | H | | | Y | | | N | | | | I | | | | I | | | | L | | | | | C | | | | N | | | | A | | | | | | | | | | | | D | | | | | | | | | | | | | | | | | | | | | K | | | | | | | | | | | | | | | | | | K | | | | K | | | | E | | | | | K | | | | E | | I | | | | | R | | | | V | | | | I | | | N | | | | T | | | | L | | | | | L | | | | | E | | | K | | | Q | | | | V | | | | D | | | | G | | | | L | | | | L | | | | F | | | M | | | | | G | | | | G | | | | A | | | | | V | | | | T | | | | E | | | | D | | | | H | | | | I | | | Q | | | A | | | F | | | K | | | T | | | SSVPVVLCATADEQRVIPS | | | |  |  |
| *Staphalococcus aureus*subsp.*aureus* | | | | | | | | | | | | | | | | | | | | | | | | | | | | | | | | | | | | | | | | | | | | | | | | | | | | | | | | | Q | L | | A | | R | | G | | L | | E | | D | | | I | | | A | | | I | | | | | M | | | | Y | | | | K | | | Y | | | H | | | | S | | | | I | | | | I | | | | | S | | | | N | | | | S | | | | | | | | | | | | D | | | | | | | | | | | | | | | | | | | | | N | | | | | | | | | | | | | | | | | | D | | | | P | | | | E | | | | | K | | | | E | | K | | | | | E | | | | I | | | | F | | | N | | | | N | | | | L | | | | | L | | | | | S | | | K | | | Q | | | | V | | | | D | | | | G | | | | I | | | | I | | | | F | | | L | | | | | G | | | | G | | | | T | | | | | I | | | | T | | | | E | | | | E | | | | M | | | | K | | | E | | | L | | | I | | | N | | | Q | | | SSVPVVVSGTNGKDAHIAS | | | |  |  |
| *Staphalococcus xylosus* | | | | | | | | | | | | | | | | | | | | | | | | | | | | | | | | | | | | | | | | | | | | | | | | | | | | | | | | | Q | L | | A | | R | | G | | L | | E | | D | | | I | | | A | | | T | | | | | M | | | | Y | | | | K | | | Y | | | H | | | | S | | | | I | | | | I | | | | | S | | | | N | | | | S | | | | | | | | | | | | D | | | | | | | | | | | | | | | | | | | | | N | | | | | | | | | | | | | | | | | | D | | | | P | | | | S | | | | | K | | | | E | | K | | | | | E | | | | I | | | | F | | | N | | | | N | | | | L | | | | | L | | | | | S | | | K | | | Q | | | | V | | | | D | | | | G | | | | I | | | | I | | | | F | | | L | | | | | G | | | | G | | | | T | | | | | I | | | | S | | | | E | | | | E | | | | I | | | | K | | | D | | | L | | | I | | | N | | | K | | | SSVPVVVSGTNGKDEGISS | | | |  |  |
| *Staphalococcus bovis* | | | | | | | | | | | | | | | | | | | | | | | | | | | | | | | | | | | | | | | | | | | | | | | | | | | | | | | | | I | L | | A | | K | | G | | I | | D | | D | | | I | | | A | | | A | | | | | M | | | | Y | | | | K | | | Y | | | N | | | | I | | | | V | | | | L | | | | | A | | | | S | | | | S | | | | | | | | | | | | D | | | | | | | | | | | | | | | | | | | | | E | | | | | | | | | | | | | | | | | | D | | | | D | | | | D | | | | | K | | | | E | | V | | | | | N | | | | V | | | | V | | | N | | | | T | | | | L | | | | | F | | | | | A | | | K | | | Q | | | | V | | | | D | | | | G | | | | I | | | | I | | | | F | | | M | | | | | G | | | | H | | | | H | | | | | L | | | | T | | | | E | | | | K | | | | I | | | | R | | | A | | | E | | | F | | | S | | | R | | | SRTPVVLAGTVDLEHQLPS | | | |  |  |
| *Streptococcus equi*subsp. *zooepidemicus* | | | | | | | | | | | | | | | | | | | | | | | | | | | | | | | | | | | | | | | | | | | | | | | | | | | | | | | | | I | L | | A | | K | | G | | I | | D | | D | | | I | | | A | | | A | | | | | M | | | | Y | | | | K | | | Y | | | N | | | | I | | | | V | | | | L | | | | | A | | | | S | | | | S | | | | | | | | | | | | D | | | | | | | | | | | | | | | | | | | | | E | | | | | | | | | | | | | | | | | | D | | | | D | | | | D | | | | | K | | | | E | | V | | | | | N | | | | V | | | | V | | | N | | | | T | | | | L | | | | | F | | | | | A | | | K | | | Q | | | | V | | | | D | | | | G | | | | I | | | | I | | | | F | | | M | | | | | G | | | | H | | | | H | | | | | L | | | | T | | | | D | | | | K | | | | I | | | | R | | | A | | | E | | | F | | | S | | | R | | | SRTPIVLAGTVDLDHQLPS | | | |  |  |
| *Streptococcus gallolyticus* | | | | | | | | | | | | | | | | | | | | | | | | | | | | | | | | | | | | | | | | | | | | | | | | | | | | | | | | | I | L | | A | | K | | G | | I | | D | | D | | | I | | | A | | | A | | | | | M | | | | Y | | | | K | | | Y | | | N | | | | I | | | | V | | | | L | | | | | A | | | | S | | | | S | | | | | | | | | | | | D | | | | | | | | | | | | | | | | | | | | | E | | | | | | | | | | | | | | | | | | D | | | | D | | | | D | | | | | K | | | | E | | V | | | | | N | | | | V | | | | V | | | N | | | | T | | | | L | | | | | F | | | | | A | | | K | | | Q | | | | V | | | | D | | | | G | | | | I | | | | I | | | | F | | | M | | | | | G | | | | H | | | | H | | | | | L | | | | T | | | | E | | | | K | | | | I | | | | R | | | A | | | E | | | F | | | S | | | R | | | SRTPVVLAGTVDLEHQLPS | | | |  |  |
| *Streptococcus mutans* | | | | | | | | | | | | | | | | | | | | | | | | | | | | | | | | | | | | | | | | | | | | | | | | | | | | | | | | | I | L | | A | | K | | G | | I | | D | | D | | | I | | | A | | | T | | | | | M | | | | Y | | | | K | | | Y | | | N | | | | I | | | | V | | | | L | | | | | A | | | | S | | | | S | | | | | | | | | | | | D | | | | | | | | | | | | | | | | | | | | | E | | | | | | | | | | | | | | | | | | D | | | | D | | | | D | | | | | K | | | | E | | V | | | | | N | | | | V | | | | I | | | N | | | | T | | | | L | | | | | F | | | | | A | | | K | | | Q | | | | V | | | | D | | | | G | | | | I | | | | I | | | | F | | | M | | | | | G | | | | H | | | | H | | | | | L | | | | T | | | | E | | | | K | | | | I | | | | R | | | A | | | E | | | F | | | S | | | R | | | ARTPVVLSGTVDLEHQLPS | | | |  |  |
| *Streptococcus thermophilus* | | | | | | | | | | | | | | | | | | | | | | | | | | | | | | | | | | | | | | | | | | | | | | | | | | | | | | | | | T | L | | A | | K | | G | | I | | D | | D | | | I | | | A | | | T | | | | | M | | | | Y | | | | K | | | Y | | | N | | | | I | | | | V | | | | L | | | | | A | | | | S | | | | S | | | | | | | | | | | | D | | | | | | | | | | | | | | | | | | | | | D | | | | | | | | | | | | | | | | | | N | | | | E | | | | D | | | | | H | | | | E | | V | | | | | T | | | | V | | | | I | | | H | | | | S | | | | L | | | | | I | | | | | S | | | K | | | Q | | | | V | | | | D | | | | G | | | | I | | | | I | | | | F | | | M | | | | | G | | | | H | | | | H | | | | | L | | | | T | | | | E | | | | K | | | | I | | | | R | | | A | | | E | | | F | | | S | | | R | | | TRTPIVLAGTVDLEHQLPS | | | |  |  |
|  | | | | | | | | | | | | | | | | | | | | | | | | | | | | | | | | | | | | | | | | | | | | | | | | | | | | | | | | | | | | | | | | | | | | | | | | | | | | | | | | | | | | | | | | | | | | | | | | | | | | | | | | | | | | | | | | | | | | | | | | | | | | | | | | | | | | | | | | | | | | | | | | | | | | | | | | | | | | | | | | | | | | | | | | | | | | | | | | | | | | | | | | | | | | | | | | | | | | | | | | | | | | | | | | | | | | | | | | | | | | | | | | | | | | | | | | | | | | | | | | | | | | | | | | | | | | | | | | | | | | | | | | | | | | | | | | | | | | | | | | | | | | | | | | | | | | | | | | | | | | | | | | | | | | | | | | | | | |  | | | |  |  |
| *Bacillus amyloliquefaciens* | | | | | | | | | | | | | | | | | | | | | | | | | | | | | | | | | | | | | | | | | | | | | | | | | | | | | | | | | V | A | | I | | D | | Y | | E | | Q | | A | | | I | | | Y | | | D | | | A | | | V | | | | | | K | | | L | | | | F | | | I | | | | D | | | | K | | | | G | | | | H | | | | R | | | | | | | | | | D | | | | | | | | | | | | | | | | | | | | | I | | | | | | | | | | | | | | | | | | | | | A | | | | F | | | | V | | | | S | | | | G | | | | | | P | | | | M | | | | | Q | | | | E | | | | P | | | | I | | | | N | | | | R | | | S | | | K | | | | K | | | | L | | | | Q | | | | G | | | | Y | | | | K | | | | R | | | | | A | | | | L | | | | E | | | | E | | | | A | | | | A | | | | I | | | | P | | | | F | | | N | | | E | | | Q | | | F | | | | V | | | A | | | EGDYTYDSGMEALQSLMGL | | | | |
| *Bacillus anthracis* | | | | | | | | | | | | | | | | | | | | | | | | | | | | | | | | | | | | | | | | | | | | | | | | | | | | | | | | | V | N | | I | | D | | Y | | T | | Q | | A | | | A | | | Y | | | D | | | A | | | M | | | | | | K | | | H | | | | F | | | I | | | | E | | | | Q | | | | G | | | | H | | | | K | | | | | | | | | | R | | | | | | | | | | | | | | | | | | | | | I | | | | | | | | | | | | | | | | | | | | | G | | | | F | | | | V | | | | S | | | | G | | | | | | P | | | | F | | | | | I | | | | D | | | | K | | | | A | | | | G | | | | S | | | A | | | K | | | | K | | | | L | | | | Q | | | | G | | | | Y | | | | K | | | | K | | | | | A | | | | L | | | | E | | | | E | | | | A | | | | G | | | | I | | | | S | | | | Y | | | D | | | E | | | N | | | L | | | | V | | | I | | | DGDYTYDSGIEAFEKLWGL | | | | |
| *Bacillus licheniformis* | | | | | | | | | | | | | | | | | | | | | | | | | | | | | | | | | | | | | | | | | | | | | | | | | | | | | | | | | V | A | | I | | D | | Y | | E | | Q | | A | | | I | | | Y | | | D | | | A | | | A | | | | | | T | | | M | | | | L | | | I | | | | E | | | | K | | | | G | | | | H | | | | K | | | | | | | | | | R | | | | | | | | | | | | | | | | | | | | | L | | | | | | | | | | | | | | | | | | | | | A | | | | F | | | | V | | | | S | | | | G | | | | | | P | | | | M | | | | | A | | | | E | | | | P | | | | V | | | | N | | | | Q | | | A | | | K | | | | K | | | | L | | | | Q | | | | G | | | | F | | | | K | | | | R | | | | | A | | | | L | | | | G | | | | D | | | | K | | | | G | | | | L | | | | Q | | | | F | | | K | | | E | | | E | | | Y | | | | V | | | V | | | EGDYTYDSGMEALEALMKL | | | | |
| *Bacillus megaterium* | | | | | | | | | | | | | | | | | | | | | | | | | | | | | | | | | | | | | | | | | | | | | | | | | | | | | | | | | V | T | | I | | D | | Y | | E | | Q | | A | | | A | | | F | | | D | | | A | | | V | | | | | | Q | | | S | | | | L | | | I | | | | D | | | | S | | | | G | | | | H | | | | K | | | | | | | | | | N | | | | | | | | | | | | | | | | | | | | | I | | | | | | | | | | | | | | | | | | | | | A | | | | F | | | | V | | | | S | | | | G | | | | | | T | | | | L | | | | | E | | | | E | | | | P | | | | I | | | | N | | | | H | | | A | | | K | | | | K | | | | V | | | | K | | | | G | | | | Y | | | | K | | | | R | | | | | A | | | | L | | | | T | | | | E | | | | S | | | | G | | | | L | | | | P | | | | V | | | R | | | D | | | S | | | Y | | | | I | | | V | | | EGDYTYDSGIEAVEKLLEE | | | | |
| *Bacillus subtilis* | | | | | | | | | | | | | | | | | | | | | | | | | | | | | | | | | | | | | | | | | | | | | | | | | | | | | | | | | V | A | | I | | D | | Y | | E | | Q | | A | | | I | | | Y | | | D | | | A | | | V | | | | | | K | | | L | | | | L | | | V | | | | D | | | | K | | | | G | | | | H | | | | T | | | | | | | | | | D | | | | | | | | | | | | | | | | | | | | | I | | | | | | | | | | | | | | | | | | | | | A | | | | F | | | | V | | | | S | | | | G | | | | | | P | | | | M | | | | | A | | | | E | | | | P | | | | I | | | | N | | | | R | | | S | | | K | | | | K | | | | L | | | | Q | | | | G | | | | Y | | | | K | | | | R | | | | | A | | | | L | | | | E | | | | E | | | | A | | | | N | | | | L | | | | P | | | | F | | | N | | | E | | | Q | | | F | | | | V | | | A | | | EGDYTYDSGLEALQHLMSL | | | | |
| *Enterococcus faecalis* | | | | | | | | | | | | | | | | | | | | | | | | | | | | | | | | | | | | | | | | | | | | | | | | | | | | | | | | | V | N | | I | | D | | Y | | T | | E | | A | | | T | | | K | | | D | | | A | | | T | | | | | | A | | | T | | | | L | | | A | | | | K | | | | N | | | | G | | | | N | | | | K | | | | | | | | | | K | | | | | | | | | | | | | | | | | | | | | I | | | | | | | | | | | | | | | | | | | | | A | | | | F | | | | V | | | | S | | | | G | | | | | | A | | | | L | | | | | I | | | | D | | | | P | | | | I | | | | N | | | | G | | | Q | | | N | | | | R | | | | M | | | | K | | | | G | | | | Y | | | | K | | | | E | | | | | A | | | | L | | | | A | | | | E | | | | N | | | | G | | | | L | | | | S | | | | Y | | | N | | | E | | | G | | | L | | | | V | | | F | | | ESEYKFKAGINLAERVRN- | | | | |
| *Lactobacillus. casei*subsp.*casei* | | | | | | | | | | | | | | | | | | | | | | | | | | | | | | | | | | | | | | | | | | | | | | | | | | | | | | | | | V | N | | I | | D | | Y | | V | | A | | A | | | V | | | E | | | E | | | A | | | T | | | | | | R | | | Q | | | | L | | | L | | | | E | | | | S | | | | G | | | | N | | | | K | | | | | | | | | | R | | | | | | | | | | | | | | | | | | | | | V | | | | | | | | | | | | | | | | | | | | | A | | | | L | | | | A | | | | T | | | | G | | | | | | S | | | | L | | | | | T | | | | H | | | | P | | | | I | | | | N | | | | G | | | Q | | | F | | | | R | | | | L | | | | K | | | | G | | | | Y | | | | K | | | | Q | | | | | A | | | | L | | | | E | | | | K | | | | A | | | | G | | | | V | | | | A | | | | Y | | | D | | | E | | | S | | | L | | | | I | | | F | | | ENEPSYQAGLALFDKLQK- | | | | |
| *Lactobacillus delbrueckii*subsp.*bulgaricus* | | | | | | | | | | | | | | | | | | | | | | | | | | | | | | | | | | | | | | | | | | | | | | | | | | | | | | | | | A | N | | I | | D | | Y | | K | | K | | A | | | D | | | T | | | E | | | A | | | L | | | | | | N | | | L | | | | L | | | L | | | | N | | | | D | | | | G | | | | K | | | | K | | | | | | | | | | K | | | | | | | | | | | | | | | | | | | | | L | | | | | | | | | | | | | | | | | | | | | A | | | | L | | | | I | | | | V | | | | G | | | | | | D | | | | K | | | | | E | | | | A | | | | P | | | | I | | | | N | | | | R | | | N | | | H | | | | R | | | | I | | | | P | | | | A | | | | F | | | | E | | | | K | | | | | F | | | | V | | | | A | | | | D | | | | N | | | | - | | | | - | | | | E | | | | L | | | E | | | G | | | C | | | E | | | | I | | | F | | | DNIKDYSDGYNLYPELAK- | | | | |
| *Lactobacillus pentosus* | | | | | | | | | | | | | | | | | | | | | | | | | | | | | | | | | | | | | | | | | | | | | | | | | | | | | | | | | V | N | | I | | D | | Y | | A | | A | | A | | | V | | | E | | | E | | | A | | | V | | | | | | T | | | N | | | | L | | | I | | | | G | | | | R | | | | G | | | | H | | | | K | | | | | | | | | | K | | | | | | | | | | | | | | | | | | | | | I | | | | | | | | | | | | | | | | | | | | | A | | | | L | | | | A | | | | L | | | | G | | | | | | S | | | | L | | | | | S | | | | Q | | | | S | | | | I | | | | N | | | | A | | | E | | | Y | | | | R | | | | L | | | | T | | | | G | | | | Y | | | | K | | | | R | | | | | A | | | | L | | | | T | | | | K | | | | A | | | | K | | | | I | | | | P | | | | F | | | D | | | D | | | A | | | L | | | | V | | | Y | | | EAGYSYDAGRKLQPVIAD- | | | | |
| *Lactobacillus plantarum* | | | | | | | | | | | | | | | | | | | | | | | | | | | | | | | | | | | | | | | | | | | | | | | | | | | | | | | | | V | N | | I | | D | | Y | | A | | A | | A | | | V | | | E | | | E | | | V | | | V | | | | | | T | | | N | | | | L | | | I | | | | A | | | | R | | | | G | | | | H | | | | K | | | | | | | | | | K | | | | | | | | | | | | | | | | | | | | | I | | | | | | | | | | | | | | | | | | | | | A | | | | L | | | | A | | | | L | | | | G | | | | | | S | | | | L | | | | | S | | | | Q | | | | S | | | | I | | | | N | | | | A | | | R | | | I | | | | P | | | | T | | | | D | | | | G | | | | L | | | | Q | | | | T | | | | | G | | | | L | | | | N | | | | Q | | | | G | | | | K | | | | I | | | | P | | | | F | | | D | | | D | | | A | | | L | | | | V | | | Y | | | EAGYSYDAGRKLQPVIAD- | | | | |
| *Lactobacillus reuteri* | | | | | | | | | | | | | | | | | | | | | | | | | | | | | | | | | | | | | | | | | | | | | | | | | | | | | | | | | V | R | | I | | N | | Y | | Q | | A | | A | | | A | | | K | | | E | | | A | | | T | | | | | | E | | | F | | | | L | | | L | | | | K | | | | H | | | | G | | | | D | | | | Q | | | | | | | | | | Q | | | | | | | | | | | | | | | | | | | | | V | | | | | | | | | | | | | | | | | | | | | A | | | | Y | | | | I | | | | T | | | | G | | | | | | P | | | | L | | | | | R | | | | Y | | | | S | | | | I | | | | N | | | | G | | | E | | | D | | | | R | | | | L | | | | N | | | | G | | | | Y | | | | K | | | | E | | | | | A | | | | L | | | | A | | | | N | | | | N | | | | N | | | | V | | | | P | | | | F | | | N | | | E | | | S | | | L | | | | V | | | I | | | ETDGSYQAGYAKAQEVIE- | | | | |
| *Lactobacillus sakei*subsp. *sakei* | | | | | | | | | | | | | | | | | | | | | | | | | | | | | | | | | | | | | | | | | | | | | | | | | | | | | | | | | V | N | | I | | D | | Y | | V | | A | | A | | | V | | | K | | | D | | | A | | | T | | | | | | L | | | K | | | | L | | | I | | | | K | | | | N | | | | G | | | | N | | | | E | | | | | | | | | | K | | | | | | | | | | | | | | | | | | | | | V | | | | | | | | | | | | | | | | | | | | | A | | | | F | | | | I | | | | C | | | | G | | | | | | N | | | | L | | | | | E | | | | F | | | | P | | | | I | | | | N | | | | S | | | K | | | Y | | | | R | | | | L | | | | R | | | | G | | | | Y | | | | K | | | | D | | | | | A | | | | L | | | | N | | | | E | | | | A | | | | G | | | | I | | | | P | | | | Y | | | D | | | E | | | S | | | L | | | | I | | | F | | | ETEYSYSAGEALFGKVQA- | | | | |
| *Lactococcus lactis*AM2 | | | | | | | | | | | | | | | | | | | | | | | | | | | | | | | | | | | | | | | | | | | | | | | | | | | | | | | | | V | N | | I | | D | | Y | | H | | L | | A | | | D | | | Y | | | Q | | | S | | | T | | | | | | T | | | K | | | | L | | | I | | | | E | | | | N | | | | G | | | | N | | | | K | | | | | | | | | | K | | | | | | | | | | | | | | | | | | | | | I | | | | | | | | | | | | | | | | | | | | | A | | | | Y | | | | I | | | | M | | | | G | | | | | | S | | | | L | | | | | K | | | | D | | | | V | | | | E | | | | N | | | | - | | | T | | | E | | | | R | | | | M | | | | V | | | | G | | | | Y | | | | - | | | | - | | | | | - | | | | - | | | | - | | | | - | | | | - | | | | - | | | | - | | | | - | | | | - | | | - | | | - | | | - | | | - | | | | - | | | - | | | ------------------- | | | | |
| *Lactococcus lactis*LMG9447 | | | | | | | | | | | | | | | | | | | | | | | | | | | | | | | | | | | | | | | | | | | | | | | | | | | | | | | | | - | - | | - | | - | | - | | - | | - | | - | | | - | | | - | | | - | | | - | | | - | | | | | | - | | | - | | | | - | | | - | | | | - | | | | - | | | | - | | | | - | | | | - | | | | | | | | | | - | | | | | | | | | | | | | | | | | | | | | - | | | | | | | | | | | | | | | | | | | | | - | | | | - | | | | - | | | | - | | | | - | | | | | | - | | | | - | | | | | - | | | | - | | | | - | | | | - | | | | - | | | | - | | | - | | | - | | | | - | | | | - | | | | - | | | | - | | | | - | | | | - | | | | - | | | | | - | | | | - | | | | - | | | | - | | | | - | | | | - | | | | - | | | | - | | | | - | | | - | | | - | | | - | | | - | | | | - | | | - | | | ------------------- | | | | |
| *Lactococcus lactis*P7304 | | | | | | | | | | | | | | | | | | | | | | | | | | | | | | | | | | | | | | | | | | | | | | | | | | | | | | | | | V | N | | I | | D | | Y | | H | | L | | A | | | A | | | Y | | | Q | | | S | | | T | | | | | | T | | | K | | | | L | | | I | | | | E | | | | N | | | | G | | | | N | | | | K | | | | | | | | | | K | | | | | | | | | | | | | | | | | | | | | I | | | | | | | | | | | | | | | | | | | | | A | | | | Y | | | | I | | | | M | | | | G | | | | | | S | | | | L | | | | | K | | | | D | | | | V | | | | E | | | | N | | | | - | | | T | | | E | | | | R | | | | M | | | | V | | | | G | | | | Y | | | | Q | | | | E | | | | | A | | | | L | | | | L | | | | E | | | | A | | | | N | | | | I | | | | E | | | | F | | | D | | | E | | | N | | | L | | | | V | | | F | | | EGNYSYKQGKSLAERLLE- | | | | |
| *Lactococcus lactis*subsp.*cremoris*MG1363 | | | | | | | | | | | | | | | | | | | | | | | | | | | | | | | | | | | | | | | | | | | | | | | | | | | | | | | | | V | N | | I | | D | | Y | | H | | L | | A | | | A | | | Y | | | Q | | | S | | | T | | | | | | T | | | K | | | | L | | | I | | | | E | | | | N | | | | G | | | | N | | | | K | | | | | | | | | | K | | | | | | | | | | | | | | | | | | | | | I | | | | | | | | | | | | | | | | | | | | | A | | | | Y | | | | I | | | | M | | | | G | | | | | | S | | | | L | | | | | K | | | | D | | | | V | | | | E | | | | N | | | | - | | | T | | | E | | | | R | | | | M | | | | V | | | | G | | | | Y | | | | Q | | | | E | | | | | A | | | | L | | | | L | | | | E | | | | A | | | | N | | | | I | | | | E | | | | F | | | D | | | E | | | N | | | L | | | | V | | | F | | | EGNYSYEQGKSLAERLLE- | | | | |
| *Lactococcus lactis*subsp.*lactis*Il1403 | | | | | | | | | | | | | | | | | | | | | | | | | | | | | | | | | | | | | | | | | | | | | | | | | | | | | | | | | V | N | | I | | D | | Y | | H | | L | | A | | | A | | | Y | | | Q | | | S | | | T | | | | | | K | | | K | | | | L | | | I | | | | D | | | | S | | | | G | | | | N | | | | K | | | | | | | | | | K | | | | | | | | | | | | | | | | | | | | | I | | | | | | | | | | | | | | | | | | | | | A | | | | Y | | | | I | | | | M | | | | G | | | | | | S | | | | L | | | | | K | | | | D | | | | V | | | | E | | | | N | | | | - | | | T | | | E | | | | R | | | | M | | | | V | | | | G | | | | Y | | | | Q | | | | E | | | | | A | | | | L | | | | L | | | | E | | | | A | | | | N | | | | I | | | | E | | | | F | | | D | | | E | | | N | | | L | | | | V | | | F | | | EGNYSYEQGKALAERLLE- | | | | |
| *Lactococcus lactis*subsp.*lactis*KF147 | | | | | | | | | | | | | | | | | | | | | | | | | | | | | | | | | | | | | | | | | | | | | | | | | | | | | | | | | V | N | | I | | D | | Y | | H | | L | | A | | | A | | | Y | | | Q | | | S | | | T | | | | | | K | | | K | | | | L | | | I | | | | D | | | | S | | | | G | | | | N | | | | K | | | | | | | | | | K | | | | | | | | | | | | | | | | | | | | | I | | | | | | | | | | | | | | | | | | | | | A | | | | Y | | | | I | | | | M | | | | G | | | | | | S | | | | L | | | | | K | | | | D | | | | V | | | | E | | | | N | | | | - | | | T | | | E | | | | R | | | | M | | | | V | | | | G | | | | Y | | | | Q | | | | E | | | | | A | | | | L | | | | L | | | | E | | | | A | | | | N | | | | I | | | | E | | | | F | | | D | | | E | | | N | | | L | | | | V | | | F | | | EGNYSYEQGKALAERLLE- | | | | |
| *Lactococcus lactis*UC317 | | | | | | | | | | | | | | | | | | | | | | | | | | | | | | | | | | | | | | | | | | | | | | | | | | | | | | | | | V | N | | N | | D | | Y | | Q | | L | | A | | | A | | | C | | | Q | | | P | | | T | | | | | | K | | | Q | | | | L | | | I | | | | D | | | | Y | | | | G | | | | K | | | | K | | | | | | | | | | K | | | | | | | | | | | | | | | | | | | | | I | | | | | | | | | | | | | | | | | | | | | T | | | | Y | | | | F | | | | L | | | | W | | | | | | V | | | | F | | | | | E | | | | R | | | | S | | | | - | | | | - | | | | - | | | - | | | - | | | | - | | | | - | | | | - | | | | - | | | | - | | | | - | | | | - | | | | | - | | | | - | | | | - | | | | - | | | | - | | | | - | | | | - | | | | - | | | | - | | | - | | | - | | | - | | | - | | | | - | | | - | | | ------------------- | | | | |
| *Listeria monocytogenes* | | | | | | | | | | | | | | | | | | | | | | | | | | | | | | | | | | | | | | | | | | | | | | | | | | | | | | | | | V | N | | I | | D | | Y | | K | | Q | | A | | | T | | | K | | | E | | | A | | | V | | | | | | K | | | R | | | | F | | | V | | | | D | | | | N | | | | G | | | | H | | | | K | | | | | | | | | | Q | | | | | | | | | | | | | | | | | | | | | I | | | | | | | | | | | | | | | | | | | | | A | | | | F | | | | V | | | | S | | | | G | | | | | | S | | | | L | | | | | N | | | | E | | | | P | | | | V | | | | N | | | | R | | | E | | | M | | | | K | | | | L | | | | A | | | | G | | | | Y | | | | K | | | | E | | | | | A | | | | L | | | | E | | | | E | | | | A | | | | G | | | | I | | | | A | | | | Y | | | Q | | | E | | | D | | | Y | | | | I | | | I | | | EAKYNYNAGVKVWAELSAL | | | | |
| *Paenibacillus mucilaginosus* | | | | | | | | | | | | | | | | | | | | | | | | | | | | | | | | | | | | | | | | | | | | | | | | | | | | | | | | | V | D | | I | | D | | H | | E | | K | | A | | | A | | | F | | | D | | | A | | | V | | | | | | N | | | V | | | | L | | | L | | | | Q | | | | S | | | | G | | | | H | | | | T | | | | | | | | | | K | | | | | | | | | | | | | | | | | | | | | I | | | | | | | | | | | | | | | | | | | | | A | | | | M | | | | I | | | | S | | | | G | | | | | | T | | | | L | | | | | Q | | | | D | | | | P | | | | A | | | | N | | | | G | | | Y | | | A | | | | R | | | | Y | | | | Q | | | | G | | | | Y | | | | R | | | | K | | | | | A | | | | L | | | | E | | | | A | | | | A | | | | N | | | | I | | | | P | | | | I | | | D | | | E | | | D | | | Y | | | | V | | | R | | | IGNYRYESGMDVTKHFLEL | | | | |
| *Staphalococcus aureus*subsp.*aureus* | | | | | | | | | | | | | | | | | | | | | | | | | | | | | | | | | | | | | | | | | | | | | | | | | | | | | | | | | V | N | | I | | D | | F | | T | | E | | A | | | A | | | K | | | E | | | I | | | T | | | | | | G | | | K | | | | L | | | I | | | | E | | | | K | | | | G | | | | A | | | | K | | | | | | | | | | S | | | | | | | | | | | | | | | | | | | | | F | | | | | | | | | | | | | | | | | | | | | A | | | | L | | | | V | | | | G | | | | G | | | | | | E | | | | H | | | | | S | | | | K | | | | K | | | | A | | | | Q | | | | E | | | D | | | - | | | | V | | | | L | | | | A | | | | G | | | | L | | | | T | | | | E | | | | | V | | | | L | | | | N | | | | K | | | | N | | | | S | | | | L | | | | Q | | | | L | | | G | | | D | | | T | | | L | | | | N | | | C | | | SGAESYKEGVKAFAKMKG- | | | | |
| *Staphalococcus xylosus* | | | | | | | | | | | | | | | | | | | | | | | | | | | | | | | | | | | | | | | | | | | | | | | | | | | | | | | | | V | N | | I | | D | | F | | E | | S | | A | | | A | | | K | | | E | | | I | | | T | | | | | | E | | | H | | | | L | | | I | | | | E | | | | K | | | | G | | | | A | | | | K | | | | | | | | | | S | | | | | | | | | | | | | | | | | | | | | F | | | | | | | | | | | | | | | | | | | | | A | | | | F | | | | V | | | | G | | | | G | | | | | | D | | | | Y | | | | | S | | | | K | | | | K | | | | A | | | | Q | | | | E | | | D | | | - | | | | V | | | | L | | | | V | | | | G | | | | L | | | | K | | | | D | | | | | V | | | | L | | | | V | | | | Q | | | | H | | | | E | | | | L | | | | E | | | | L | | | D | | | E | | | Q | | | L | | | | I | | | F | | | NGNETYKDGLRAFESLAT- | | | | |
| *Staphalococcus bovis* | | | | | | | | | | | | | | | | | | | | | | | | | | | | | | | | | | | | | | | | | | | | | | | | | | | | | | | | | V | N | | I | | D | | Y | | K | | A | | A | | | V | | | A | | | D | | | V | | | V | | | | | | D | | | I | | | | L | | | A | | | | K | | | | N | | | | - | | | | N | | | | K | | | | | | | | | | D | | | | | | | | | | | | | | | | | | | | | I | | | | | | | | | | | | | | | | | | | | | A | | | | F | | | | V | | | | S | | | | G | | | | | | P | | | | L | | | | | I | | | | D | | | | D | | | | I | | | | N | | | | G | | | K | | | V | | | | R | | | | L | | | | A | | | | G | | | | Y | | | | K | | | | E | | | | | G | | | | L | | | | E | | | | K | | | | N | | | | N | | | | L | | | | S | | | | F | | | K | | | E | | | G | | | L | | | | V | | | F | | | EANYNYKDGYELAQRVMN- | | | | |
| *Streptococcus equi*subsp. *zooepidemicus* | | | | | | | | | | | | | | | | | | | | | | | | | | | | | | | | | | | | | | | | | | | | | | | | | | | | | | | | | V | N | | I | | D | | Y | | K | | A | | A | | | V | | | A | | | D | | | V | | | V | | | | | | D | | | I | | | | L | | | A | | | | K | | | | D | | | | - | | | | H | | | | Q | | | | | | | | | | S | | | | | | | | | | | | | | | | | | | | | I | | | | | | | | | | | | | | | | | | | | | A | | | | F | | | | V | | | | S | | | | G | | | | | | P | | | | L | | | | | I | | | | D | | | | D | | | | I | | | | N | | | | G | | | K | | | V | | | | R | | | | L | | | | A | | | | G | | | | Y | | | | K | | | | D | | | | | G | | | | L | | | | K | | | | K | | | | N | | | | K | | | | L | | | | D | | | | F | | | K | | | E | | | G | | | L | | | | V | | | F | | | EANYSYKEGFELAQRVIN- | | | | |
| *Streptococcus gallolyticus* | | | | | | | | | | | | | | | | | | | | | | | | | | | | | | | | | | | | | | | | | | | | | | | | | | | | | | | | | V | N | | I | | D | | Y | | K | | A | | A | | | V | | | A | | | D | | | V | | | V | | | | | | D | | | I | | | | L | | | A | | | | K | | | | N | | | | - | | | | N | | | | K | | | | | | | | | | E | | | | | | | | | | | | | | | | | | | | | I | | | | | | | | | | | | | | | | | | | | | A | | | | F | | | | V | | | | S | | | | G | | | | | | P | | | | L | | | | | I | | | | D | | | | D | | | | I | | | | N | | | | G | | | K | | | V | | | | R | | | | L | | | | A | | | | G | | | | Y | | | | K | | | | E | | | | | G | | | | L | | | | E | | | | K | | | | N | | | | G | | | | L | | | | S | | | | F | | | K | | | E | | | G | | | L | | | | V | | | F | | | EANYNYKDGFELAQRVIN- | | | | |
| *Streptococcus mutans* | | | | | | | | | | | | | | | | | | | | | | | | | | | | | | | | | | | | | | | | | | | | | | | | | | | | | | | | | V | N | | I | | D | | H | | S | | K | | A | | | A | | | Q | | | D | | | A | | | V | | | | | | A | | | L | | | | L | | | A | | | | K | | | | H | | | | - | | | | H | | | | D | | | | | | | | | | K | | | | | | | | | | | | | | | | | | | | | I | | | | | | | | | | | | | | | | | | | | | A | | | | F | | | | V | | | | S | | | | G | | | | | | P | | | | L | | | | | I | | | | D | | | | D | | | | I | | | | N | | | | G | | | K | | | V | | | | R | | | | L | | | | A | | | | G | | | | Y | | | | K | | | | E | | | | | G | | | | L | | | | K | | | | K | | | | K | | | | G | | | | L | | | | P | | | | F | | | K | | | E | | | G | | | L | | | | V | | | F | | | EAQYKYQEGYQLAQRVIN- | | | | |
| *Streptococcus thermophilus* | | | | | | | | | | | | | | | | | | | | | | | | | | | | | | | | | | | | | | | | | | | | | | | | | | | | | | | | | V | N | | I | | D | | Y | | K | | A | | A | | | V | | | E | | | D | | | C | | | V | | | | | | T | | | Q | | | | L | | | A | | | | K | | | | N | | | | - | | | | N | | | | E | | | | | | | | | | K | | | | | | | | | | | | | | | | | | | | | V | | | | | | | | | | | | | | | | | | | | | A | | | | F | | | | V | | | | S | | | | G | | | | | | P | | | | L | | | | | I | | | | D | | | | D | | | | I | | | | N | | | | G | | | K | | | L | | | | R | | | | L | | | | A | | | | G | | | | Y | | | | K | | | | S | | | | | G | | | | L | | | | E | | | | K | | | | N | | | | N | | | | L | | | | S | | | | Y | | | N | | | E | | | G | | | L | | | | V | | | F | | | EAKYSYKDGFELAQRVLN- | | | | |
|  | | | | | | | | | | | | | | | | | | | | | | | | | | | | | | | | | | | | | | | | | | | | | | | | | | | | | | | | | | | | | | | | | | | | | | | | | | | | | | | | | | | | | | | | | | | | | | | | | | | | | | | | | | | | | | | | | | | | | | | | | | | | | | | | | | | | | | | | | | | | | | | | | | | | | | | | | | | | | | | | | | | | | | | | | | | | | | | | | | | | | | | | | | | | | | | | | | | | | | | | | | | | | | | | | | | | | | | | | | | | | | | | | | | | | | | | | | | | | | | | | | | | | | | | | | | | | | | | | | | | | | | | | | | | | | | | | | | | | | | | | | | | | | | | | | | | | | | | | | | | | | | | | | | | | | | | | | |  | | | | | |  |
| *Bacillus amyloliquefaciens* | | | | | | | | | | | | | | | | | | | | | | | | | | | | | | | | | | | | | | | | | | | | | | | | | | | | | | | | | D | R | | K | | P | | T | | A | | I | | L | | | S | | | A | | | T | | | D | | | E | | | | M | | | | | A | | | L | | | G | | | | I | | | | I | | | | | | H | | | | A | | | | A | | | | | | | | | | | | | | | | Q | | | | | | | | | | | | | | | | | | | | | | | | | D | | | | | | | | | | | | Q | | | | G | | | | L | | | S | | | | I | | | | P | | | D | | | | D | | | | L | | | | | D | | | | I | | | | I | | | | | G | | | F | | | D | | | N | | | | T | | | R | | | | L | | | | | S | | | | L | | | | M | | | | | V | | | R | | | | P | | | | Q | | | | L | | | | S | | | | T | | | | V | | | | V | | | | Q | | | P | | | T | | | Y | | | | D | | | I | | | GAVAMRLLTKLMNKEP--- | | | | | |  |
| *Bacillus anthracis* | | | | | | | | | | | | | | | | | | | | | | | | | | | | | | | | | | | | | | | | | | | | | | | | | | | | | | | | | D | E | | K | | P | | S | | A | | I | | F | | | V | | | S | | | S | | | D | | | E | | | | M | | | | | A | | | L | | | G | | | | V | | | | I | | | | | | H | | | | A | | | | A | | | | | | | | | | | | | | | | Q | | | | | | | | | | | | | | | | | | | | | | | | | D | | | | | | | | | | | | A | | | | G | | | | L | | | N | | | | V | | | | P | | | T | | | | D | | | | V | | | | | E | | | | V | | | | L | | | | | G | | | F | | | D | | | N | | | | T | | | R | | | | L | | | | | A | | | | L | | | | M | | | | | V | | | R | | | | P | | | | Q | | | | L | | | | S | | | | T | | | | V | | | | V | | | | Q | | | P | | | M | | | Y | | | | D | | | I | | | GAVAMRLLTKYMNKEK--- | | | | | |  |
| *Bacillus licheniformis* | | | | | | | | | | | | | | | | | | | | | | | | | | | | | | | | | | | | | | | | | | | | | | | | | | | | | | | | | D | E | | K | | P | | T | | A | | V | | L | | | S | | | A | | | T | | | D | | | E | | | | M | | | | | A | | | L | | | G | | | | V | | | | I | | | | | | H | | | | A | | | | A | | | | | | | | | | | | | | | | Q | | | | | | | | | | | | | | | | | | | | | | | | | D | | | | | | | | | | | | K | | | | G | | | | L | | | A | | | | V | | | | P | | | D | | | | D | | | | L | | | | | E | | | | V | | | | I | | | | | G | | | F | | | D | | | N | | | | T | | | R | | | | L | | | | | S | | | | L | | | | M | | | | | V | | | R | | | | P | | | | Q | | | | L | | | | S | | | | T | | | | V | | | | V | | | | Q | | | P | | | T | | | Y | | | | D | | | I | | | GAVAMRLLTKLMNKEE--- | | | | | |  |
| *Bacillus megaterium* | | | | | | | | | | | | | | | | | | | | | | | | | | | | | | | | | | | | | | | | | | | | | | | | | | | | | | | | | D | E | | K | | P | | T | | A | | I | | F | | | V | | | G | | | T | | | D | | | E | | | | M | | | | | A | | | L | | | G | | | | V | | | | I | | | | | | H | | | | G | | | | A | | | | | | | | | | | | | | | | Q | | | | | | | | | | | | | | | | | | | | | | | | | D | | | | | | | | | | | | R | | | | G | | | | L | | | N | | | | V | | | | P | | | N | | | | D | | | | L | | | | | E | | | | I | | | | I | | | | | G | | | F | | | D | | | N | | | | T | | | R | | | | L | | | | | S | | | | T | | | | M | | | | | V | | | R | | | | P | | | | Q | | | | L | | | | T | | | | S | | | | V | | | | V | | | | Q | | | P | | | M | | | Y | | | | D | | | I | | | GAVAMRLLTKYMNKET--- | | | | | |  |
| *Bacillus subtilis* | | | | | | | | | | | | | | | | | | | | | | | | | | | | | | | | | | | | | | | | | | | | | | | | | | | | | | | | | D | K | | K | | P | | T | | A | | I | | L | | | S | | | A | | | T | | | D | | | E | | | | M | | | | | A | | | L | | | G | | | | I | | | | I | | | | | | H | | | | A | | | | A | | | | | | | | | | | | | | | | Q | | | | | | | | | | | | | | | | | | | | | | | | | D | | | | | | | | | | | | Q | | | | G | | | | L | | | S | | | | I | | | | P | | | E | | | | D | | | | L | | | | | D | | | | I | | | | I | | | | | G | | | F | | | D | | | N | | | | T | | | R | | | | L | | | | | S | | | | L | | | | M | | | | | V | | | R | | | | P | | | | Q | | | | L | | | | S | | | | T | | | | V | | | | V | | | | Q | | | P | | | T | | | Y | | | | D | | | I | | | GAVAMRLLTKLMNKEP--- | | | | | |  |
| *Enterococcus faecalis* | | | | | | | | | | | | | | | | | | | | | | | | | | | | | | | | | | | | | | | | | | | | | | | | | | | | | | | | | - | S | | G | | A | | T | | A | | A | | F | | | V | | | T | | | D | | | D | | | E | | | | L | | | | | A | | | I | | | G | | | | L | | | | L | | | | | | D | | | | G | | | | M | | | | | | | | | | | | | | | | L | | | | | | | | | | | | | | | | | | | | | | | | | D | | | | | | | | | | | | A | | | | G | | | | V | | | K | | | | V | | | | P | | | E | | | | E | | | | F | | | | | E | | | | I | | | | I | | | | | T | | | S | | | N | | | N | | | | S | | | L | | | | L | | | | | T | | | | E | | | | V | | | | | S | | | R | | | | P | | | | R | | | | L | | | | S | | | | S | | | | I | | | | T | | | | Q | | | P | | | L | | | Y | | | | D | | | I | | | GAVSMRLLTKLMNKEE--- | | | | | |  |
| *Lactobacillus. casei*subsp.*casei* | | | | | | | | | | | | | | | | | | | | | | | | | | | | | | | | | | | | | | | | | | | | | | | | | | | | | | | | | - | V | | G | | A | | T | | A | | V | | I | | | A | | | G | | | D | | | D | | | E | | | | L | | | | | A | | | V | | | G | | | | L | | | | L | | | | | | D | | | | G | | | | A | | | | | | | | | | | | | | | | I | | | | | | | | | | | | | | | | | | | | | | | | | D | | | | | | | | | | | | K | | | | G | | | | V | | | K | | | | V | | | | P | | | D | | | | D | | | | F | | | | | E | | | | I | | | | I | | | | | T | | | S | | | N | | | N | | | | T | | | K | | | | L | | | | | T | | | | E | | | | M | | | | | T | | | R | | | | P | | | | Q | | | | L | | | | T | | | | S | | | | I | | | | D | | | | Q | | | P | | | L | | | Y | | | | D | | | I | | | GAVAMRLLTKMMNKEE--- | | | | | |  |
| *Lactobacillus delbrueckii*subsp.*bulgaricus* | | | | | | | | | | | | | | | | | | | | | | | | | | | | | | | | | | | | | | | | | | | | | | | | | | | | | | | | | - | K | | G | | I | | N | | G | | V | | I | | | I | | | T | | | K | | | D | | | V | | | | S | | | | | S | | | V | | | G | | | | L | | | | L | | | | | | N | | | | S | | | | A | | | | | | | | | | | | | | | | L | | | | | | | | | | | | | | | | | | | | | | | | | D | | | | | | | | | | | | R | | | | G | | | | A | | | K | | | | V | | | | P | | | E | | | | D | | | | F | | | | | E | | | | I | | | | V | | | | | T | | | A | | | S | | | A | | | | T | | | Q | | | | I | | | | | A | | | | S | | | | V | | | | | V | | | R | | | | P | | | | A | | | | L | | | | T | | | | T | | | | I | | | | K | | | | Q | | | P | | | L | | | Y | | | | D | | | L | | | GAVAMRMLTKLMNDEG--- | | | | | |  |
| *Lactobacillus pentosus* | | | | | | | | | | | | | | | | | | | | | | | | | | | | | | | | | | | | | | | | | | | | | | | | | | | | | | | | | - | S | | G | | A | | T | | A | | V | | F | | | V | | | G | | | D | | | D | | | E | | | | M | | | | | A | | | A | | | G | | | | I | | | | I | | | | | | N | | | | A | | | | S | | | | | | | | | | | | | | | | M | | | | | | | | | | | | | | | | | | | | | | | | | E | | | | | | | | | | | | T | | | | G | | | | I | | | N | | | | V | | | | P | | | D | | | | D | | | | L | | | | | E | | | | V | | | | V | | | | | T | | | S | | | N | | | D | | | | T | | | I | | | | I | | | | | T | | | | Q | | | | I | | | | | T | | | R | | | | P | | | | A | | | | I | | | | T | | | | S | | | | I | | | | T | | | | Q | | | P | | | L | | | Y | | | | D | | | I | | | GAVAMRMLTKLMNDKE--- | | | | | |  |
| *Lactobacillus plantarum* | | | | | | | | | | | | | | | | | | | | | | | | | | | | | | | | | | | | | | | | | | | | | | | | | | | | | | | | | - | S | | G | | P | | T | | A | | V | | F | | | V | | | G | | | D | | | D | | | E | | | | M | | | | | A | | | A | | | G | | | | L | | | | I | | | | | | N | | | | A | | | | S | | | | | | | | | | | | | | | | M | | | | | | | | | | | | | | | | | | | | | | | | | E | | | | | | | | | | | | S | | | | G | | | | I | | | N | | | | V | | | | P | | | D | | | | D | | | | L | | | | | E | | | | V | | | | V | | | | | T | | | S | | | N | | | D | | | | T | | | I | | | | I | | | | | T | | | | Q | | | | I | | | | | T | | | R | | | | P | | | | A | | | | I | | | | T | | | | S | | | | I | | | | T | | | | Q | | | P | | | L | | | Y | | | | D | | | I | | | GAVAMRMLTKLMNDKE--- | | | | | |  |
| *Lactobacillus reuteri* | | | | | | | | | | | | | | | | | | | | | | | | | | | | | | | | | | | | | | | | | | | | | | | | | | | | | | | | | - | K | | G | | L | | K | | A | | T | | Y | | | V | | | T | | | D | | | D | | | S | | | | L | | | | | A | | | A | | | G | | | | L | | | | L | | | | | | N | | | | G | | | | L | | | | | | | | | | | | | | | | T | | | | | | | | | | | | | | | | | | | | | | | | | D | | | | | | | | | | | | A | | | | G | | | | I | | | S | | | | V | | | | P | | | D | | | | D | | | | F | | | | | E | | | | L | | | | I | | | | | S | | | S | | | N | | | D | | | | T | | | N | | | | Y | | | | | T | | | | K | | | | V | | | | | V | | | R | | | | P | | | | T | | | | I | | | | T | | | | S | | | | I | | | | T | | | | Q | | | P | | | L | | | Y | | | | D | | | L | | | GAISMRLLTKLMDGDDSN- | | | | | |  |
| *Lactobacillus sakei*subsp. *sakei* | | | | | | | | | | | | | | | | | | | | | | | | | | | | | | | | | | | | | | | | | | | | | | | | | | | | | | | | | - | I | | G | | A | | T | | A | | A | | V | | | V | | | S | | | D | | | D | | | E | | | | L | | | | | A | | | V | | | G | | | | V | | | | L | | | | | | N | | | | A | | | | A | | | | | | | | | | | | | | | | V | | | | | | | | | | | | | | | | | | | | | | | | | D | | | | | | | | | | | | A | | | | G | | | | V | | | N | | | | I | | | | P | | | T | | | | D | | | | F | | | | | E | | | | I | | | | V | | | | | T | | | S | | | N | | | N | | | | T | | | K | | | | L | | | | | T | | | | E | | | | M | | | | | V | | | R | | | | P | | | | K | | | | M | | | | T | | | | S | | | | I | | | | S | | | | Q | | | P | | | L | | | Y | | | | D | | | M | | | GAVGMRLLTKMMNKEE--- | | | | | |  |
| *Lactococcus lactis*AM2 | | | | | | | | | | | | | | | | | | | | | | | | | | | | | | | | | | | | | | | | | | | | | | | | | | | | | | | | | - | - | | - | | - | | - | | - | | - | | - | | | - | | | - | | | - | | | - | | | - | | | | - | | | | | - | | | - | | | - | | | | - | | | | - | | | | | | - | | | | - | | | | - | | | | | | | | | | | | | | | | - | | | | | | | | | | | | | | | | | | | | | | | | | - | | | | | | | | | | | | - | | | | - | | | | - | | | - | | | | - | | | | - | | | - | | | | - | | | | - | | | | | - | | | | - | | | | - | | | | | - | | | - | | | - | | | - | | | | - | | | - | | | | - | | | | | - | | | | - | | | | - | | | | | - | | | - | | | | - | | | | - | | | | - | | | | - | | | | - | | | | - | | | | - | | | | - | | | - | | | - | | | - | | | | - | | | - | | | ------------------- | | | | | |  |
| *Lactococcus lactis*LMG9447 | | | | | | | | | | | | | | | | | | | | | | | | | | | | | | | | | | | | | | | | | | | | | | | | | | | | | | | | | - | - | | - | | - | | - | | - | | - | | - | | | - | | | - | | | - | | | - | | | - | | | | - | | | | | - | | | - | | | - | | | | - | | | | - | | | | | | - | | | | - | | | | - | | | | | | | | | | | | | | | | - | | | | | | | | | | | | | | | | | | | | | | | | | - | | | | | | | | | | | | - | | | | - | | | | - | | | - | | | | - | | | | - | | | - | | | | - | | | | - | | | | | - | | | | - | | | | - | | | | | - | | | - | | | - | | | - | | | | - | | | - | | | | - | | | | | - | | | | - | | | | - | | | | | - | | | - | | | | - | | | | - | | | | - | | | | - | | | | - | | | | - | | | | - | | | | - | | | - | | | - | | | - | | | | - | | | - | | | ------------------- | | | | | |  |
| *Lactococcus lactis*P7304 | | | | | | | | | | | | | | | | | | | | | | | | | | | | | | | | | | | | | | | | | | | | | | | | | | | | | | | | | - | R | | G | | A | | T | | S | | A | | V | | | V | | | S | | | H | | | D | | | T | | | | V | | | | | A | | | V | | | G | | | | L | | | | L | | | | | | S | | | | A | | | | M | | | | | | | | | | | | | | | | M | | | | | | | | | | | | | | | | | | | | | | | | | D | | | | | | | | | | | | K | | | | E | | | | S | | | - | | | | - | | | | - | | | - | | | | - | | | | - | | | | | - | | | | - | | | | - | | | | | - | | | - | | | - | | | - | | | | - | | | - | | | | - | | | | | - | | | | - | | | | - | | | | | - | | | - | | | | - | | | | - | | | | - | | | | - | | | | - | | | | - | | | | - | | | | - | | | - | | | - | | | - | | | | - | | | - | | | ------------------- | | | | | |  |
| *Lactococcus lactis*subsp.*cremoris*MG1363 | | | | | | | | | | | | | | | | | | | | | | | | | | | | | | | | | | | | | | | | | | | | | | | | | | | | | | | | | - | R | | G | | A | | T | | S | | A | | V | | | V | | | S | | | H | | | D | | | T | | | | V | | | | | A | | | V | | | G | | | | L | | | | L | | | | | | S | | | | A | | | | M | | | | | | | | | | | | | | | | M | | | | | | | | | | | | | | | | | | | | | | | | | D | | | | | | | | | | | | K | | | | E | | | | V | | | K | | | | V | | | | P | | | E | | | | E | | | | F | | | | | E | | | | I | | | | I | | | | | S | | | G | | | A | | | N | | | | S | | | P | | | | I | | | | | T | | | | Q | | | | Y | | | | | T | | | Y | | | | P | | | | T | | | | L | | | | T | | | | S | | | | V | | | | N | | | | Q | | | P | | | L | | | Y | | | | D | | | L | | | GAVAMRLLTKLMLKED--- | | | | | |  |
| *Lactococcus lactis*subsp.*lactis*Il1403 | | | | | | | | | | | | | | | | | | | | | | | | | | | | | | | | | | | | | | | | | | | | | | | | | | | | | | | | | - | R | | G | | A | | T | | S | | A | | V | | | V | | | S | | | H | | | D | | | T | | | | V | | | | | A | | | V | | | G | | | | L | | | | L | | | | | | S | | | | A | | | | M | | | | | | | | | | | | | | | | M | | | | | | | | | | | | | | | | | | | | | | | | | D | | | | | | | | | | | | K | | | | G | | | | V | | | K | | | | V | | | | P | | | E | | | | D | | | | F | | | | | E | | | | I | | | | I | | | | | S | | | G | | | A | | | N | | | | S | | | P | | | | I | | | | | T | | | | Q | | | | Y | | | | | T | | | Y | | | | P | | | | T | | | | L | | | | T | | | | S | | | | V | | | | N | | | | Q | | | P | | | L | | | Y | | | | D | | | L | | | GAVAMRLLTKLMLKED--- | | | | | |  |
| *Lactococcus lactis*subsp.*lactis*KF147 | | | | | | | | | | | | | | | | | | | | | | | | | | | | | | | | | | | | | | | | | | | | | | | | | | | | | | | | | - | R | | G | | A | | T | | S | | A | | V | | | V | | | S | | | H | | | D | | | T | | | | V | | | | | A | | | V | | | G | | | | L | | | | L | | | | | | S | | | | A | | | | M | | | | | | | | | | | | | | | | M | | | | | | | | | | | | | | | | | | | | | | | | | D | | | | | | | | | | | | K | | | | G | | | | V | | | K | | | | V | | | | P | | | E | | | | D | | | | F | | | | | E | | | | I | | | | I | | | | | S | | | G | | | A | | | N | | | | S | | | P | | | | I | | | | | T | | | | Q | | | | Y | | | | | T | | | Y | | | | P | | | | T | | | | L | | | | T | | | | S | | | | V | | | | N | | | | Q | | | P | | | L | | | Y | | | | D | | | L | | | GAVAMRLLTKLMLKED--- | | | | | |  |
| *Lactococcus lactis*UC317 | | | | | | | | | | | | | | | | | | | | | | | | | | | | | | | | | | | | | | | | | | | | | | | | | | | | | | | | | - | - | | - | | - | | - | | - | | - | | - | | | - | | | - | | | - | | | - | | | - | | | | - | | | | | - | | | - | | | - | | | | - | | | | - | | | | | | - | | | | - | | | | - | | | | | | | | | | | | | | | | - | | | | | | | | | | | | | | | | | | | | | | | | | - | | | | | | | | | | | | - | | | | - | | | | - | | | - | | | | - | | | | - | | | - | | | | - | | | | - | | | | | - | | | | - | | | | - | | | | | - | | | - | | | - | | | - | | | | - | | | - | | | | - | | | | | - | | | | - | | | | - | | | | | - | | | - | | | | - | | | | - | | | | - | | | | - | | | | - | | | | - | | | | - | | | | - | | | - | | | - | | | - | | | | - | | | - | | | ------------------- | | | | | |  |
| *Listeria monocytogenes* | | | | | | | | | | | | | | | | | | | | | | | | | | | | | | | | | | | | | | | | | | | | | | | | | | | | | | | | | S | K | | K | | P | | N | | A | | V | | V | | | V | | | A | | | D | | | D | | | E | | | | L | | | | | A | | | I | | | G | | | | I | | | | L | | | | | | N | | | | A | | | | A | | | | | | | | | | | | | | | | L | | | | | | | | | | | | | | | | | | | | | | | | | D | | | | | | | | | | | | A | | | | G | | | | I | | | K | | | | V | | | | P | | | E | | | | D | | | | L | | | | | E | | | | V | | | | M | | | | | T | | | S | | | N | | | N | | | | T | | | K | | | | L | | | | | T | | | | L | | | | M | | | | | S | | | R | | | | P | | | | Q | | | | L | | | | S | | | | T | | | | I | | | | V | | | | Q | | | P | | | L | | | Y | | | | D | | | I | | | GAVAMRLLTKLMTSEE--- | | | | | |  |
| *Paenibacillus mucilaginosus* | | | | | | | | | | | | | | | | | | | | | | | | | | | | | | | | | | | | | | | | | | | | | | | | | | | | | | | | | D | E | | R | | P | | T | | A | | I | | F | | | A | | | A | | | T | | | D | | | E | | | | M | | | | | A | | | I | | | G | | | | A | | | | I | | | | | | H | | | | S | | | | L | | | | | | | | | | | | | | | | Q | | | | | | | | | | | | | | | | | | | | | | | | | D | | | | | | | | | | | | S | | | | G | | | | L | | | R | | | | V | | | | P | | | E | | | | E | | | | M | | | | | S | | | | V | | | | I | | | | | S | | | V | | | D | | | N | | | | I | | | R | | | | M | | | | | A | | | | S | | | | M | | | | | V | | | R | | | | P | | | | Q | | | | L | | | | T | | | | T | | | | V | | | | A | | | | Q | | | P | | | M | | | Y | | | | D | | | I | | | GAVAMRLLTKLMNKETKDA | | | | | |  |
| *Staphalococcus aureus*subsp.*aureus* | | | | | | | | | | | | | | | | | | | | | | | | | | | | | | | | | | | | | | | | | | | | | | | | | | | | | | | | | - | N | | L | | P | | D | | A | | I | | L | | | C | | | I | | | S | | | D | | | E | | | | E | | | | | A | | | I | | | G | | | | I | | | | M | | | | | | H | | | | S | | | | A | | | | | | | | | | | | | | | | M | | | | | | | | | | | | | | | | | | | | | | | | | D | | | | | | | | | | | | A | | | | G | | | | I | | | K | | | | V | | | | P | | | E | | | | E | | | | L | | | | | Q | | | | I | | | | I | | | | | S | | | F | | | N | | | N | | | | T | | | R | | | | L | | | | | V | | | | E | | | | M | | | | | V | | | R | | | | P | | | | Q | | | | L | | | | S | | | | S | | | | V | | | | I | | | | Q | | | P | | | L | | | Y | | | | D | | | I | | | GAVGMRLLTKYMNDEK--- | | | | | |  |
| *Staphalococcus xylosus* | | | | | | | | | | | | | | | | | | | | | | | | | | | | | | | | | | | | | | | | | | | | | | | | | | | | | | | | | - | A | | K | | P | | D | | A | | I | | L | | | S | | | I | | | S | | | D | | | E | | | | Q | | | | | A | | | I | | | G | | | | L | | | | V | | | | | | H | | | | A | | | | A | | | | | | | | | | | | | | | | Q | | | | | | | | | | | | | | | | | | | | | | | | | D | | | | | | | | | | | | A | | | | G | | | | V | | | N | | | | V | | | | P | | | N | | | | D | | | | L | | | | | Q | | | | I | | | | V | | | | | S | | | F | | | N | | | N | | | | T | | | R | | | | L | | | | | V | | | | E | | | | M | | | | | V | | | R | | | | P | | | | Q | | | | L | | | | S | | | | S | | | | V | | | | I | | | | Q | | | P | | | L | | | Y | | | | D | | | I | | | GAVGMRLLTKYMNEED--- | | | | | |  |
| *Staphalococcus bovis* | | | | | | | | | | | | | | | | | | | | | | | | | | | | | | | | | | | | | | | | | | | | | | | | | | | | | | | | | - | S | | G | | A | | T | | A | | A | | Y | | | V | | | A | | | E | | | D | | | E | | | | L | | | | | A | | | A | | | G | | | | L | | | | L | | | | | | N | | | | G | | | | L | | | | | | | | | | | | | | | | F | | | | | | | | | | | | | | | | | | | | | | | | | A | | | | | | | | | | | | A | | | | G | | | | K | | | K | | | | V | | | | P | | | E | | | | D | | | | F | | | | | E | | | | I | | | | L | | | | | T | | | S | | | N | | | D | | | | S | | | P | | | | I | | | | | T | | | | S | | | | Y | | | | | T | | | R | | | | P | | | | N | | | | L | | | | S | | | | S | | | | I | | | | S | | | | Q | | | P | | | V | | | Y | | | | D | | | L | | | GAVSMRMLTKIMNKEE--- | | | | | |  |
| *Streptococcus equi*subsp. *zooepidemicus* | | | | | | | | | | | | | | | | | | | | | | | | | | | | | | | | | | | | | | | | | | | | | | | | | | | | | | | | | - | S | | G | | A | | T | | A | | A | | Y | | | V | | | A | | | E | | | D | | | E | | | | L | | | | | A | | | A | | | G | | | | L | | | | L | | | | | | N | | | | G | | | | L | | | | | | | | | | | | | | | | F | | | | | | | | | | | | | | | | | | | | | | | | | E | | | | | | | | | | | | A | | | | G | | | | K | | | R | | | | V | | | | P | | | E | | | | D | | | | F | | | | | E | | | | I | | | | I | | | | | T | | | S | | | N | | | D | | | | S | | | P | | | | V | | | | | V | | | | Q | | | | Y | | | | | T | | | R | | | | P | | | | N | | | | L | | | | S | | | | S | | | | I | | | | S | | | | Q | | | P | | | V | | | Y | | | | D | | | L | | | GAVSMRMLTKIMNKEE--- | | | | | |  |
| *Streptococcus gallolyticus* | | | | | | | | | | | | | | | | | | | | | | | | | | | | | | | | | | | | | | | | | | | | | | | | | | | | | | | | | - | S | | G | | A | | T | | A | | A | | F | | | V | | | A | | | E | | | D | | | E | | | | L | | | | | A | | | A | | | G | | | | L | | | | L | | | | | | N | | | | G | | | | L | | | | | | | | | | | | | | | | F | | | | | | | | | | | | | | | | | | | | | | | | | E | | | | | | | | | | | | A | | | | G | | | | K | | | K | | | | V | | | | P | | | E | | | | D | | | | F | | | | | E | | | | I | | | | I | | | | | T | | | S | | | N | | | D | | | | S | | | P | | | | I | | | | | T | | | | S | | | | Y | | | | | T | | | R | | | | P | | | | N | | | | L | | | | S | | | | S | | | | I | | | | S | | | | Q | | | P | | | V | | | Y | | | | D | | | L | | | GAVSMRMLTKIMNKEE--- | | | | | |  |
| *Streptococcus mutans* | | | | | | | | | | | | | | | | | | | | | | | | | | | | | | | | | | | | | | | | | | | | | | | | | | | | | | | | | - | S | | G | | A | | T | | A | | A | | Y | | | V | | | A | | | E | | | D | | | E | | | | L | | | | | A | | | A | | | G | | | | L | | | | L | | | | | | N | | | | G | | | | L | | | | | | | | | | | | | | | | F | | | | | | | | | | | | | | | | | | | | | | | | | A | | | | | | | | | | | | A | | | | G | | | | K | | | K | | | | V | | | | P | | | E | | | | D | | | | F | | | | | E | | | | I | | | | I | | | | | T | | | S | | | N | | | D | | | | S | | | T | | | | I | | | | | A | | | | L | | | | Y | | | | | T | | | R | | | | P | | | | N | | | | M | | | | T | | | | S | | | | I | | | | S | | | | Q | | | P | | | I | | | Y | | | | D | | | L | | | GAVAMRMLTKIMNKEE--- | | | | | |  |
| *Streptococcus thermophilus* | | | | | | | | | | | | | | | | | | | | | | | | | | | | | | | | | | | | | | | | | | | | | | | | | | | | | | | | | - | S | | G | | A | | T | | A | | A | | Y | | | V | | | G | | | E | | | D | | | E | | | | L | | | | | A | | | A | | | G | | | | L | | | | L | | | | | | N | | | | G | | | | L | | | | | | | | | | | | | | | | F | | | | | | | | | | | | | | | | | | | | | | | | | A | | | | | | | | | | | | A | | | | G | | | | K | | | S | | | | V | | | | P | | | E | | | | D | | | | F | | | | | E | | | | I | | | | I | | | | | T | | | S | | | N | | | D | | | | S | | | P | | | | V | | | | | T | | | | S | | | | Y | | | | | T | | | R | | | | P | | | | N | | | | L | | | | S | | | | S | | | | I | | | | N | | | | H | | | P | | | L | | | Y | | | | D | | | L | | | GAVSMRMLTKIMHKEE--- | | | | | |  |
|  | | | | | | | | | | | | | | | | | | | | | | | | | | | | | | | | | | | | | | | | | | | | | | | | | | | | | | | | | | | | | | | | | | | | | | | | | | | | | | | | | | | | | | | | | | | | | | | | | | | | | | | | | | | | | | | | | | | | | | | | | | | | | | | | | | | | | | | | | | | | | | | | | | | | | | | | | | | | | | | | | | | | | | | | | | | | | | | | | | | | | | | | | | | | | | | | | | | | | | | | | | | | | | | | | | | | | | | | | | | | | | | | | | | | | | | | | | | | | | | | | | | | | | | | | | | | | | | | | | | | | | | | | | | | | | | | | | | | | | | | | | | | | | | | | | | | | | | | | | | | | | | | | | | | | | | | | | |  | | | | | |  |
| *Bacillus amyloliquefaciens* | | | | | | | | | | | | | | | | | | | | | | | | | | | | | | | | | | | | | | | | | | | | | | | | | | | | | | | | | V | E | | E | | Q | | I | | V | | E | | | L | | | P | | | H | | | R | | | I | | | E | | | | L | | | R | | | Q | | | S | | | | T | | | | K | | | | S | | | | | - | | | | - | | | |  | | | | | | | | | | | | | | | | | | | | | | | | | | | | | | | | | | | | | | | | | | | | | | |  |  |  |  |  |  |  |  |  |  |  |  |  |  |  |  |  |  |  |  |  |  |  |  |  |  |  |  |  |  |  |  |  |  |  |  |  |  |  |  |  |  |  |  |  |  |  |  |  |  |  |  |  |  |  |  |  |  |  |  |  |  |  |  |  |  |  |  |  |  |  |  |  |  |  |  |  |  |  |  |  |  |  |  |  |  |  |  |  |  |  |  |  |  |  |  |  |  |  |  |  |  |  |  |  |  |  |  |  |  |  |  |  |  |  |  |  |  |  |  |  |  |  |  |  |  |  |  |  |  |  |  |  |  |  |  |  |  |  |  |  |  |  |  |  |  |  |  |  |  |  |
| *Bacillus anthracis* | | | | | | | | | | | | | | | | | | | | | | | | | | | | | | | | | | | | | | | | | | | | | | | | | | | | | | | | | V | E | | D | | H | | T | | V | | I | | | L | | | P | | | H | | | R | | | I | | | Q | | | | F | | | R | | | D | | | S | | | | T | | | | K | | | | - | | | | | - | | | | - | | | |  | | | | | | | | | | | | | | | | | | | | | | | | | | | | | | | | | | | | | | | | | | | | | | |  |  |  |  |  |  |  |  |  |  |  |  |  |  |  |  |  |  |  |  |  |  |  |  |  |  |  |  |  |  |  |  |  |  |  |  |  |  |  |  |  |  |  |  |  |  |  |  |  |  |  |  |  |  |  |  |  |  |  |  |  |  |  |  |  |  |  |  |  |  |  |  |  |  |  |  |  |  |  |  |  |  |  |  |  |  |  |  |  |  |  |  |  |  |  |  |  |  |  |  |  |  |  |  |  |  |  |  |  |  |  |  |  |  |  |  |  |  |  |  |  |  |  |  |  |  |  |  |  |  |  |  |  |  |  |  |  |  |  |  |  |  |  |  |  |  |  |  |  |  |  |
| *Bacillus licheniformis* | | | | | | | | | | | | | | | | | | | | | | | | | | | | | | | | | | | | | | | | | | | | | | | | | | | | | | | | | V | E | | E | | H | | I | | V | | Q | | | L | | | P | | | H | | | R | | | I | | | E | | | | L | | | R | | | Q | | | S | | | | T | | | | K | | | | - | | | | | - | | | | - | | | |  | | | | | | | | | | | | | | | | | | | | | | | | | | | | | | | | | | | | | | | | | | | | | | |  |  |  |  |  |  |  |  |  |  |  |  |  |  |  |  |  |  |  |  |  |  |  |  |  |  |  |  |  |  |  |  |  |  |  |  |  |  |  |  |  |  |  |  |  |  |  |  |  |  |  |  |  |  |  |  |  |  |  |  |  |  |  |  |  |  |  |  |  |  |  |  |  |  |  |  |  |  |  |  |  |  |  |  |  |  |  |  |  |  |  |  |  |  |  |  |  |  |  |  |  |  |  |  |  |  |  |  |  |  |  |  |  |  |  |  |  |  |  |  |  |  |  |  |  |  |  |  |  |  |  |  |  |  |  |  |  |  |  |  |  |  |  |  |  |  |  |  |  |  |  |
| *Bacillus megaterium* | | | | | | | | | | | | | | | | | | | | | | | | | | | | | | | | | | | | | | | | | | | | | | | | | | | | | | | | | V | D | | S | | S | | I | | V | | Q | | | L | | | P | | | H | | | R | | | I | | | E | | | | F | | | R | | | Q | | | S | | | | T | | | | K | | | | - | | | | | - | | | | - | | | |  | | | | | | | | | | | | | | | | | | | | | | | | | | | | | | | | | | | | | | | | | | | | | | |  |  |  |  |  |  |  |  |  |  |  |  |  |  |  |  |  |  |  |  |  |  |  |  |  |  |  |  |  |  |  |  |  |  |  |  |  |  |  |  |  |  |  |  |  |  |  |  |  |  |  |  |  |  |  |  |  |  |  |  |  |  |  |  |  |  |  |  |  |  |  |  |  |  |  |  |  |  |  |  |  |  |  |  |  |  |  |  |  |  |  |  |  |  |  |  |  |  |  |  |  |  |  |  |  |  |  |  |  |  |  |  |  |  |  |  |  |  |  |  |  |  |  |  |  |  |  |  |  |  |  |  |  |  |  |  |  |  |  |  |  |  |  |  |  |  |  |  |  |  |  |
| *Bacillus subtilis* | | | | | | | | | | | | | | | | | | | | | | | | | | | | | | | | | | | | | | | | | | | | | | | | | | | | | | | | | V | E | | E | | H | | I | | V | | E | | | L | | | P | | | H | | | R | | | I | | | E | | | | L | | | R | | | K | | | S | | | | T | | | | K | | | | S | | | | | - | | | | - | | | |  | | | | | | | | | | | | | | | | | | | | | | | | | | | | | | | | | | | | | | | | | | | | | | |  |  |  |  |  |  |  |  |  |  |  |  |  |  |  |  |  |  |  |  |  |  |  |  |  |  |  |  |  |  |  |  |  |  |  |  |  |  |  |  |  |  |  |  |  |  |  |  |  |  |  |  |  |  |  |  |  |  |  |  |  |  |  |  |  |  |  |  |  |  |  |  |  |  |  |  |  |  |  |  |  |  |  |  |  |  |  |  |  |  |  |  |  |  |  |  |  |  |  |  |  |  |  |  |  |  |  |  |  |  |  |  |  |  |  |  |  |  |  |  |  |  |  |  |  |  |  |  |  |  |  |  |  |  |  |  |  |  |  |  |  |  |  |  |  |  |  |  |  |  |  |
| *Enterococcus faecalis* | | | | | | | | | | | | | | | | | | | | | | | | | | | | | | | | | | | | | | | | | | | | | | | | | | | | | | | | | I | E | | E | | K | | T | | V | | V | | | L | | | P | | | Y | | | G | | | I | | | D | | | | Q | | | K | | | G | | | S | | | | T | | | | K | | | | - | | | | | - | | | | - | | | |  | | | | | | | | | | | | | | | | | | | | | | | | | | | | | | | | | | | | | | | | | | | | | | |  |  |  |  |  |  |  |  |  |  |  |  |  |  |  |  |  |  |  |  |  |  |  |  |  |  |  |  |  |  |  |  |  |  |  |  |  |  |  |  |  |  |  |  |  |  |  |  |  |  |  |  |  |  |  |  |  |  |  |  |  |  |  |  |  |  |  |  |  |  |  |  |  |  |  |  |  |  |  |  |  |  |  |  |  |  |  |  |  |  |  |  |  |  |  |  |  |  |  |  |  |  |  |  |  |  |  |  |  |  |  |  |  |  |  |  |  |  |  |  |  |  |  |  |  |  |  |  |  |  |  |  |  |  |  |  |  |  |  |  |  |  |  |  |  |  |  |  |  |  |  |
| *Lactobacillus. casei*subsp.*casei* | | | | | | | | | | | | | | | | | | | | | | | | | | | | | | | | | | | | | | | | | | | | | | | | | | | | | | | | | I | E | | E | | K | | T | | V | | M | | | L | | | G | | | F | | | D | | | I | | | L | | | | K | | | R | | | G | | | S | | | | T | | | | K | | | | - | | | | | - | | | | - | | | |  | | | | | | | | | | | | | | | | | | | | | | | | | | | | | | | | | | | | | | | | | | | | | | |  |  |  |  |  |  |  |  |  |  |  |  |  |  |  |  |  |  |  |  |  |  |  |  |  |  |  |  |  |  |  |  |  |  |  |  |  |  |  |  |  |  |  |  |  |  |  |  |  |  |  |  |  |  |  |  |  |  |  |  |  |  |  |  |  |  |  |  |  |  |  |  |  |  |  |  |  |  |  |  |  |  |  |  |  |  |  |  |  |  |  |  |  |  |  |  |  |  |  |  |  |  |  |  |  |  |  |  |  |  |  |  |  |  |  |  |  |  |  |  |  |  |  |  |  |  |  |  |  |  |  |  |  |  |  |  |  |  |  |  |  |  |  |  |  |  |  |  |  |  |  |
| *Lactobacillus delbrueckii*subsp.*bulgaricus* | | | | | | | | | | | | | | | | | | | | | | | | | | | | | | | | | | | | | | | | | | | | | | | | | | | | | | | | | L | E | | D | | K | | H | | I | | V | | | L | | | P | | | Y | | | E | | | L | | | I | | | | K | | | K | | | Q | | | S | | | | T | | | | L | | | | N | | | | | K | | | | - | | | |  | | | | | | | | | | | | | | | | | | | | | | | | | | | | | | | | | | | | | | | | | | | | | | |  |  |  |  |  |  |  |  |  |  |  |  |  |  |  |  |  |  |  |  |  |  |  |  |  |  |  |  |  |  |  |  |  |  |  |  |  |  |  |  |  |  |  |  |  |  |  |  |  |  |  |  |  |  |  |  |  |  |  |  |  |  |  |  |  |  |  |  |  |  |  |  |  |  |  |  |  |  |  |  |  |  |  |  |  |  |  |  |  |  |  |  |  |  |  |  |  |  |  |  |  |  |  |  |  |  |  |  |  |  |  |  |  |  |  |  |  |  |  |  |  |  |  |  |  |  |  |  |  |  |  |  |  |  |  |  |  |  |  |  |  |  |  |  |  |  |  |  |  |  |  |
| *Lactobacillus pentosus* | | | | | | | | | | | | | | | | | | | | | | | | | | | | | | | | | | | | | | | | | | | | | | | | | | | | | | | | | L | D | | E | | K | | T | | V | | T | | | L | | | P | | | Y | | | G | | | I | | | V | | | | R | | | R | | | G | | | S | | | | T | | | | K | | | | S | | | | | A | | | | D | | | |  | | | | | | | | | | | | | | | | | | | | | | | | | | | | | | | | | | | | | | | | | | | | | | |  |  |  |  |  |  |  |  |  |  |  |  |  |  |  |  |  |  |  |  |  |  |  |  |  |  |  |  |  |  |  |  |  |  |  |  |  |  |  |  |  |  |  |  |  |  |  |  |  |  |  |  |  |  |  |  |  |  |  |  |  |  |  |  |  |  |  |  |  |  |  |  |  |  |  |  |  |  |  |  |  |  |  |  |  |  |  |  |  |  |  |  |  |  |  |  |  |  |  |  |  |  |  |  |  |  |  |  |  |  |  |  |  |  |  |  |  |  |  |  |  |  |  |  |  |  |  |  |  |  |  |  |  |  |  |  |  |  |  |  |  |  |  |  |  |  |  |  |  |  |  |
| *Lactobacillus plantarum* | | | | | | | | | | | | | | | | | | | | | | | | | | | | | | | | | | | | | | | | | | | | | | | | | | | | | | | | | L | D | | E | | K | | T | | V | | T | | | L | | | P | | | Y | | | G | | | I | | | V | | | | R | | | R | | | G | | | S | | | | T | | | | K | | | | S | | | | | A | | | | D | | | |  | | | | | | | | | | | | | | | | | | | | | | | | | | | | | | | | | | | | | | | | | | | | | | |  |  |  |  |  |  |  |  |  |  |  |  |  |  |  |  |  |  |  |  |  |  |  |  |  |  |  |  |  |  |  |  |  |  |  |  |  |  |  |  |  |  |  |  |  |  |  |  |  |  |  |  |  |  |  |  |  |  |  |  |  |  |  |  |  |  |  |  |  |  |  |  |  |  |  |  |  |  |  |  |  |  |  |  |  |  |  |  |  |  |  |  |  |  |  |  |  |  |  |  |  |  |  |  |  |  |  |  |  |  |  |  |  |  |  |  |  |  |  |  |  |  |  |  |  |  |  |  |  |  |  |  |  |  |  |  |  |  |  |  |  |  |  |  |  |  |  |  |  |  |  |
| *Lactobacillus reuteri* | | | | | | | | | | | | | | | | | | | | | | | | | | | | | | | | | | | | | | | | | | | | | | | | | | | | | | | | | D | D | | E | | K | | N | | V | | I | | | L | | | D | | | H | | | G | | | F | | | V | | | | E | | | R | | | Q | | | S | | | | T | | | | R | | | | K | | | | | - | | | | - | | | |  | | | | | | | | | | | | | | | | | | | | | | | | | | | | | | | | | | | | | | | | | | | | | | |  |  |  |  |  |  |  |  |  |  |  |  |  |  |  |  |  |  |  |  |  |  |  |  |  |  |  |  |  |  |  |  |  |  |  |  |  |  |  |  |  |  |  |  |  |  |  |  |  |  |  |  |  |  |  |  |  |  |  |  |  |  |  |  |  |  |  |  |  |  |  |  |  |  |  |  |  |  |  |  |  |  |  |  |  |  |  |  |  |  |  |  |  |  |  |  |  |  |  |  |  |  |  |  |  |  |  |  |  |  |  |  |  |  |  |  |  |  |  |  |  |  |  |  |  |  |  |  |  |  |  |  |  |  |  |  |  |  |  |  |  |  |  |  |  |  |  |  |  |  |  |
| *Lactobacillus sakei*subsp. *sakei* | | | | | | | | | | | | | | | | | | | | | | | | | | | | | | | | | | | | | | | | | | | | | | | | | | | | | | | | | I | D | | D | | K | | T | | I | | L | | | L | | | P | | | Y | | | S | | | I | | | V | | | | D | | | R | | | Q | | | S | | | | T | | | | K | | | | - | | | | | - | | | | - | | | |  | | | | | | | | | | | | | | | | | | | | | | | | | | | | | | | | | | | | | | | | | | | | | | |  |  |  |  |  |  |  |  |  |  |  |  |  |  |  |  |  |  |  |  |  |  |  |  |  |  |  |  |  |  |  |  |  |  |  |  |  |  |  |  |  |  |  |  |  |  |  |  |  |  |  |  |  |  |  |  |  |  |  |  |  |  |  |  |  |  |  |  |  |  |  |  |  |  |  |  |  |  |  |  |  |  |  |  |  |  |  |  |  |  |  |  |  |  |  |  |  |  |  |  |  |  |  |  |  |  |  |  |  |  |  |  |  |  |  |  |  |  |  |  |  |  |  |  |  |  |  |  |  |  |  |  |  |  |  |  |  |  |  |  |  |  |  |  |  |  |  |  |  |  |  |
| *Lactococcus lactis*AM2 | | | | | | | | | | | | | | | | | | | | | | | | | | | | | | | | | | | | | | | | | | | | | | | | | | | | | | | | | - | - | | - | | - | | - | | - | | - | | | - | | | - | | | - | | | - | | | - | | | - | | | | - | | | - | | | - | | | - | | | | - | | | | - | | | | - | | | | | - | | | | - | | | |  | | | | | | | | | | | | | | | | | | | | | | | | | | | | | | | | | | | | | | | | | | | | | | |  |  |  |  |  |  |  |  |  |  |  |  |  |  |  |  |  |  |  |  |  |  |  |  |  |  |  |  |  |  |  |  |  |  |  |  |  |  |  |  |  |  |  |  |  |  |  |  |  |  |  |  |  |  |  |  |  |  |  |  |  |  |  |  |  |  |  |  |  |  |  |  |  |  |  |  |  |  |  |  |  |  |  |  |  |  |  |  |  |  |  |  |  |  |  |  |  |  |  |  |  |  |  |  |  |  |  |  |  |  |  |  |  |  |  |  |  |  |  |  |  |  |  |  |  |  |  |  |  |  |  |  |  |  |  |  |  |  |  |  |  |  |  |  |  |  |  |  |  |  |  |
| *Lactococcus lactis*LMG9447 | | | | | | | | | | | | | | | | | | | | | | | | | | | | | | | | | | | | | | | | | | | | | | | | | | | | | | | | | - | - | | - | | - | | - | | - | | - | | | - | | | - | | | - | | | - | | | - | | | - | | | | - | | | - | | | - | | | - | | | | - | | | | - | | | | - | | | | | - | | | | - | | | |  | | | | | | | | | | | | | | | | | | | | | | | | | | | | | | | | | | | | | | | | | | | | | | |  |  |  |  |  |  |  |  |  |  |  |  |  |  |  |  |  |  |  |  |  |  |  |  |  |  |  |  |  |  |  |  |  |  |  |  |  |  |  |  |  |  |  |  |  |  |  |  |  |  |  |  |  |  |  |  |  |  |  |  |  |  |  |  |  |  |  |  |  |  |  |  |  |  |  |  |  |  |  |  |  |  |  |  |  |  |  |  |  |  |  |  |  |  |  |  |  |  |  |  |  |  |  |  |  |  |  |  |  |  |  |  |  |  |  |  |  |  |  |  |  |  |  |  |  |  |  |  |  |  |  |  |  |  |  |  |  |  |  |  |  |  |  |  |  |  |  |  |  |  |  |
| *Lactococcus lactis*P7304 | | | | | | | | | | | | | | | | | | | | | | | | | | | | | | | | | | | | | | | | | | | | | | | | | | | | | | | | | - | - | | - | | - | | - | | - | | - | | | - | | | - | | | - | | | - | | | - | | | - | | | | - | | | - | | | - | | | - | | | | - | | | | - | | | | - | | | | | - | | | | - | | | |  | | | | | | | | | | | | | | | | | | | | | | | | | | | | | | | | | | | | | | | | | | | | | | |  |  |  |  |  |  |  |  |  |  |  |  |  |  |  |  |  |  |  |  |  |  |  |  |  |  |  |  |  |  |  |  |  |  |  |  |  |  |  |  |  |  |  |  |  |  |  |  |  |  |  |  |  |  |  |  |  |  |  |  |  |  |  |  |  |  |  |  |  |  |  |  |  |  |  |  |  |  |  |  |  |  |  |  |  |  |  |  |  |  |  |  |  |  |  |  |  |  |  |  |  |  |  |  |  |  |  |  |  |  |  |  |  |  |  |  |  |  |  |  |  |  |  |  |  |  |  |  |  |  |  |  |  |  |  |  |  |  |  |  |  |  |  |  |  |  |  |  |  |  |  |
| *Lactococcus lactis*subsp.*cremoris*MG1363 | | | | | | | | | | | | | | | | | | | | | | | | | | | | | | | | | | | | | | | | | | | | | | | | | | | | | | | | | V | E | | Q | | N | | Q | | L | | V | | | L | | | D | | | H | | | E | | | I | | | I | | | | S | | | R | | | R | | | S | | | | T | | | | K | | | | - | | | | | - | | | | - | | | |  | | | | | | | | | | | | | | | | | | | | | | | | | | | | | | | | | | | | | | | | | | | | | | |  |  |  |  |  |  |  |  |  |  |  |  |  |  |  |  |  |  |  |  |  |  |  |  |  |  |  |  |  |  |  |  |  |  |  |  |  |  |  |  |  |  |  |  |  |  |  |  |  |  |  |  |  |  |  |  |  |  |  |  |  |  |  |  |  |  |  |  |  |  |  |  |  |  |  |  |  |  |  |  |  |  |  |  |  |  |  |  |  |  |  |  |  |  |  |  |  |  |  |  |  |  |  |  |  |  |  |  |  |  |  |  |  |  |  |  |  |  |  |  |  |  |  |  |  |  |  |  |  |  |  |  |  |  |  |  |  |  |  |  |  |  |  |  |  |  |  |  |  |  |  |
| *Lactococcus lactis*subsp.*lactis*Il1403 | | | | | | | | | | | | | | | | | | | | | | | | | | | | | | | | | | | | | | | | | | | | | | | | | | | | | | | | | V | E | | Q | | N | | Q | | L | | V | | | L | | | D | | | H | | | E | | | I | | | F | | | | S | | | R | | | R | | | S | | | | T | | | | K | | | | - | | | | | - | | | | - | | | |  | | | | | | | | | | | | | | | | | | | | | | | | | | | | | | | | | | | | | | | | | | | | | | |  |  |  |  |  |  |  |  |  |  |  |  |  |  |  |  |  |  |  |  |  |  |  |  |  |  |  |  |  |  |  |  |  |  |  |  |  |  |  |  |  |  |  |  |  |  |  |  |  |  |  |  |  |  |  |  |  |  |  |  |  |  |  |  |  |  |  |  |  |  |  |  |  |  |  |  |  |  |  |  |  |  |  |  |  |  |  |  |  |  |  |  |  |  |  |  |  |  |  |  |  |  |  |  |  |  |  |  |  |  |  |  |  |  |  |  |  |  |  |  |  |  |  |  |  |  |  |  |  |  |  |  |  |  |  |  |  |  |  |  |  |  |  |  |  |  |  |  |  |  |  |
| *Lactococcus lactis*subsp.*lactis*KF147 | | | | | | | | | | | | | | | | | | | | | | | | | | | | | | | | | | | | | | | | | | | | | | | | | | | | | | | | | V | E | | Q | | N | | Q | | L | | V | | | L | | | D | | | H | | | E | | | I | | | F | | | | S | | | R | | | R | | | S | | | | T | | | | K | | | | - | | | | | - | | | | - | | | |  | | | | | | | | | | | | | | | | | | | | | | | | | | | | | | | | | | | | | | | | | | | | | | |  |  |  |  |  |  |  |  |  |  |  |  |  |  |  |  |  |  |  |  |  |  |  |  |  |  |  |  |  |  |  |  |  |  |  |  |  |  |  |  |  |  |  |  |  |  |  |  |  |  |  |  |  |  |  |  |  |  |  |  |  |  |  |  |  |  |  |  |  |  |  |  |  |  |  |  |  |  |  |  |  |  |  |  |  |  |  |  |  |  |  |  |  |  |  |  |  |  |  |  |  |  |  |  |  |  |  |  |  |  |  |  |  |  |  |  |  |  |  |  |  |  |  |  |  |  |  |  |  |  |  |  |  |  |  |  |  |  |  |  |  |  |  |  |  |  |  |  |  |  |  |
| *Lactococcus lactis*UC317 | | | | | | | | | | | | | | | | | | | | | | | | | | | | | | | | | | | | | | | | | | | | | | | | | | | | | | | | | - | - | | - | | - | | - | | - | | - | | | - | | | - | | | - | | | - | | | - | | | - | | | | - | | | - | | | - | | | - | | | | - | | | | - | | | | - | | | | | - | | | | - | | | |  | | | | | | | | | | | | | | | | | | | | | | | | | | | | | | | | | | | | | | | | | | | | | | |  |  |  |  |  |  |  |  |  |  |  |  |  |  |  |  |  |  |  |  |  |  |  |  |  |  |  |  |  |  |  |  |  |  |  |  |  |  |  |  |  |  |  |  |  |  |  |  |  |  |  |  |  |  |  |  |  |  |  |  |  |  |  |  |  |  |  |  |  |  |  |  |  |  |  |  |  |  |  |  |  |  |  |  |  |  |  |  |  |  |  |  |  |  |  |  |  |  |  |  |  |  |  |  |  |  |  |  |  |  |  |  |  |  |  |  |  |  |  |  |  |  |  |  |  |  |  |  |  |  |  |  |  |  |  |  |  |  |  |  |  |  |  |  |  |  |  |  |  |  |  |
| *Listeria monocytogenes* | | | | | | | | | | | | | | | | | | | | | | | | | | | | | | | | | | | | | | | | | | | | | | | | | | | | | | | | | V | D | | E | | K | | T | | V | | I | | | L | | | P | | | H | | | S | | | E | | | K | | | | L | | | R | | | G | | | T | | | | T | | | | K | | | | E | | | | | K | | | | K | | | |  | | | | | | | | | | | | | | | | | | | | | | | | | | | | | | | | | | | | | | | | | | | | | | |  |  |  |  |  |  |  |  |  |  |  |  |  |  |  |  |  |  |  |  |  |  |  |  |  |  |  |  |  |  |  |  |  |  |  |  |  |  |  |  |  |  |  |  |  |  |  |  |  |  |  |  |  |  |  |  |  |  |  |  |  |  |  |  |  |  |  |  |  |  |  |  |  |  |  |  |  |  |  |  |  |  |  |  |  |  |  |  |  |  |  |  |  |  |  |  |  |  |  |  |  |  |  |  |  |  |  |  |  |  |  |  |  |  |  |  |  |  |  |  |  |  |  |  |  |  |  |  |  |  |  |  |  |  |  |  |  |  |  |  |  |  |  |  |  |  |  |  |  |  |  |
| *Paenibacillus mucilaginosus* | | | | | | | | | | | | | | | | | | | | | | | | | | | | | | | | | | | | | | | | | | | | | | | | | | | | | | | | | S | E | | L | | T | | Q | | V | | I | | | L | | | P | | | H | | | E | | | V | | | I | | | | H | | | R | | | N | | | S | | | | V | | | | A | | | | Q | | | | | R | | | | S | | | |  | | | | | | | | | | | | | | | | | | | | | | | | | | | | | | | | | | | | | | | | | | | | | | |  |  |  |  |  |  |  |  |  |  |  |  |  |  |  |  |  |  |  |  |  |  |  |  |  |  |  |  |  |  |  |  |  |  |  |  |  |  |  |  |  |  |  |  |  |  |  |  |  |  |  |  |  |  |  |  |  |  |  |  |  |  |  |  |  |  |  |  |  |  |  |  |  |  |  |  |  |  |  |  |  |  |  |  |  |  |  |  |  |  |  |  |  |  |  |  |  |  |  |  |  |  |  |  |  |  |  |  |  |  |  |  |  |  |  |  |  |  |  |  |  |  |  |  |  |  |  |  |  |  |  |  |  |  |  |  |  |  |  |  |  |  |  |  |  |  |  |  |  |  |  |
| *Staphalococcus aureus*subsp.*aureus* | | | | | | | | | | | | | | | | | | | | | | | | | | | | | | | | | | | | | | | | | | | | | | | | | | | | | | | | | I | E | | E | | P | | N | | V | | V | | | L | | | P | | | H | | | R | | | I | | | E | | | | Y | | | R | | | G | | | T | | | | T | | | | K | | | | - | | | | | - | | | | - | | | |  | | | | | | | | | | | | | | | | | | | | | | | | | | | | | | | | | | | | | | | | | | | | | | |  |  |  |  |  |  |  |  |  |  |  |  |  |  |  |  |  |  |  |  |  |  |  |  |  |  |  |  |  |  |  |  |  |  |  |  |  |  |  |  |  |  |  |  |  |  |  |  |  |  |  |  |  |  |  |  |  |  |  |  |  |  |  |  |  |  |  |  |  |  |  |  |  |  |  |  |  |  |  |  |  |  |  |  |  |  |  |  |  |  |  |  |  |  |  |  |  |  |  |  |  |  |  |  |  |  |  |  |  |  |  |  |  |  |  |  |  |  |  |  |  |  |  |  |  |  |  |  |  |  |  |  |  |  |  |  |  |  |  |  |  |  |  |  |  |  |  |  |  |  |  |
| *Staphalococcus xylosus* | | | | | | | | | | | | | | | | | | | | | | | | | | | | | | | | | | | | | | | | | | | | | | | | | | | | | | | | | I | D | | E | | P | | N | | V | | I | | | L | | | P | | | H | | | R | | | I | | | E | | | | Y | | | R | | | G | | | T | | | | T | | | | K | | | | - | | | | | - | | | | - | | | |  | | | | | | | | | | | | | | | | | | | | | | | | | | | | | | | | | | | | | | | | | | | | | | |  |  |  |  |  |  |  |  |  |  |  |  |  |  |  |  |  |  |  |  |  |  |  |  |  |  |  |  |  |  |  |  |  |  |  |  |  |  |  |  |  |  |  |  |  |  |  |  |  |  |  |  |  |  |  |  |  |  |  |  |  |  |  |  |  |  |  |  |  |  |  |  |  |  |  |  |  |  |  |  |  |  |  |  |  |  |  |  |  |  |  |  |  |  |  |  |  |  |  |  |  |  |  |  |  |  |  |  |  |  |  |  |  |  |  |  |  |  |  |  |  |  |  |  |  |  |  |  |  |  |  |  |  |  |  |  |  |  |  |  |  |  |  |  |  |  |  |  |  |  |  |
| *Staphalococcus bovis* | | | | | | | | | | | | | | | | | | | | | | | | | | | | | | | | | | | | | | | | | | | | | | | | | | | | | | | | | L | E | | E | | K | | E | | I | | I | | | L | | | N | | | H | | | G | | | L | | | K | | | | L | | | R | | | G | | | T | | | | T | | | | R | | | | Q | | | | | - | | | | - | | | |  | | | | | | | | | | | | | | | | | | | | | | | | | | | | | | | | | | | | | | | | | | | | | | |  |  |  |  |  |  |  |  |  |  |  |  |  |  |  |  |  |  |  |  |  |  |  |  |  |  |  |  |  |  |  |  |  |  |  |  |  |  |  |  |  |  |  |  |  |  |  |  |  |  |  |  |  |  |  |  |  |  |  |  |  |  |  |  |  |  |  |  |  |  |  |  |  |  |  |  |  |  |  |  |  |  |  |  |  |  |  |  |  |  |  |  |  |  |  |  |  |  |  |  |  |  |  |  |  |  |  |  |  |  |  |  |  |  |  |  |  |  |  |  |  |  |  |  |  |  |  |  |  |  |  |  |  |  |  |  |  |  |  |  |  |  |  |  |  |  |  |  |  |  |  |
| *Streptococcus equi*subsp. *zooepidemicus* | | | | | | | | | | | | | | | | | | | | | | | | | | | | | | | | | | | | | | | | | | | | | | | | | | | | | | | | | L | E | | E | | K | | E | | I | | L | | | L | | | N | | | H | | | G | | | I | | | K | | | | K | | | R | | | G | | | T | | | | T | | | | K | | | | - | | | | | - | | | | - | | | |  | | | | | | | | | | | | | | | | | | | | | | | | | | | | | | | | | | | | | | | | | | | | | | |  |  |  |  |  |  |  |  |  |  |  |  |  |  |  |  |  |  |  |  |  |  |  |  |  |  |  |  |  |  |  |  |  |  |  |  |  |  |  |  |  |  |  |  |  |  |  |  |  |  |  |  |  |  |  |  |  |  |  |  |  |  |  |  |  |  |  |  |  |  |  |  |  |  |  |  |  |  |  |  |  |  |  |  |  |  |  |  |  |  |  |  |  |  |  |  |  |  |  |  |  |  |  |  |  |  |  |  |  |  |  |  |  |  |  |  |  |  |  |  |  |  |  |  |  |  |  |  |  |  |  |  |  |  |  |  |  |  |  |  |  |  |  |  |  |  |  |  |  |  |  |
| *Streptococcus gallolyticus* | | | | | | | | | | | | | | | | | | | | | | | | | | | | | | | | | | | | | | | | | | | | | | | | | | | | | | | | | L | E | | E | | K | | E | | I | | L | | | L | | | N | | | H | | | G | | | L | | | T | | | | T | | | R | | | G | | | T | | | | T | | | | R | | | | - | | | | | - | | | | - | | | |  | | | | | | | | | | | | | | | | | | | | | | | | | | | | | | | | | | | | | | | | | | | | | | |  |  |  |  |  |  |  |  |  |  |  |  |  |  |  |  |  |  |  |  |  |  |  |  |  |  |  |  |  |  |  |  |  |  |  |  |  |  |  |  |  |  |  |  |  |  |  |  |  |  |  |  |  |  |  |  |  |  |  |  |  |  |  |  |  |  |  |  |  |  |  |  |  |  |  |  |  |  |  |  |  |  |  |  |  |  |  |  |  |  |  |  |  |  |  |  |  |  |  |  |  |  |  |  |  |  |  |  |  |  |  |  |  |  |  |  |  |  |  |  |  |  |  |  |  |  |  |  |  |  |  |  |  |  |  |  |  |  |  |  |  |  |  |  |  |  |  |  |  |  |  |
| *Streptococcus mutans* | | | | | | | | | | | | | | | | | | | | | | | | | | | | | | | | | | | | | | | | | | | | | | | | | | | | | | | | | L | E | | E | | K | | E | | I | | V | | | L | | | N | | | H | | | G | | | I | | | R | | | | E | | | R | | | G | | | T | | | | T | | | | K | | | | - | | | | | - | | | | - | | | |  | | | | | | | | | | | | | | | | | | | | | | | | | | | | | | | | | | | | | | | | | | | | | | |  |  |  |  |  |  |  |  |  |  |  |  |  |  |  |  |  |  |  |  |  |  |  |  |  |  |  |  |  |  |  |  |  |  |  |  |  |  |  |  |  |  |  |  |  |  |  |  |  |  |  |  |  |  |  |  |  |  |  |  |  |  |  |  |  |  |  |  |  |  |  |  |  |  |  |  |  |  |  |  |  |  |  |  |  |  |  |  |  |  |  |  |  |  |  |  |  |  |  |  |  |  |  |  |  |  |  |  |  |  |  |  |  |  |  |  |  |  |  |  |  |  |  |  |  |  |  |  |  |  |  |  |  |  |  |  |  |  |  |  |  |  |  |  |  |  |  |  |  |  |  |
| *Streptococcus thermophilus* | | | | | | | | | | | | | | | | | | | | | | | | | | | | | | | | | | | | | | | | | | | | | | | | | | | | | | | | | L | E | | D | | K | | D | | V | | I | | | L | | | N | | | H | | | G | | | L | | | T | | | | L | | | R | | | Q | | | S | | | | T | | | | K | | | | - | | | | | - | | | | - | | | |  |  |  |  | |  |  | |  |  |  | |  |  |  |  |  |  |  |  |  |  |  |  |  | |  |  | |  |  |  | |  |  |  |  |  |  | |  |  |  | |  |  |  |  | | | | | | | | | | | | | | | | | | | | | | | | | | | | | | | | | | | | | | | | | | | | | | | | | | | | | | | | | | | | | | | | | | | | | | | | | | | | | | | | | | | | | | | | | | | | | | | | | | | | | | | | | | | | | | | | | | | | | | | | | | | | | | | | | | | | | | | | | | | | | | | | | | |  |  |  |  |
