# Supplementary material for: Adaption to glucose limitation is modulated by the pleotropic regulator CcpA, independent of selection pressure strength
Source: BMC Evol Biol. 2019 Jan 10;19:15. doi: 10.1186/s12862-018-1331-x (PMC6327505; doi:10.1186/s12862-018-1331-x)
Supplement: Supplementary file 7 — Table S5. Primers sequences used in this study. (DOCX 18 kb) [file 12862_2018_1331_MOESM7_ESM.docx]

**Additional file 7: Table S5.**Primers sequences used in this study

|  | **primer name** | **primer sequence** | **cre site** | **reference** |
| --- | --- | --- | --- | --- |
| Mobility shift assays | | | | |
|  | ptcB-cre-F1 | AAAAAATAGATAACGCTTGCATTATGAA | AGATAACGCTTGCA | this work |
|  | ptcB-cre-R1 | TTCATAATGCAAGCGTTATCTATTTTTT |  |  |
|  | ptcB-cre-F2 | TGACCACGCAACTCCCAGTA | TGACCACGCAACT | Kowalczyk *et al.,*2007 |
|  | ptcB-cre-R2 | TACTGGGAGTTGCGTGGTCA |  |  |
|  | syn-cre-F | TTCTTACTGTTAGCGCTTTCAGTACG | TGTTAGCGCTTTCA | Schumacher *et al.,*2011 |
|  | syn-cre-R | CGTACTGAAAGCGCTAACAGTAAGAA |  |  |
|  | codYoppD-F | TTTATTTCGCGTAATGTTCAGAAAATTCATGAACATA |  | this work |
|  | codYoppD-R | TATGTTCATGAATTTTCTGAACATTACGCGAAATAAA |  |  |
|  | mtlD-F | ACCACAATGTAAAAGCTTACAATTGCT | TGTAAAAGCTTACA |  |
|  | mtlD-R | AGCAATTGTAAGCTTTTACATTGTGGT |  |  |
| molecular dynamics | | | | |
|  | syn-cre-F | TTCTTACTGTTAGCGCTTTCAGTACG | TGTTAGCGCTTTCA | Schumacher *et al.,*2011 |
|  | dev-cre-F | TTCTTACTGTTAGCGCTTTAAGTACG | TGTTAGCGCTTTAA | this work |
|  | ptcB-cre-F1 | AAAAAATAGATAACGCTTGCATTATGAA | AGATAACGCTTGCA | this work |
|  | *mtlD* | ACCACAATGTAAAAGCTTACAATTGCT | TGTAAAAGCTTACA | this work/Zomer *et al.,*2007 |
|  | *mtlA* | GTCTTATTGGTAGCGGTTATAATAT | TGGTAGCGGTTATA | this work/Zomer *et al.,*2007 |
|  | ackA2 | TTCTTATTGTAAGCGTTATCAATACG | TGTAAGCGTTATCA | Schumacher *et al.,*2011 |
|  | gntR-down | GTCTGATTGAAAGCGGTACCATTTTA | TGAAAGCGGTACCA | Schumacher *et al.,*2011 |
| Pyrosequencing | | | | |
|  | ccpA-pyro-F2 | CAATTTATGATGTGGCACG | N/A | this work |
|  | ccpA-pyro-R1 | ACCGTTAGTCGCGTTGTGAA | N/A |  |
|  | ccpA-pyro-S1 | GTCGCCGGAGTCTCA | N/A |  |
| Primers for resequencing | | | | |
|  |  |  |  |  |
|  | ccpAF | AACACTATATATCCATTCTACG | N/A | this work |
|  | ccpArev2 | TTGCGACTTTTCTTGTAAATG | N/A |  |
|  | codYF | CTAGACCACCATGGGGCATCACCATCACCATCACGTGGCTACATTACTTGAAAAAACACG | N/A |  |
|  | codYR | CTAGTCTAGATTAGAAATTACGTCCAGCAAGTTTATC | N/A |  |
|  | hllAF | ACTGGGCTAAGATTTGC | N/A |  |
|  | hllAR | AACGTGGCACTTGATG | N/A |  |
|  | greAF | ATGTAACAACTGGTAAAG | N/A |  |
|  | greAR | TCACTACAACTCACGGCTTTC | N/A |  |
|  | llrFF | CTTTTCAGCCAATTTAACC | N/A |  |
|  | llrFR | TAAACTGACCGAAGTCAC | N/A |  |
|  | rpoCF | GCTACATCTGAGTACCTTG | N/A |  |
|  | rpoCR | GATTTGCAAGGAGATAATAAGTC | N/A |  |
|  | xptF | TTCGGCTCTTCATGTTTG | N/A |  |
|  | xptR | GTTACTTTTGATACTCTAGC | N/A |  |
|  | llmg0677F | TAAATTATACAATAGTGACTC | N/A |  |
|  | llmg0677R | CAATGATACGAGCTAACC | N/A |  |
|  | llmg1597F | TCCTAACCTAGCTTTTAACG | N/A |  |
|  | llmg1597R | TAGCACGACTTTAAAAATAATC | N/A |  |
